# Cost analysis of childhood hematopoietic stem cell transplantation in Sichuan, China

**DOI:** 10.3389/fpubh.2023.990181

**Published:** 2023-03-23

**Authors:** Min Chen, Yantao Liu, Xue Yang, Yu Hong, Jiaqi Ni

**Affiliations:** ^1^Department of Pharmacy, West China Second University Hospital, Sichuan University, Chengdu, China; ^2^Evidence-Based Pharmacy Center, West China Second University Hospital, Sichuan University, Chengdu, China; ^3^Key Laboratory of Birth Defects and Related Diseases of Women and Children, Sichuan University, Ministry of Education, Chengdu, China; ^4^Department of Pediatric Hematology and Oncology, West China Second University Hospital, Sichuan University, Chengdu, China; ^5^West China School of Pharmacy, Sichuan University, Chengdu, China

**Keywords:** hematopoietic stem cell transplantation, cost analysis, children, China, developing country

## Abstract

**Objective:**

To analyze the inpatient cost of hematopoietic stem cell transplantation (HSCT) in children, so as to support clinical decision-making.

**Methods:**

Study population were children who received HSCT in a tertiary children’s hospital (Sichuan, China) between 1st January 2020 and 31st December 2021. The median and interquartile range (IQR) of total cost at 100 days post transplantation were calculated. Subgroup analyses were conducted based on age, gender, transplantation types, and post-transplant complications. The cost differences between subgroups were analyzed to determine whether it had an impact on the total costs.

**Results:**

A total of 142 pediatric patients were included in the study with a total cost of 250721.78 yuan (197019.16–315740.52, 1 yuan equals to around 0.15 US dollars). Drug costs accounted for 51.85% of the total cost, followed by medical service costs (12.57%) and treatment expenses (12.24%). In terms of transplantation types, the cost of autologous transplantation was lower than that of allogeneic transplantation (115722.98 yuan vs. 256043.99 yuan, *p* < 0.05), and the cost of human leukocyte antigen (HLA) complete matched was lower compared with that of partial matched (213760.88 yuan vs. 294044.84 yuan, *p* < 0.05). As for post-transplant complications, cases with <3 types of complications cost less than those with ≥3 types (212893.25 yuan vs. 286064.60 yuan, *p* < 0.05), and those with severity ≤ grade 2 cost less than those > grade 2 (235569.37 yuan vs. 280061.58 yuan, *p* < 0.05). Age and gender of patients did not lead to statistical differences in the total cost, while the transplantation types and post-transplant complications influenced the total cost.

**Conclusion:**

The total cost at 100 days post transplantation associated with HSCT treatment were substantial for pediatric patients. The HLA compatibility between donors and recipients, and post-transplant complications were important factors affecting the total cost.

## 1. Introduction

Hematopoietic stem cell transplantation (HSCT) is the procedure conducted after completely or partially destroyed the patient’s hematopoietic and immune systems with chemotherapy and / or radiological procedure, then donors’ or autologous healthy hematopoietic stem cells are transplanted to rebuild the patient’s hematopoietic and immune functions (1–3). With the advancement of supportive care and pre-transplant procedures, HSCT has become an effective treatment for hematological diseases such as lymphoma, multiple myeloma and leukemia, and non-hematological diseases such as genetic diseases (4).

HSCT is a significant financial burden for most families, which impacts the quality of life of the whole family (5). Previous studies revealed that many factors affected the total cost of HSCT, which included transplantation types, length of hospitalization, and pre-transplant treatments, etc. (6) Graft vs. host disease (GvHD), infection, disease recurrence and other post-transplant complications led to increased inpatient costs and even the failure of transplantation (6–8). Severe sepsis, acute respiratory failure, endotracheal intubation, total parenteral nutrition (TPN) could double the total cost (8).

A comprehensive search was conducted in databases including Cochrane Library, PubMed, Embase, NHS EED, HTA, CBM, CNKI, Wanfang, and VIP. Literature related to HSCT mainly came from the United States, Japan, Germany and other developed countries. Among them, only four studies were conducted in pediatric patients (9–12). In China, to the best of our knowledge, very limited data has been published on costs associated with HSCT in pediatric patients. The aim of this study is to fill this gap by investigating costs associated with HSCT based on claims data from a Chinese study population, so as to support clinical decision-making and health administrative regulations.

## 2. Methods

### 2.1. Study design

Data was obtained from the electronic medical record system of a tertiary children’s hospital (Sichuan, China). All pediatric patients who received HSCT between 1st January 2020 and 31st December 2021 were included. Literature reported that the direct medical costs of HSCT peaked in the first quarter post transplantation. The costs of 100 days post transplantation accounted for 75% of the total cost within 1 year post transplantation (12, 13). Therefore, the data collection time was the period after initial hospitalization to 100 days post transplantation. Patients with incomplete or missed medical records were excluded.

Medical records were retrospectively reviewed for patients’ demographic data (medical registration number, name, gender, age), admission date, discharge date, date of engraftment, duration of hospital stay, main diagnosis and inpatient expenses. The components of inpatient expenses included medical services, treatments and operations, nursing services, laboratory and diagnostic services, medications, blood products, etc.

### 2.2. Statistical analysis

Excel 2013 was adopted for data entry and classification, and SPSS 17.0 was used for statistical analysis. Cost data was classified into subgroups based on the patient’s medical information (age, gender, transplantation types, post-transplant complications, etc.), and the cost differences between subgroups were analyzed to determine whether it had an impact on the total cost of HSCT.

Shapiro–Wilk Normality test was performed on the data of each subgroup, and the data did not conform to the normal distribution if *p* < 0.05. Independent sample *t*-test was used for the data conforming to the normal distribution, and independent sample nonparametric test was used for the data not conforming to the normal distribution to compare the differences between subgroups. *p* < 0.05 represented statistically significant differences between subgroups.

## 3. Results

### 3.1. Patient and transplant characteristics

A total of 1,322 hospital admissions with 208 pediatric patients were initially retrieved. After removing cases with incomplete medical records, 142 patients were finally included in the study, with 86 boys and 56 girls. The median age was 5 years old, and the median length of hospital stay post transplantation was 51 [43–63] days. In terms of disease types, 14 types were obtained. Leukemia was the most common type of disease receiving HSCT, which included 36 cases of acute myeloid leukemia, 23 cases of acute lymphoblastic leukemia, 7 cases of mixed leukemia, and 6 cases of chronic myeloid leukemia. Anemia was the second common type of disease receiving HSCT with 45 cases, including 24 cases of aplastic anemia and 21 cases of thalassemia. Other malignant diagnoses included solid tumors, leukodystrophy, congenital keratosis, lymphoproliferative diseases, mucopolysaccharide storage diseases, myelodysplastic syndromes, platelet dysfunction, and immune deficiencies with increased IgM. Related information including transplantation types, post-transplant complications and severity classification was shown in Table 1.

**Table 1:** Patient and transplant characteristics.

Total sample	*N* = 142
Median age, years	5 (3–10)
Gender	
Male, *n* (%)	86 (60.6)
Female, *n* (%)	56 (39.4)
Diagnosis	*n* (%)
Acute myeloid leukemia	36 (25.4)
Aplastic anemia	24 (17.0)
Acute lymphoblastic leukemia	23 (16.2)
Thalassemia	21 (14.8)
Solid tumors	8 (5.6)
Mixed leukemia	7 (4.9)
Chronic myeloid leukemia	6 (4.2)
Leukodystrophy	4 (2.8)
Congenital keratosis	3 (2.1)
Lymphoproliferative diseases	3 (2.1)
Mucopolysaccharide storage diseases	2 (1.4)
Myelodysplastic syndromes	2 (1.4)
Platelet dysfunction	2 (1.4)
Immune deficiencies with increased IgM	1 (0.7)
Transplantation types	*n* (%)
Autologous	7 (4.9)
Allogeneic	135 (95.1)
HLA complete matched	72 (53.3)
HLA partial matched	63 (46.7)
Post-transplant complications	*n* (%)
≥3 types	77 (54.2)
<3 types	65 (45.8)
>grade 2	46 (32.4)
≤grade 2	96 (67.6)

HLA, Human leukocyte antigen.

### 3.2. Cost analysis

Direct health care costs were divided into 9 categories: (1) total costs; (2) medical service costs; (3) treatment expenses (surgical and non-surgical methods); (4) nursing service fees; (5) laboratory and diagnostic costs; (6) drug costs; (7) supportive care expenses (including blood transfusion fees, albumin fees, globulin fees, coagulation factor fees, cytokine fees, etc.); (8) material fees; (9) others. The costs were tested for normality, and the results showed that all the costs did not conform to the normal distribution (*p* < 0.05). The total cost of HSCT was 250721.78 yuan (197019.16−315740.52, 1 yuan equals to around 0.15 US dollars). Drug costs accounted for 51.85% of the total cost, followed by medical service costs (12.57%) and treatment expenses (12.24%; Table 2).

**Table 2:** Direct health care costs with hematopoietic stem cell transplantation.

Cost categories	Median (IQR)	Percentage
Total costs	250721.78 (197019.16–315740.52)	100
Drug costs	126688.15 (97940.92–167379.25)	51.85
Medical service costs	32781.25 (28436.50–38891.00)	12.57
Treatment expenses	31714.40 (25537.78–39720.45)	12.24
Laboratory and diagnostic costs	18245.50 (15856.00–22449.75)	7.87
Nursing service fees	15220.90 (10119.00–23945.10)	6.47
Supportive care expenses	11802.50 (8260.00–18445.00)	5.66
Material fees	8098.23 (6212.99–9841.22)	3.15
Others	456.00 (380.00–529.25)	0.18

IQR, interquartile range.

### 3.3. Subgroup analysis

Cost data was classified into subgroups based on the patient’s age, gender, transplantation types, and post-transplant complications. The cost differences between subgroups were analyzed to determine how these factors affected the total cost of HSCT.

#### 3.3.1. Age and gender

Results revealed no significant differences of the costs between the 1- to 5-year-old and the 6- to16-year-old groups. Same results were observed between the male and female groups (*p* > 0.05), which indicated that age and gender did not influence the cost of HSCT (Table 3).

**Table 3:** Subgroup analysis of costs with hematopoietic stem cell transplantation.

	Total costs	Drug costs	Medical service costs	Treatment expenses	Laboratory and diagnostic costs	Nursing service fees	Supportive care expenses	Material fees	Others
**Age, years**									
1–5	218592.22	102095.37	32660.25	31714.40	17906.50	14997.60	11090.00	7925.70	451.00
6–16	270770.83	147811.08	32950.25	31935.90	15520.50	15903.90	12780.00	8462.99	458.00
*P*-value	0.092	0.081	0.590	0.463	0.336	0.340	0.070	0.207	0.662
**Gender**									
Male	244974.49	124564.35	32501.00	31714.40	18245.50	13406.80	11700.00	8017.71	456.50
Female	263957.94	135108.33	33046.40	32441.75	18292.00	16478.40	11802.50	8254.44	440.50
*P*-value	0.440	0.345	0.254	0.907	0.830	0.178	0.889	0.520	0.743
**Transplantation types**									
Allogeneic	256043.99	128600.72	33052.50	32496.70	18367.00	15254.00	11920.00	8218.86	256043.99
Autologous	115722.98	45116.52	23606.00	16162.80	11221.00	14814.60	5920.00	4657.40	209.00
*P*-value	0.001	0.000	0.003	0.001	0.007	0.012	0.011	0.003	0.004
**HLA compatibility of allogeneic HSCT**									
Complete matched	294044.84	156234.97	34416.00	37215.50	20819.00	17730.00	12860.00	9042.54	491.00
Partial matched	213760.88	104317.85	30918.00	27101.60	17200.00	14252.40	11140.00	6969.15	416.00
*P*-value	0.000	0.000	0.001	0.000	0.000	0.027	0.043	0.000	0.000
**Post-transplant complications**									
≥3 types	286064.60	143615.51	34476.00	35376.30	20328.00	18115.00	12060.00	8769.45	492.00
<3 types	212893.25	104317.85	29534.00	28130.10	17040.00	13438.00	10520.00	6899.95	405.00
*P*-value	0.000	0.001	0.000	0.000	0.001	0.007	0.048	0.000	0.000
>grade 2	280061.58	141748.37	34493.50	35311.05	20833.00	19092.50	15220.00	8820.89	484.50
≤grade 2	235569.37	112968.33	31818.00	30508.75	17852.50	13800.30	10480.00	7900.01	436.00
*P*-value	0.004	0.009	0.008	0.032	0.007	0.025	0.002	0.009	0.028

HLA, Human leukocyte antigen.

#### 3.3.2. Transplantation types

Subgroup analysis in different transplantation types showed statistically significant differences in the costs between allogeneic hematopoietic stem cell transplantation (allogeneic HSCT) and autologous hematopoietic stem cell transplantation (autologous HSCT). In addition, human leukocyte antigen (HLA) fully matched and partially matched groups revealed similar differences (*p* < 0.05). The cost of autologous HSCT was lower than that of allogeneic HSCT, and the cost of HLA fully matched HSCT was lower than that of partially matched among allogeneic HSCT (Table 3).

#### 3.3.3. Post-transplant complications

In terms of complications after HSCT, 129 (90.8%) cases had infections, 87 (61.3%) cases experienced oral mucosal ulcer, 69 (48.6%) cases reported engraftment syndrome, 50 (35.2%) cases reported acute GvHD, 27 (19.1%) cases had hemorrhagic cystitis, 2 (1.4%) cases experienced hepatic portal vein occlusion, and 2 (1.4%) cases had graft dysfunction.

Subgroup analysis of different post-transplant complications revealed statistically significant differences in the costs between patients experienced ≥3 types of complications and those with <3. Similar results were observed in patients reported complication severity > grade 2 and those ≤grade 2. Complications were graded based on NCI Common Terminology Criteria for Adverse Events (CTCAE, version 4.03; Supplementary material) grading scale (14) and the Mount Sinai Acute GvHD International Consortium (MAGIC) grading system (Supplementary Figure 1) (15, 16). If the complication fell into inconsistent grade between the two grading scale, it was classified according to the higher level. The more types of complications experienced, and the more severe complications were, the higher the cost of HSCT (Table 3).

## 4. Discussion

HSCT is an effective treatment for a variety of hematological and non-hematological diseases (9). The direct medical costs within 100 days after HSCT treatment in 142 Chinese children were analyzed for the first time. Moreover, the effects of age, gender, transplantation types, and post-transplant complications on the costs were discussed in subgroups. The total cost was 250721.78 yuan (197019.16−315740.52), of which the drug costs were 126688.15 yuan (97940.92−167379.25), followed by medical service costs of 32781.25 yuan (28436.50–38891.00) and treatment expenses of 31714.40 yuan (25537.78−39720.45). In China, the *per capita* GDP in 2021 was 80,976 yuan, and the transplantation cost exceeded 3 times of the *per capita* GDP, which was a substantial financial burden for most families. Drug costs accounted for the highest proportion in HSCT cost, which was consistent with findings from other published literature (6, 13, 17). A study including 162 patients who received HSCT in India showed that age and gender did not affect the cost, which was confirmed by our study (6). Moreover, our study revealed that the transplantation types and post-transplant complications would cause differences in the total cost.

Hematopoietic stem cells mainly origin from bone marrow, peripheral blood, and umbilical cord blood. Based on the source of donors, HSCT can be categorized as autologous HSCT and allogeneic HSCT. Allogeneic HSCT can be further categorized as relative-related and non-relative related allogeneic HSCT based on the relationship between donors and recipients. According to compatibility of HLA between donors and recipients, relative-related allogeneic HSCT can be further categorized as haploid allogeneic HSCT and homozygous allogeneic HSCT (1). Limited studies were conducted regarding the costs of different transplantation types. Kanate et al. performed a retrospective study to evaluate the costs of double umbilical cord blood transplants (dUCBT) and haploid allogeneic HSCT for adult patients in the first 100 days post transplantation. Haploid allogeneic HSCT showed a significantly lower total costs compared with dUCBT (18). A study conducted in Nepal reported the costs were $5,200, $10,000, and $13,300 for autologous HSCT, homozygous allogeneic HSCT, and haploid allogeneic HSCT, respectively (19). Compared with allogeneic HSCT, autologous HSCT has fewer restrictions on age, fewer post-transplant complications, faster recovery and higher quality of life, and transplantation is not limited by donors (4, 20). Among the 142 patients included in this study, only 7 cases (4.9%) received autologous HSCT, and 135 cases (95.1%) received allogeneic HSCT. Compared with developed countries, the proportion of autologous HSCT was relatively low (4). The cost of autologous HSCT is lower than that of allogeneic HSCT (115722.98 yuan vs. 256043.99 yuan). In addition, the cost of HLA fully matched HSCT was lower than that of partially matched (213760.88 yuan vs. 294044.84 yuan).

The occurrence of post-transplant complications is one of the main reasons for the high cost of HSCT. A retrospective study of 1,831 adult patients who received HSCT in the United States showed that 70% patients experienced complications. The most common complications were mucositis, neutropenia with fever, and infections. The incidence of acute organ failure, acute GvHD and death was relatively low (10%), however, it greatly increased inpatient cost and length of hospital stay (21). In contrast, another study showed that acute GvHD within 2 months after transplantation did not increase the cost (22). In this study, 97.2% pediatric patients experienced post-transplant complications. The incidence of infection, oral mucositis, engraftment syndrome and acute GvHD was relatively high (90.8%, 61.3%, 48.6%, and 35.2%, respectively). Among them, 77 patients (54.2%) were complicated with ≥3 types of complications. The cost of HSCT in those with ≥3 complications increased by around 34% compared with those with <3 complications (286064.60 vs. 212893.25 yuan). The cost of HSCT with complication severity > grade 2 increased by about 19% compared with those ≤grade 2 (280061.58 vs. 235569.37 yuan), which indicated that the increase in the types and severity of complications led to an increase in the cost.

Some limitations were identified in the study. The sample size was relatively small with 142 patients. In addition, we focused on direct medical expenses during hospitalization. Outpatient visits and non-medical expenses, including transportation and accommodation expenses were not discussed. Moreover, the collection time period was 100 days after transplantation, which resulted in the lack of long-term follow-up data. Further research using larger and randomized controlled trials will be required to confirm the long-term cost. Studies showed the disease types, such as acute leukemia and aplastic anemia, and pre-transplant treatments would affect the cost (23). Hou et al. reported that low dose cyclophosphamide (CTX) combined with thoracic and abdominal radiotherapy improved the efficacy without increasing the incidence of HSCT complications. In contrast, large dose of CTX was related to the high incidence of GvHD. Adding anti-thymocyte globulin on the basis of the original regimen may reduce the incidence of GvHD (24). A retrospective study performed in pediatric HSCT patients revealed that the risk of pneumatosis intestinalis increased in patients receiving steroid therapy, patients with gastrointestinal GvHD, and patients relying on nasogastric tube feeds for >50% of total daily nutrition (25). Yang et al. reported that patients with mismatched blood type, CMV infection and elderly age had an increased risk of poor graft function post HSCT (26). Our study did not conduct the subgroup analyses based on these factors due to the limited sample size, which demanded further investigation.

## 5. Conclusion

Hematopoietic stem cell transplantation in children is an expensive health-care intervention. Allogeneic HSCT (especially those with HLA partial matched), and more types of post-transplant complications will result in an increase in the total cost. Selection of proper donor, adoption of appropriate immunotherapy post transplantation, preventing infection, and early transition to outpatient visits may be worthwhile to reduce the total cost.

## Data availability statement

The raw data supporting the conclusions of this article will be made available by the authors, without undue reservation.

## Ethics statement

The studies involving human participants were reviewed and approved by West China Second University Hospital Ethical Committee. Written informed consent to participate in this study was provided by the participants’ legal guardian/next of kin.

## Author contributions

MC and JN: study design and protocol preparation and manuscript drafting. YL: data collection. YH: data analysis. XY, JN, and MC: data interpretation. All authors contributed to the article and approved the submitted version.

## Conflict of interest

The authors declare that the research was conducted in the absence of any commercial or financial relationships that could be construed as a potential conflict of interest.

## Publisher’s note

All claims expressed in this article are solely those of the authors and do not necessarily represent those of their affiliated organizations, or those of the publisher, the editors and the reviewers. Any product that may be evaluated in this article, or claim that may be made by its manufacturer, is not guaranteed or endorsed by the publisher.
