# Supplementary material for: Cost analysis of childhood hematopoietic stem cell transplantation in Sichuan, China
Source: Front Public Health. 2023 Mar 23;11:990181. doi: 10.3389/fpubh.2023.990181 (PMC10076710; doi:10.3389/fpubh.2023.990181)
Supplement: Supplementary file 1 [file Data_Sheet_1.PDF]

Common Terminology Criteria for Adverse Events (CTCAE)

Version 4.0

Published: May 28, 2009 (v4.03: June 14, 2010)

U.S.DEPARTMENT OF HEALTH AND HUMAN SERVICES

National Institutes of Health

National Cancer Institute

Common Terminology Criteria for Adverse Events v4.0 (CTCAE)

Publish Date: May 28, 2009

Quick Reference

The NCI Common Terminology Criteria for Adverse Events is a descriptive terminology which can be utilized for Adverse Event (AE) reporting. A grading (severity) scale is provided for each AE term.

Components and Organization

SOC

System Organ Class, the highest level of the MedDRA hierarchy, is identified by anatomical or physiological system, etiology, or purpose (e.g., SOC Investigations for laboratory test results). CTCAE terms are grouped by MedDRA Primary SOCs. Within each SOC, AEs are listed and accompanied by descriptions of severity (Grade).

CTCAE Terms

An Adverse Event (AE) is any unfavorable and unintended sign (including an abnormal laboratory finding), symptom, or disease temporally associated with the use of a medical treatment or procedure that may or may *not* be considered related to the medical treatment or procedure. An AE is a term that is a unique representation of a specific event used for medical documentation and scientific analyses. Each CTCAE v4.0 term is a MedDRA LLT (Lowest Level Term).

Definitions

A brief definition is provided to clarify the meaning of each AE term.

Grades

Grade refers to the severity of the AE. The CTCAE displays Grades 1 through 5 with unique clinical descriptions of severity for each AE based on this general guideline:

Grade 1	Mild; asymptomatic or mild symptoms; clinical or diagnostic observations only; intervention not indicated.
Grade 2	Moderate; minimal, local or noninvasive intervention indicated; limiting age-appropriate instrumental ADL*.
Grade 3	Severe or medically significant but not immediately life-threatening; hospitalization or prolongation of hospitalization indicated; disabling; limiting self care ADL**.
Grade 4	Life-threatening consequences; urgent intervention indicated.
Grade 5	Death related to AE.

A Semi-colon indicates ‘or’ within the description of the grade.

A single dash (-) indicates a grade is not available.

Not all Grades are appropriate for all AEs. Therefore, some AEs are listed with fewer than five options for Grade selection.

Grade 5

Grade 5 (Death) is not appropriate for some AEs and therefore is not an option.

Activities of Daily Living (ADL)

*Instrumental ADL refer to preparing meals, shopping for groceries or clothes, using the telephone, managing money, etc.

**Self care ADL refer to bathing, dressing and undressing, feeding self, using the toilet, taking medications, and not bedridden.

---

† CTCAE v4.0 incorporates certain elements of the MedDRA terminology. For further details on MedDRA refer to the MedDRA MSSO Web site (http://www.meddramsso.com).

SOC	Page
Blood and lymphatic system disorders	3
Cardiac disorders	6
Congenital, familial and genetic disorders	15
Ear and labyrinth disorders	16
Endocrine disorders	19
Eye disorders	22
Gastrointestinal disorders	28
General disorders and administration site conditions	55
Hepatobiliary disorders	61
Immune system disorders	65
Infections and infestations	68
Injury, poisoning and procedural complications	87
Investigations	107
Metabolism and nutrition disorders	114
Musculoskeletal and connective tissue disorders	119
Neoplasms benign, malignant and unspecified (incl cysts and polyps)	128
Nervous system disorders	129
Pregnancy, puerperium and perinatal conditions	141
Psychiatric disorders	142
Renal and urinary disorders	147
Reproductive system and breast disorders	153
Respiratory, thoracic and mediastinal disorders	164
Skin and subcutaneous tissue disorders	179
Social circumstances	188
Surgical and medical procedures	189
Vascular disorders	190

Blood and lymphatic system disorders

	Grade				
**Adverse Event**	**1**	**2**	**3**	**4**	**5**
Anemia	Hemoglobin (Hgb) <LLN - 10.0 g/dL; <LLN - 6.2 mmol/L; <LLN - 100 g/L	Hgb <10.0 - 8.0 g/dL; <6.2 - 4.9 mmol/L; <100 - 80g/L	Hgb <8.0 g/dL; <4.9 mmol/L; <80 g/L; transfusion indicated	Life-threatening consequences; urgent intervention indicated	Death
Definition: A disorder characterized by an reduction in the amount of hemoglobin in 100 ml of blood. Signs and symptoms of anemia may include pallor of the skin and mucous membranes, shortness of breath, palpitations of the heart, soft systolic murmurs, lethargy, and fatigability.					
Bone marrow hypocellular	Mildly hypocellular or <=25% reduction from normal cellularity for age	Moderately hypocellular or >25 - <50% reduction from normal cellularity for age	Severely hypocellular or >50 - <=75% reduction cellularity from normal for age	Aplastic persistent for longer than 2 weeks	Death
Definition: A disorder characterized by the inability of the bone marrow to produce hematopoietic elements.					
Disseminated intravascular coagulation	-	Laboratory findings with no bleeding	Laboratory findings and bleeding	Life-threatening consequences; urgent intervention indicated	Death
Definition: A disorder characterized by systemic pathological activation of blood clotting mechanisms which results in clot formation throughout the body. There is an increase in the risk of hemorrhage as the body is depleted of platelets and coagulation factors.					
Febrile neutropenia	-	-	ANC <1000/mm3 with a single temperature of >38.3 degrees C (101 degrees F) or a sustained temperature of >=38 degrees C (100.4 degrees F) for more than one hour.	Life-threatening consequences; urgent intervention indicated	Death
Definition: A disorder characterized by an ANC <1000/mm3 and a single temperature of >38.3 degrees C (101 degrees F) or a sustained temperature of >=38 degrees C (100.4 degrees F) for more than one hour.					

Blood and lymphatic system disorders

	Grade				
**Adverse Event**	**1**	**2**	**3**	**4**	**5**
Hemolysis	Laboratory evidence of hemolysis only (e.g., direct antiglobulin test; DAT; Coombs'; schistocytes; decreased haptoglobin)	Evidence of hemolysis and >=2 gm decrease in hemoglobin.	Transfusion or medical intervention indicated (e.g., steroids)	Life-threatening consequences; urgent intervention indicated	Death
Definition: A disorder characterized by laboratory test results that indicate widespread erythrocyte cell membrane destruction.					
Hemolytic uremic syndrome	Evidence of RBC destruction (schistocytosis) without clinical consequences	-	Laboratory findings with clinical consequences (e.g., renal insufficiency, petechiae)	Life-threatening consequences, (e.g., CNS hemorrhage or thrombosis/embolism or renal failure)	Death
Definition: A disorder characterized by a form of thrombotic microangiopathy with renal failure, hemolytic anemia, and severe thrombocytopenia.					
Leukocytosis	-	-	>100,000/mm3	Clinical manifestations of leucostasis; urgent intervention indicated	Death
Definition: A disorder characterized by laboratory test results that indicate an increased number of white blood cells in the blood.					
Lymph node pain	Mild pain	Moderate pain; limiting instrumental ADL	Severe pain; limiting self care ADL	-	-
Definition: A disorder characterized by a sensation of marked discomfort in a lymph node.					
Spleen disorder	Incidental findings (e.g., Howell-Jolly bodies); mild degree of thrombocytosis and leukocytosis	Prophylactic antibiotics indicated	-	Life-threatening consequences; urgent intervention indicated	Death

Blood and lymphatic system disorders					
	Grade				
**Adverse Event**	**1**	**2**	**3**	**4**	**5**
Definition: A disorder of the spleen.					
Thrombotic thrombocytopenic purpura	Evidence of RBC destruction (schistocytosis) without clinical consequences	-	Laboratory findings with clinical consequences (e.g., renal insufficiency, petechiae)	Life-threatening consequences, (e.g., CNS hemorrhage or thrombosis/embolism or renal failure)	Death
Definition: A disorder characterized by the presence of microangiopathic hemolytic anemia, thrombocytopenic purpura, fever, renal abnormalities and neurological abnormalities such as seizures, hemiplegia, and visual disturbances. It is an acute or subacute condition.					
Blood and lymphatic system disorders - Other, specify	Asymptomatic or mild symptoms; clinical or diagnostic observations only; intervention not indicated	Moderate; minimal, local or noninvasive intervention indicated; limiting age-appropriate instrumental ADL	Severe or medically significant but not immediately life-threatening; hospitalization or prolongation of existing hospitalization indicated; disabling; limiting self care ADL	Life-threatening consequences; urgent intervention indicated	Death

Cardiac disorders

	Grade				
**Adverse Event**	**1**	**2**	**3**	**4**	**5**
Acute coronary syndrome	-	Symptomatic, progressive angina; cardiac enzymes normal; hemodynamically stable	Symptomatic, unstable angina and/or acute myocardial infarction, cardiac enzymes abnormal, hemodynamically stable	Symptomatic, unstable angina and/or acute myocardial infarction, cardiac enzymes abnormal, hemodynamically unstable	Death
Definition: A disorder characterized by signs and symptoms related to acute ischemia of the myocardium secondary to coronary artery disease. The clinical presentation covers a spectrum of heart diseases from unstable angina to myocardial infarction.					
Aortic valve disease	Asymptomatic valvular thickening with or without mild valvular regurgitation or stenosis by imaging	Asymptomatic; moderate regurgitation or stenosis by imaging	Symptomatic; severe regurgitation or stenosis by imaging; symptoms controlled with medical intervention	Life-threatening consequences; urgent intervention indicated (e.g., valve replacement, valvuloplasty)	Death
Definition: A disorder characterized by a defect in aortic valve function or structure.					
Asystole	Periods of asystole; non-urgent medical management indicated	-	-	Life-threatening consequences; urgent intervention indicated	Death
Definition: A disorder characterized by a dysrhythmia without cardiac electrical activity. Typically, this is accompanied by cessation of the pumping function of the heart.					
Atrial fibrillation	Asymptomatic, intervention not indicated	Non-urgent medical intervention indicated	Symptomatic and incompletely controlled medically, or controlled with device (e.g., pacemaker), or ablation	Life-threatening consequences; urgent intervention indicated	Death

Cardiac disorders					
	Grade				
**Adverse Event**	**1**	**2**	**3**	**4**	**5**
Definition: A disorder characterized by a dysrhythmia without discernible P waves and an irregular ventricular response due to multiple reentry circuits. The rhythm disturbance originates above the ventricles.					
Atrial flutter	Asymptomatic, intervention not indicated	Non-urgent medical intervention indicated	Symptomatic and incompletely controlled medically, or controlled with device (e.g., pacemaker), or ablation	Life-threatening consequences; urgent intervention indicated	Death
Definition: A disorder characterized by a dysrhythmia with organized rhythmic atrial contractions with a rate of 200-300 beats per minute. The rhythm disturbance originates in the atria.					
Atrioventricular block complete	-	Non-urgent intervention indicated	Symptomatic and incompletely controlled medically, or controlled with device (e.g., pacemaker)	Life-threatening consequences; urgent intervention indicated	Death
Definition: A disorder characterized by a dysrhythmia with complete failure of atrial electrical impulse conduction through the AV node to the ventricles.					
Atrioventricular block first degree	Asymptomatic, intervention not indicated	Non-urgent intervention indicated	-	-	-
Definition: A disorder characterized by a dysrhythmia with a delay in the time required for the conduction of an electrical impulse through the atrioventricular (AV) node beyond 0.2 seconds; prolongation of the PR interval greater than 200 milliseconds.					
Cardiac arrest	-	-	-	Life-threatening consequences; urgent intervention indicated	Death
Definition: A disorder characterized by cessation of the pumping function of the heart.					

Cardiac disorders

	Grade				
**Adverse Event**	**1**	**2**	**3**	**4**	**5**
Chest pain - cardiac	Mild pain	Moderate pain; limiting instrumental ADL	Pain at rest; limiting self care ADL	-	-
Definition: A disorder characterized by substernal discomfort due to insufficient myocardial oxygenation.					
Conduction disorder	Mild symptoms; intervention not indicated	Moderate symptoms	Severe symptoms; intervention indicated	Life-threatening consequences; urgent intervention indicated	Death
Definition: A disorder characterized by pathological irregularities in the cardiac conduction system.					
Constrictive pericarditis	-	-	Symptomatic heart failure or other cardiac symptoms, responsive to intervention	Refractory heart failure or other poorly controlled cardiac symptoms	Death
Definition: A disorder characterized by a thickened and fibrotic pericardial sac; these fibrotic changes impede normal myocardial function by restricting myocardial muscle action.					
Heart failure	Asymptomatic with laboratory (e.g., BNP [B-Natriuretic Peptide]) or cardiac imaging abnormalities	Symptoms with mild to moderate activity or exertion	Severe with symptoms at rest or with minimal activity or exertion; intervention indicated	Life-threatening consequences; urgent intervention indicated (e.g., continuous IV therapy or mechanical hemodynamic support)	Death
Definition: A disorder characterized by the inability of the heart to pump blood at an adequate volume to meet tissue metabolic requirements, or, the ability to do so only at an elevation in the filling pressure.					

Cardiac disorders

	Grade				
**Adverse Event**	**1**	**2**	**3**	**4**	**5**
Left ventricular systolic dysfunction	-	-	Symptomatic due to drop in ejection fraction responsive to intervention	Refractory or poorly controlled heart failure due to drop in ejection fraction; intervention such as ventricular assist device, intravenous vasopressor support, or heart transplant indicated	Death
Definition: A disorder characterized by failure of the left ventricle to produce adequate output despite an increase in distending pressure and in end-diastolic volume. Clinical manifestations mayinclude dyspnea, orthopnea, and other signs and symptoms of pulmonary congestion and edema.					
Mitral valve disease	Asymptomatic valvular thickening with or without mild valvular regurgitation or stenosis by imaging	Asymptomatic; moderate regurgitation or stenosis by imaging	Symptomatic; severe regurgitation or stenosis by imaging; symptoms controlled with medical intervention	Life-threatening consequences; urgent intervention indicated (e.g., valve replacement, valvuloplasty)	Death
Definition: A disorder characterized by a defect in mitral valve function or structure.					
Mobitz (type) II atrioventricular block	Asymptomatic, intervention not indicated	Symptomatic; medical intervention indicated	Symptomatic and incompletely controlled medically, or controlled with device (e.g., pacemaker)	Life-threatening consequences; urgent intervention indicated	Death
Definition: A disorder characterized by a dysrhythmia with relatively constant PR interval prior to the block of an atrial impulse. This is the result of intermittent failure of atrial electrical impulse conduction through the atrioventricular (AV) node to the ventricles.					

Cardiac disorders

	Grade				
**Adverse Event**	**1**	**2**	**3**	**4**	**5**
Mobitz type I	Asymptomatic, intervention not indicated	Symptomatic; medical intervention indicated	Symptomatic and incompletely controlled medically, or controlled with device (e.g., pacemaker)	Life-threatening consequences; urgent intervention indicated	Death
Definition: A disorder characterized by a dysrhythmia with a progressively lengthening PR interval prior to the blocking of an atrial impulse. This is the result of intermittent failure of atrial electrical impulse conduction through the atrioventricular (AV) node to the ventricles.					
Myocardial infarction	-	Asymptomatic and cardiac enzymes minimally abnormal and no evidence of ischemic ECG changes	Severe symptoms; cardiac enzymes abnormal; hemodynamically stable; ECG changes consistent with infarction	Life-threatening consequences; hemodynamically unstable	Death
Definition: A disorder characterized by gross necrosis of the myocardium; this is due to an interruption of blood supply to the area.					
Myocarditis	Asymptomatic with laboratory (e.g., BNP [B-Natriuretic Peptide]) or cardiac imaging abnormalities	Symptoms with mild to moderate activity or exertion	Severe with symptoms at rest or with minimal activity or exertion; intervention indicated	Life-threatening consequences; urgent intervention indicated (e.g., continuous IV therapy or mechanical hemodynamic support)	Death
Definition: A disorder characterized by inflammation of the muscle tissue of the heart.					
Palpitations	Mild symptoms; intervention not indicated	Intervention indicated	-	-	-
Definition: A disorder characterized by an unpleasant sensation of irregular and/or forceful beating of the heart.					

Cardiac disorders					
	Grade				
**Adverse Event**	**1**	**2**	**3**	**4**	**5**
Paroxysmal atrial tachycardia	Asymptomatic, intervention not indicated	Symptomatic; medical management indicated	IV medication indicated	Life-threatening consequences; incompletely controlled medically; cardioversion indicated	Death
Definition: A disorder characterized by a dysrhythmia with abrupt onset and sudden termination of atrial contractions with a rate of 150-250 beats per minute. The rhythm disturbance originates in the atria.					
Pericardial effusion	-	Asymptomatic effusion size small to moderate	Effusion with physiologic consequences	Life-threatening consequences; urgent intervention indicated	Death
Definition: A disorder characterized by fluid collection within the pericardial sac, usually due to inflammation.					
Pericardial tamponade	-	-	-	Life-threatening consequences; urgent intervention indicated	Death
Definition: A disorder characterized by an increase in intrapericardial pressure due to the collection of blood or fluid in the pericardium.					
Pericarditis	Asymptomatic, ECG or physical findings (e.g., rub) consistent with pericarditis	Symptomatic pericarditis (e.g., chest pain)	Pericarditis with physiologic consequences (e.g., pericardial constriction)	Life-threatening consequences; urgent intervention indicated	Death
Definition: A disorder characterized by irritation to the layers of the pericardium (the protective sac around the heart).					
Pulmonary valve disease	Asymptomatic valvular thickening with or without mild valvular regurgitation or stenosis by imaging	Asymptomatic; moderate regurgitation or stenosis by imaging	Symptomatic; severe regurgitation or stenosis by imaging; symptoms controlled with medical intervention	Life-threatening consequences; urgent intervention indicated (e.g., valve replacement, valvuloplasty)	Death

Cardiac disorders

	Grade				
**Adverse Event**	**1**	**2**	**3**	**4**	**5**
Definition: A disorder characterized by a defect in pulmonary valve function or structure.					
Restrictive cardiomyopathy	-	-	Symptomatic heart failure or other cardiac symptoms, responsive to intervention	Refractory heart failure or other poorly controlled cardiac symptoms	Death
Definition: A disorder characterized by an inability of the ventricles to fill with blood because the myocardium (heart muscle) stiffens and loses its flexibility.					
Right ventricular dysfunction	Asymptomatic with laboratory (e.g., BNP [B-Natriuretic Peptide]) or cardiac imaging abnormalities	Symptoms with mild to moderate activity or exertion	Severe symptoms, associated with hypoxemia, right heart failure; oxygen indicated	Life-threatening consequences; urgent intervention indicated (e.g., ventricular assist device); heart transplant indicated	Death
Definition: A disorder characterized by impairment of right ventricular function associated with low ejection fraction and a decrease in motility of the right ventricular wall.					
Sick sinus syndrome	Asymptomatic, intervention not indicated	Non-urgent intervention indicated	Severe, medically significant; medical intervention indicated	Life-threatening consequences; urgent intervention indicated	Death
Definition: A disorder characterized by a dysrhythmia with alternating periods of bradycardia and atrial tachycardia accompanied by syncope, fatigue and dizziness.					
Sinus bradycardia	Asymptomatic, intervention not indicated	Symptomatic, medical intervention indicated	Severe, medically significant, medical intervention indicated	Life-threatening consequences; urgent intervention indicated	Death
Definition: A disorder characterized by a dysrhythmia with a heart rate less than 60 beats per minute that originates in the sinus node.					
Sinus tachycardia	Asymptomatic, intervention not indicated	Symptomatic; non-urgent medical intervention indicated	Urgent medical intervention indicated	-	-
Definition: A disorder characterized by a dysrhythmia with a heart rate greater than 100 beats per minute that originates in the sinus node.					

Cardiac disorders

	Grade				
**Adverse Event**	**1**	**2**	**3**	**4**	**5**
Supraventricular tachycardia	Asymptomatic, intervention not indicated	Non-urgent medical intervention indicated	Medical intervention indicated	Life-threatening consequences; urgent intervention indicated	Death
Definition: A disorder characterized by a dysrhythmia with a heart rate greater than 100 beats per minute that originates above the ventricles.					
Tricuspid valve disease	Asymptomatic valvular thickening with or without mild valvular regurgitation or stenosis	Asymptomatic; moderate regurgitation or stenosis by imaging	Symptomatic; severe regurgitation or stenosis; symptoms controlled with medical intervention	Life-threatening consequences; urgent intervention indicated (e.g., valve replacement, valvuloplasty)	Death
Definition: A disorder characterized by a defect in tricuspid valve function or structure.					
Ventricular arrhythmia	Asymptomatic, intervention not indicated	Non-urgent medical intervention indicated	Medical intervention indicated	Life-threatening consequences; hemodynamic compromise; urgent intervention indicated	Death
Definition: A disorder characterized by a dysrhythmia that originates in the ventricles.					
Ventricular fibrillation	-	-	-	Life-threatening consequences; hemodynamic compromise; urgent intervention indicated	Death
Definition: A disorder characterized by a dysrhythmia without discernible QRS complexes due to rapid repetitive excitation of myocardial fibers without coordinated contraction of the ventricles.					

Cardiac disorders

	Grade				
**Adverse Event**	**1**	**2**	**3**	**4**	**5**
Ventricular tachycardia	-	Non-urgent medical intervention indicated	Medical intervention indicated	Life-threatening consequences; hemodynamic compromise; urgent intervention indicated	Death
Definition: A disorder characterized by a dysrhythmia with a heart rate greater than 100 beats per minute that originates distal to the bundle of His.					
Wolff-Parkinson-White syndrome	Asymptomatic, intervention not indicated	Non-urgent medical intervention indicated	Symptomatic and incompletely controlled medically or controlled with procedure	Life-threatening consequences; urgent intervention indicated	Death
Definition: A disorder characterized by the presence of an accessory conductive pathway between the atria and the ventricles that causes premature ventricular activation.					
Cardiac disorders - Other, specify	Asymptomatic or mild symptoms; clinical or diagnostic observations only; intervention not indicated	Moderate; minimal, local or noninvasive intervention indicated; limiting age-appropriate instrumental ADL	Severe or medically significant but not immediately life-threatening; hospitalization or prolongation of existing hospitalization indicated; disabling; limiting self care ADL	Life-threatening consequences; urgent intervention indicated	Death

Congenital, familial and genetic disorders					
	Grade				
**Adverse Event**	**1**	**2**	**3**	**4**	**5**
Congenital, familial and genetic disorders - Other, specify	Asymptomatic or mild symptoms; clinical or diagnostic observations only; intervention not indicated	Moderate; minimal, local or noninvasive intervention indicated; limiting age-appropriate instrumental ADL	Severe or medically significant but not immediately life-threatening; hospitalization or prolongation of existing hospitalization indicated; disabling; limiting self care ADL	Life-threatening consequences; urgent intervention indicated	Death

Ear and labyrinth disorders

	Grade				
**Adverse Event**	**1**	**2**	**3**	**4**	**5**
Ear pain	Mild pain	Moderate pain; limiting instrumental ADL	Severe pain; limiting self care ADL	-	-
Definition: A disorder characterized by a sensation of marked discomfort in the ear.					
External ear inflammation	External otitis with erythema or dry desquamation	External otitis with moist desquamation, edema, enhanced cerumen or discharge; tympanic membrane perforation; tympanostomy	External otitis with mastoiditis; stenosis or osteomyelitis; necrosis of soft tissue or bone	Urgent operative intervention indicated	Death
Definition: A disorder characterized by inflammation, swelling and redness to the outer ear and ear canal.					
External ear pain	Mild pain	Moderate pain; limiting instrumental ADL	Severe pain; limiting self care ADL	-	-
Definition: A disorder characterized by a sensation of marked discomfort in the external ear region.					

Ear and labyrinth disorders

	Grade				
**Adverse Event**	**1**	**2**	**3**	**4**	**5**
Hearing impaired	Adults enrolled on a Monitoring Program (on a 1, 2, 3, 4, 6 and 8 kHz audiogram): Threshold shift of 15 - 25 dB averaged at 2 contiguous test frequencies in at least one ear.<br><br>Adults not enrolled in Monitoring Program: subjective change in hearing in the absence of documented hearing loss.<br><br>Pediatric (on a 1, 2, 3, 4, 6 and 8 kHz audiogram): Threshold shift >20 dB at 8 kHz in at least one ear.	Adults enrolled in Monitoring Program (on a 1, 2, 3, 4, 6 and 8 kHz audiogram): Threshold shift of >25 dB averaged at 2 contiguous test frequencies in at least one ear.<br><br>Adults not enrolled in Monitoring Program: hearing loss but hearing aid or intervention not indicated; limiting instrumental ADL.<br><br>Pediatric (on a 1, 2, 3, 4, 6 and 8 kHz audiogram): Threshold shift >20 dB at 4 kHz and above in at least one ear.	Adults enrolled in Monitoring Program (on a 1, 2, 3, 4, 6 and 8 kHz audiogram): Threshold shift of >25 dB averaged at 3 contiguous test frequencies in at least one ear; therapeutic intervention indicated.<br><br>Adults not enrolled in Monitoring Program: hearing loss with hearing aid or intervention indicated; limiting self care ADL.<br><br>Pediatric (on a 1, 2, 3, 4, 6 and 8 kHz audiogram): hearing loss sufficient to indicate therapeutic intervention, including hearing aids; threshold shift >20 dB at 3 kHz and above in at least one ear; additional speech-language related services indicated.	Adults: Decrease in hearing to profound bilateral loss (absolute threshold >80 dB HL at 2 kHz and above); non-servicable hearing.<br><br>Pediatric: Audiologic indication for cochlear implant and additional speech-language related services indicated.	-

Ear and labyrinth disorders

	Grade				
**Adverse Event**	**1**	**2**	**3**	**4**	**5**
Definition: A disorder characterized by partial or complete loss of the ability to detect or understand sounds resulting from damage to ear structures.					
Middle ear inflammation	Serous otitis	Serous otitis, medical intervention indicated	Mastoiditis; necrosis of canal soft tissue or bone	Life-threatening consequences; urgent intervention indicated	Death
Definition: A disorder characterized by inflammation (physiologic response to irritation), swelling and redness to the middle ear.					
Tinnitus	Mild symptoms; intervention not indicated	Moderate symptoms; limiting instrumental ADL	Severe symptoms; limiting self care ADL	-	-
Definition: A disorder characterized by noise in the ears, such as ringing, buzzing, roaring or clicking.					
Vertigo	Mild symptoms	Moderate symptoms; limiting instrumental ADL	Severe symptoms; limiting self care ADL	-	-
Definition: A disorder characterized by a sensation as if the external world were revolving around the patient (objective vertigo) or as if he himself were revolving in space (subjective vertigo).					
Vestibular disorder	-	Symptomatic; limiting instrumental ADL	Severe symptoms; limiting self care ADL	-	-
Definition: A disorder characterized by dizziness, imbalance, nausea, and vision problems.					
Ear and labyrinth disorders - Other, specify	Asymptomatic or mild symptoms; clinical or diagnostic observations only; intervention not indicated	Moderate; minimal, local or noninvasive intervention indicated; limiting age-appropriate instrumental ADL	Severe or medically significant but not immediately life-threatening; hospitalization or prolongation of existing hospitalization indicated; disabling; limiting self care ADL	Life-threatening consequences; urgent intervention indicated	Death

Endocrine disorders

	Grade				
**Adverse Event**	**1**	**2**	**3**	**4**	**5**
Adrenal insufficiency	Asymptomatic; clinical or diagnostic observations only; intervention not indicated	Moderate symptoms; medical intervention indicated	Severe symptoms; hospitalization indicated	Life-threatening consequences; urgent intervention indicated	Death
Definition: A disorder that occurs when the adrenal cortex does not produce enough of the hormone cortisol and in some cases, the hormone aldosterone. It may be due to a disorder of the adrenal cortex as in Addison's disease or primary adrenal insufficiency.					
Cushingoid	Mild symptoms; intervention not indicated	Moderate symptoms; medical intervention indicated	Severe symptoms, medical intervention or hospitalization indicated	-	-
Definition: A disorder characterized by signs and symptoms that resemble Cushing's disease or syndrome: buffalo hump obesity, striations, adiposity, hypertension, diabetes, and osteoporosis, usually due to exogenous corticosteroids.					
Delayed puberty	-	No breast development by age 13 yrs for females; testes volume of <3 cc or no Tanner Stage 2 development by age 14.5 yrs for males	No breast development by age 14 yrs for females; no increase in testes volume or no Tanner Stage 2 by age 16 yrs for males; hormone replacement indicated	-	-
Definition: A disorder characterized by unusually late sexual maturity.					
Growth accelerated	-	>= +2 SD (standard deviation) above mid parental height or target height	-	-	-
Definition: A disorder characterized by greater growth than expected for age.					
Hyperparathyroidism	Mild symptoms; intervention not indicated	Moderate symptoms; medical intervention indicated	-	-	-

Endocrine disorders					
	Grade				
**Adverse Event**	**1**	**2**	**3**	**4**	**5**
Definition: A disorder characterized by an increase in production of parathyroid hormone by the parathyroid glands. This results in hypercalcemia (abnormally high levels of calcium in the blood).					
Hyperthyroidism	Asymptomatic; clinical or diagnostic observations only; intervention not indicated	Symptomatic; thyroid suppression therapy indicated; limiting instrumental ADL	Severe symptoms; limiting self care ADL; hospitalization indicated	Life-threatening consequences; urgent intervention indicated	Death
Definition: A disorder characterized by excessive levels of thyroid hormone in the body. Common causes include an overactive thyroid gland or thyroid hormone overdose.					
Hypoparathyroidism	Asymptomatic; clinical or diagnostic observations only; intervention not indicated	Moderate symptoms; medical intervention indicated	Severe symptoms; medical intervention or hospitalization indicated	Life-threatening consequences; urgent intervention indicated	Death
Definition: A disorder characterized by a decrease in production of parathyroid hormone by the parathyroid glands.					
Hypothyroidism	Asymptomatic; clinical or diagnostic observations only; intervention not indicated	Symptomatic; thyroid replacement indicated; limiting instrumental ADL	Severe symptoms; limiting self care ADL; hospitalization indicated	Life-threatening consequences; urgent intervention indicated	Death
Definition: A disorder characterized by a decrease in production of thyroid hormone by the thyroid gland.					
Precocious puberty	Physical signs of puberty with no biochemical markers for females <8 years and males <9 years	Physical signs and biochemical markers of puberty for females <8 years and males <9 years	-	-	-
Definition: A disorder characterized by unusually early development of secondary sexual features; the onset of sexual maturation begins usually before age 8 for girls and before age 9 for boys.					
Virilization	Mild symptoms; intervention not indicated	Moderate symptoms; medical intervention indicated	-	-	-

Endocrine disorders

	Grade				
**Adverse Event**	**1**	**2**	**3**	**4**	**5**
Definition: A disorder characterized by inappropriate masculinization occurring in a female or prepubertal male.					
Endocrine disorders - Other, specify	Asymptomatic or mild symptoms; clinical or diagnostic observations only; intervention not indicated	Moderate; minimal, local or noninvasive intervention indicated; limiting age-appropriate instrumental ADL	Severe or medically significant but not immediately life-threatening; hospitalization or prolongation of existing hospitalization indicated; disabling; limiting self care ADL	Life-threatening consequences; urgent intervention indicated	Death

Eye disorders

	Grade				
**Adverse Event**	**1**	**2**	**3**	**4**	**5**
Blurred vision	Intervention not indicated	Symptomatic; limiting instrumental ADL	Limiting self care ADL	-	-
Definition: A disorder characterized by visual perception of unclear or fuzzy images.					
Cataract	Asymptomatic; clinical or diagnostic observations only; intervention not indicated	Symptomatic; moderate decrease in visual acuity (20/40 or better)	Symptomatic with marked decrease in visual acuity (worse than 20/40 but better than 20/200); operative intervention indicated (e.g., cataract surgery)	Blindness (20/200 or worse) in the affected eye	-
Definition: A disorder characterized by partial or complete opacity of the crystalline lens of one or both eyes. This results in a decrease in visual acuity and eventual blindness if untreated.					
Conjunctivitis	Asymptomatic or mild symptoms; intervention not indicated	Symptomatic; topical intervention indicated (e.g., antibiotics); limiting instrumental ADL	Limiting self care ADL	-	-
Definition: A disorder characterized by inflammation, swelling and redness to the conjunctiva of the eye.					
Corneal ulcer	-	Symptomatic; medical intervention indicated (e.g., topical agents); limiting instrumental ADL	Limiting self care ADL; declining vision (worse than 20/40 but better than 20/200)	Perforation or blindness (20/200 or worse) in the affected eye	-
Definition: A disorder characterized by an area of epithelial tissue loss on the surface of the cornea. It is associated with inflammatory cells in the cornea and anterior chamber.					

Eye disorders

	Grade				
**Adverse Event**	**1**	**2**	**3**	**4**	**5**
Dry eye	Asymptomatic; clinical or diagnostic observations only; mild symptoms relieved by lubricants	Symptomatic; multiple agents indicated; limiting instrumental ADL	Decrease in visual acuity (<20/40); limiting self care ADL	-	-
Definition: A disorder characterized by dryness of the cornea and conjunctiva.					
Extraocular muscle paresis	Asymptomatic; clinical or diagnostic observations only	Symptomatic; limiting instrumental ADL	Limiting self care ADL; disabling	-	-
Definition: A disorder characterized by incomplete paralysis of an extraocular muscle.					
Eye pain	Mild pain	Moderate pain; limiting instrumental ADL	Severe pain; limiting self care ADL	-	-
Definition: A disorder characterized by a sensation of marked discomfort in the eye.					
Eyelid function disorder	Asymptomatic; clinical or diagnostic observations only; intervention not indicated	Symptomatic; nonoperative intervention indicated; limiting instrumental ADL	Limiting self care ADL; operative intervention indicated	-	-
Definition: A disorder characterized by impaired eyelid function.					
Flashing lights	Symptomatic but not limiting ADL	Limiting instrumental ADL	Limiting self care ADL	-	-
Definition: A disorder characterized by a sudden or brief burst of light.					
Floaters	Symptomatic but not limiting ADL	Limiting instrumental ADL	Limiting self care ADL	-	-

Eye disorders

	Grade				
**Adverse Event**	**1**	**2**	**3**	**4**	**5**
Definition: A disorder characterized by an individual seeing spots before their eyes. The spots are shadows of opaque cell fragments in the vitreous humor or lens.					
Glaucoma	Elevated intraocular pressure (EIOP) with single topical agent for intervention; no visual field deficit	EIOP causing early visual field deficits; multiple topical or oral agents indicated; limiting instrumental ADL	EIOP causing marked visual field deficits (e.g., involving both superior and inferior visual fields); operative intervention indicated; limiting self care ADL	Blindness (20/200 or worse) in the affected eye	-
Definition: A disorder characterized by an increase in pressure in the eyeball due to obstruction of the aqueous humor outflow.					
Keratitis	-	Symptomatic; medical intervention indicated (e.g., topical agents); limiting instrumental ADL	Decline in vision (worse than 20/40 but better than 20/200); limiting self care ADL	Perforation or blindness (20/200 or worse) in the affected eye	-
Definition: A disorder characterized by inflammation to the cornea of the eye.					
Night blindness	Symptomatic but not limiting ADL	Limiting instrumental ADL	Limiting self care ADL	Blindness (20/200 or worse) in the affected eye	-
Definition: A disorder characterized by an inability to see clearly in dim light.					
Optic nerve disorder	Asymptomatic; clinical or diagnostic observations only	Limiting vision of the affected eye (20/40 or better)	Limiting vision in the affected eye (worse than 20/40 but better than 20/200)	Blindness (20/200 or worse) in the affected eye	-
Definition: A disorder characterized by involvement of the optic nerve (second cranial nerve).					

Eye disorders

	Grade				
**Adverse Event**	**1**	**2**	**3**	**4**	**5**
Papilledema	Asymptomatic; no visual field defects	Symptomatic decline in vision; visual field defect present sparing the central 20 degrees	Marked visual field defect (worse than 20/40 but better than 20/200)	Blindness (20/200 or worse) in the affected eye	-
Definition: A disorder characterized by swelling around the optic disc.					
Photophobia	Symptomatic but not limiting ADL	Limiting instrumental ADL	Limiting self care ADL	-	-
Definition: A disorder characterized by fear and avoidance of light.					
Retinal detachment	Asymptomatic	Exudative and visual acuity 20/40 or better	Rhegmatogenous or exudative detachment; operative intervention indicated; decline in vision (worse than 20/40 but better than 20/200)	Blindness (20/200 or worse) in the affected eye	-
Definition: A disorder characterized by the separation of the inner retina layers from the underlying pigment epithelium.					
Retinal tear	-	Laser therapy or pneumopexy indicated	Vitroretinal surgical repair indicated	Blindness (20/200 or worse) in the affected eye	-
Definition: A disorder characterized by a small laceration of the retina, this occurs when the vitreous separates from the retina. Symptoms include flashes and floaters.					
Retinal vascular disorder	-	Topical medication indicated	Intravitreal medication; operative intervention indicated	-	-
Definition: A disorder characterized by pathological retinal blood vessels that adversely affects vision.					

Eye disorders

	Grade				
**Adverse Event**	**1**	**2**	**3**	**4**	**5**
Retinopathy	Asymptomatic; clinical or diagnostic observations only	Symptomatic with moderate decrease in visual acuity (20/40 or better); limiting instrumental ADL	Symptomatic with marked decrease in visual acuity (worse than 20/40); disabling; limiting self care ADL	Blindness (20/200 or worse) in the affected eye	-
Definition: A disorder involving the retina.					
Scleral disorder	Asymptomatic; clinical or diagnostic observations only	Symptomatic, limiting instrumental ADL; moderate decrease in visual acuity (20/40 or better)	Symptomatic, limiting self care ADL; marked decrease in visual acuity (worse than 20/40)	Blindness (20/200 or worse) in the affected eye	-
Definition: A disorder characterized by involvement of the sclera of the eye.					
Uveitis	Asymptomatic; clinical or diagnostic observations only	Anterior uveitis; medical intervention indicated	Posterior or pan-uveitis	Blindness (20/200 or worse) in the affected eye	-
Definition: A disorder characterized by inflammation to the uvea of the eye.					
Vitreous hemorrhage	Asymptomatic or mild symptoms; clinical or diagnostic observations only	Symptomatic; limiting instrumental ADL	Limiting self care ADL; vitrectomy indicated	Blindness (20/200 or worse) in the affected eye	-
Definition: A disorder characterized by blood extravasation into the vitreous humor.					
Watering eyes	Intervention not indicated	Intervention indicated	Operative intervention indicated	-	-

Eye disorders					
	Grade				
**Adverse Event**	**1**	**2**	**3**	**4**	**5**
Definition: A disorder of excessive tearing in the eyes; it can be caused by overproduction of tears or impaired drainage of the tear duct.					
Eye disorders - Other, specify	Asymptomatic or mild symptoms; clinical or diagnostic observations only; intervention not indicated	Moderate; minimal, local or noninvasive intervention indicated; limiting age-appropriate instrumental ADL	Severe or medically significant but not immediately sight-threatening; hospitalization or prolongation of existing hospitalization indicated; disabling; limiting self care ADL	Sight-threatening consequences; urgent intervention indicated; blindness (20/200 or worse) in the affected eye	-

Gastrointestinal disorders

	Grade				
**Adverse Event**	**1**	**2**	**3**	**4**	**5**
Abdominal distension	Asymptomatic; clinical or diagnostic observations only; intervention not indicated	Symptomatic; limiting instrumental ADL	Severe discomfort; limiting self care ADL	-	-
Definition: A disorder characterized by swelling of the abdomen.					
Abdominal pain	Mild pain	Moderate pain; limiting instrumental ADL	Severe pain; limiting self care ADL	-	-
Definition: A disorder characterized by a sensation of marked discomfort in the abdominal region.					
Anal fistula	Asymptomatic; clinical or diagnostic observations only; intervention not indicated	Symptomatic; altered GI function	Severely altered GI function; tube feeding, TPN or hospitalization indicated; elective operative intervention indicated	Life-threatening consequences; urgent intervention indicated	Death
Definition: A disorder characterized by an abnormal communication between the opening in the anal canal to the perianal skin.					
Anal hemorrhage	Mild; intervention not indicated	Moderate symptoms; medical intervention or minor cauterization indicated	Transfusion, radiologic, endoscopic, or elective operative intervention indicated	Life-threatening consequences; urgent intervention indicated	Death
Definition: A disorder characterized by bleeding from the anal region.					
Anal mucositis	Asymptomatic or mild symptoms; intervention not indicated	Symptomatic; medical intervention indicated; limiting instrumental ADL	Severe symptoms; limiting self care ADL	Life-threatening consequences; urgent intervention indicated	Death
Definition: A disorder characterized by inflammation of the mucous membrane of the anus.					

Gastrointestinal disorders

	Grade				
**Adverse Event**	**1**	**2**	**3**	**4**	**5**
Anal necrosis	-	-	TPN or hospitalization indicated; radiologic, endoscopic, or operative intervention indicated	Life-threatening consequences; urgent operative intervention indicated	Death
Definition: A disorder characterized by a necrotic process occurring in the anal region.					
Anal pain	Mild pain	Moderate pain; limiting instrumental ADL	Severe pain; limiting self care ADL	-	-
Definition: A disorder characterized by a sensation of marked discomfort in the anal region.					
Anal stenosis	Asymptomatic; clinical or diagnostic observations only; intervention not indicated	Symptomatic; altered GI function	Symptomatic and severely altered GI function; non-emergent operative intervention indicated; TPN or hospitalization indicated	Life-threatening consequences; urgent operative intervention indicated	Death
Definition: A disorder characterized by a narrowing of the lumen of the anal canal.					
Anal ulcer	Asymptomatic; clinical or diagnostic observations only; intervention not indicated	Symptomatic; altered GI function	Severely altered GI function; TPN indicated; elective operative or endoscopic intervention indicated; disabling	Life-threatening consequences; urgent operative intervention indicated	Death
Definition: A disorder characterized by a circumscribed, inflammatory and necrotic erosive lesion on the mucosal surface of the anal canal.					

Gastrointestinal disorders

	Grade				
**Adverse Event**	**1**	**2**	**3**	**4**	**5**
Ascites	Asymptomatic; clinical or diagnostic observations only; intervention not indicated	Symptomatic; medical intervention indicated	Severe symptoms; invasive intervention indicated	Life-threatening consequences; urgent operative intervention indicated	Death
Definition: A disorder characterized by accumulation of serous or hemorrhagic fluid in the peritoneal cavity.					
Bloating	No change in bowel function or oral intake	Symptomatic, decreased oral intake; change in bowel function	-	-	-
Definition: A disorder characterized by subject-reported feeling of uncomfortable fullness of the abdomen.					
Cecal hemorrhage	Mild; intervention not indicated	Moderate symptoms; medical intervention or minor cauterization indicated	Transfusion, radiologic, endoscopic, or elective operative intervention indicated	Life-threatening consequences; urgent intervention indicated	Death
Definition: A disorder characterized by bleeding from the cecum.					
Cheilitis	Asymptomatic; clinical or diagnostic observations only; intervention not indicated	Moderate symptoms; limiting instrumental ADL	Severe symptoms; limiting self care ADL; intervention indicated	-	-
Definition: A disorder characterized by inflammation of the lip.					
Colitis	Asymptomatic; clinical or diagnostic observations only; intervention not indicated	Abdominal pain; mucus or blood in stool	Severe abdominal pain; change in bowel habits; medical intervention indicated; peritoneal signs	Life-threatening consequences; urgent intervention indicated	Death
Definition: A disorder characterized by inflammation of the colon.					

Gastrointestinal disorders

	Grade				
**Adverse Event**	**1**	**2**	**3**	**4**	**5**
Colonic fistula	Asymptomatic; clinical or diagnostic observations only; intervention not indicated	Symptomatic; altered GI function	Severely altered GI function; bowel rest, TPN or hospitalization indicated; elective operative intervention indicated	Life-threatening consequences; urgent intervention indicated	Death
Definition: A disorder characterized by an abnormal communication between the large intestine and another organ or anatomic site.					
Colonic hemorrhage	Mild; intervention not indicated	Moderate symptoms; medical intervention or minor cauterization indicated	Transfusion, radiologic, endoscopic, or elective operative intervention indicated	Life-threatening consequences; urgent intervention indicated	Death
Definition: A disorder characterized by bleeding from the colon.					
Colonic obstruction	Asymptomatic; clinical or diagnostic observations only; intervention not indicated	Symptomatic; altered GI function	Hospitalization indicated; elective operative intervention indicated; disabling	Life-threatening consequences; urgent operative intervention indicated	Death
Definition: A disorder characterized by blockage of the normal flow of the intestinal contents in the colon.					
Colonic perforation	-	Symptomatic; medical intervention indicated	Severe symptoms; elective operative intervention indicated	Life-threatening consequences; urgent intervention indicated	Death
Definition: A disorder characterized by a rupture in the colonic wall.					

Gastrointestinal disorders

	Grade				
**Adverse Event**	**1**	**2**	**3**	**4**	**5**
Colonic stenosis	Asymptomatic; clinical or diagnostic observations only; intervention not indicated	Symptomatic; altered GI function	Severely altered GI function; tube feeding or hospitalization indicated; elective operative intervention indicated	Life-threatening consequences; urgent operative intervention indicated	Death
Definition: A disorder characterized by a narrowing of the lumen of the colon.					
Colonic ulcer	Asymptomatic; clinical or diagnostic observations only; intervention not indicated	Symptomatic; altered GI function	Severely altered GI function; TPN indicated; elective operative or endoscopic intervention indicated; disabling	Life-threatening consequences; urgent operative intervention indicated	Death
Definition: A disorder characterized by a circumscribed, inflammatory and necrotic erosive lesion on the mucosal surface of the colon.					
Constipation	Occasional or intermittent symptoms; occasional use of stool softeners, laxatives, dietary modification, or enema	Persistent symptoms with regular use of laxatives or enemas; limiting instrumental ADL	Obstipation with manual evacuation indicated; limiting self care ADL	Life-threatening consequences; urgent intervention indicated	Death
Definition: A disorder characterized by irregular and infrequent or difficult evacuation of the bowels.					
Dental caries	One or more dental caries, not involving the root	Dental caries involving the root	Dental caries resulting in pulpitis or periapical abscess or resulting in tooth loss	-	-
Definition: A disorder characterized by the decay of a tooth, in which it becomes softened, discolored and/or porous.					

Gastrointestinal disorders

	Grade				
**Adverse Event**	**1**	**2**	**3**	**4**	**5**
Diarrhea	Increase of <4 stools per day over baseline; mild increase in ostomy output compared to baseline	Increase of 4 - 6 stools per day over baseline; moderate increase in ostomy output compared to baseline	Increase of >=7 stools per day over baseline; incontinence; hospitalization indicated; severe increase in ostomy output compared to baseline; limiting self care ADL	Life-threatening consequences; urgent intervention indicated	Death
Definition: A disorder characterized by frequent and watery bowel movements.					
Dry mouth	Symptomatic (e.g., dry or thick saliva) without significant dietary alteration; unstimulated saliva flow >0.2 ml/min	Moderate symptoms; oral intake alterations (e.g., copious water, other lubricants, diet limited to purees and/or soft, moist foods); unstimulated saliva 0.1 to 0.2 ml/min	Inability to adequately aliment orally; tube feeding or TPN indicated; unstimulated saliva <0.1 ml/min	-	-
Definition: A disorder characterized by reduced salivary flow in the oral cavity.					
Duodenal fistula	Asymptomatic; clinical or diagnostic observations only; intervention not indicated	Symptomatic; altered GI function	Severely altered GI function; tube feeding, TPN or hospitalization indicated; elective operative intervention indicated	Life-threatening consequences; urgent intervention indicated	Death
Definition: A disorder characterized by an abnormal communication between the duodenum and another organ or anatomic site.					

Gastrointestinal disorders

	Grade				
**Adverse Event**	**1**	**2**	**3**	**4**	**5**
Duodenal hemorrhage	Mild; intervention not indicated	Moderate symptoms; medical intervention or minor cauterization indicated	Transfusion, radiologic, endoscopic, or elective operative intervention indicated	Life-threatening consequences; urgent intervention indicated	Death
Definition: A disorder characterized by bleeding from the duodenum.					
Duodenal obstruction	Asymptomatic; clinical or diagnostic observations only; intervention not indicated	Symptomatic; altered GI function	Hospitalization or elective operative intervention indicated; disabling	Life-threatening consequences; urgent operative intervention indicated	Death
Definition: A disorder characterized by blockage of the normal flow of stomach contents through the duodenum.					
Duodenal perforation	-	Symptomatic; medical intervention indicated	Severe symptoms; elective operative intervention indicated	Life-threatening consequences; urgent operative intervention indicated	Death
Definition: A disorder characterized by a rupture in the duodenal wall.					
Duodenal stenosis	Asymptomatic; clinical or diagnostic observations only; intervention not indicated	Symptomatic; altered GI function	Severely altered GI function; tube feeding; hospitalization indicated; elective operative intervention indicated	Life-threatening consequences; urgent operative intervention indicated	Death
Definition: A disorder characterized by a narrowing of the lumen of the duodenum.					

Gastrointestinal disorders					
	Grade				
**Adverse Event**	**1**	**2**	**3**	**4**	**5**
Duodenal ulcer	Asymptomatic; clinical or diagnostic observations only; intervention not indicated	Moderate symptoms; medical intervention indicated; limiting instrumental ADL	Severely altered GI function; TPN indicated; elective operative or endoscopic intervention indicated; limiting self care ADL; disabling	Life-threatening consequences; urgent operative intervention indicated	Death
Definition: A disorder characterized by a circumscribed, inflammatory and necrotic erosive lesion on the mucosal surface of the duodenal wall.					
Dyspepsia	Mild symptoms; intervention not indicated	Moderate symptoms; medical intervention indicated	Severe symptoms; surgical intervention indicated	-	-
Definition: A disorder characterized by an uncomfortable, often painful feeling in the stomach, resulting from impaired digestion. Symptoms include burning stomach, bloating, heartburn, nausea and vomiting.					
Dysphagia	Symptomatic, able to eat regular diet	Symptomatic and altered eating/swallowing	Severely altered eating/swallowing; tube feeding or TPN or hospitalization indicated	Life-threatening consequences; urgent intervention indicated	Death
Definition: A disorder characterized by difficulty in swallowing.					
Enterocolitis	Asymptomatic; clinical or diagnostic observations only; intervention not indicated	Abdominal pain; mucus or blood in stool	Severe or persistent abdominal pain; fever; ileus; peritoneal signs	Life-threatening consequences; urgent intervention indicated	Death
Definition: A disorder characterized by inflammation of the small and large intestines.					
Enterovesical fistula	Asymptomatic; clinical or diagnostic observations only; intervention not indicated	Symptomatic; noninvasive intervention indicated	Severe, medically significant; medical intervention indicated	Life-threatening consequences; urgent intervention indicated	Death
Definition: A disorder characterized by an abnormal communication between the urinary bladder and the intestine.					

Gastrointestinal disorders

	Grade				
**Adverse Event**	**1**	**2**	**3**	**4**	**5**
Esophageal fistula	Asymptomatic; clinical or diagnostic observations only; intervention not indicated	Symptomatic; altered GI function	Severely altered GI function; tube feeding, TPN or hospitalization indicated; elective operative intervention indicated	Life-threatening consequences; urgent intervention indicated	Death
Definition: A disorder characterized by an abnormal communication between the esophagus and another organ or anatomic site.					
Esophageal hemorrhage	Mild; intervention not indicated	Moderate symptoms; medical intervention or minor cauterization indicated	Transfusion, radiologic, endoscopic, or elective operative intervention indicated	Life-threatening consequences; urgent intervention indicated	Death
Definition: A disorder characterized by bleeding from the esophagus.					
Esophageal necrosis	-	-	Inability to aliment adequately by GI tract; radiologic, endoscopic, or operative intervention indicated	Life-threatening consequences; urgent operative intervention indicated	Death
Definition: A disorder characterized by a necrotic process occurring in the esophageal wall.					
Esophageal obstruction	Asymptomatic; clinical or diagnostic observations only; intervention not indicated	Symptomatic; altered GI function; limiting instrumental ADL	Hospitalization indicated; elective operative intervention indicated; limiting self care ADL; disabling	Life-threatening consequences; urgent intervention indicated	Death
Definition: A disorder characterized by blockage of the normal flow of the contents in the esophagus.					
Esophageal pain	Mild pain	Moderate pain; limiting instrumental ADL	Severe pain; limiting self care ADL	-	-

Gastrointestinal disorders					
	Grade				
**Adverse Event**	**1**	**2**	**3**	**4**	**5**
Definition: A disorder characterized by a sensation of marked discomfort in the esophageal region.					
Esophageal perforation	-	Symptomatic; medical intervention indicated	Severe symptoms; elective operative intervention indicated	Life-threatening consequences; urgent operative intervention indicated	Death
Definition: A disorder characterized by a rupture in the wall of the esophagus.					
Esophageal stenosis	Asymptomatic; clinical or diagnostic observations only; intervention not indicated	Symptomatic; altered GI function	Severely altered GI function; tube feeding; hospitalization indicated; elective operative intervention indicated	Life-threatening consequences; urgent operative intervention indicated	Death
Definition: A disorder characterized by a narrowing of the lumen of the esophagus.					
Esophageal ulcer	Asymptomatic; clinical or diagnostic observations only; intervention not indicated	Symptomatic; altered GI function; limiting instrumental ADL	Severely altered GI function; TPN indicated; elective operative or endoscopic intervention indicated; limiting self care ADL; disabling	Life-threatening consequences; urgent operative intervention indicated	Death
Definition: A disorder characterized by a circumscribed, inflammatory and necrotic erosive lesion on the mucosal surface of the esophageal wall.					
Esophageal varices hemorrhage	-	Self-limited; intervention not indicated	Transfusion, radiologic, endoscopic, or elective operative intervention indicated	Life-threatening consequences; urgent intervention indicated	Death
Definition: A disorder characterized by bleeding from esophageal varices.					

Gastrointestinal disorders					
	Grade				
**Adverse Event**	**1**	**2**	**3**	**4**	**5**
Esophagitis	Asymptomatic; clinical or diagnostic observations only; intervention not indicated	Symptomatic; altered eating/swallowing; oral supplements indicated	Severely altered eating/swallowing; tube feeding, TPN or hospitalization indicated	Life-threatening consequences; urgent operative intervention indicated	Death
Definition: A disorder characterized by inflammation of the esophageal wall.					
Fecal incontinence	Occasional use of pads required	Daily use of pads required	Severe symptoms; elective operative intervention indicated	-	-
Definition: A disorder characterized by inability to control the escape of stool from the rectum.					
Flatulence	Mild symptoms; intervention not indicated	Moderate; persistent; psychosocial sequelae	-	-	-
Definition: A disorder characterized by a state of excessive gas in the alimentary canal.					
Gastric fistula	Asymptomatic; clinical or diagnostic observations only; intervention not indicated	Symptomatic; altered GI function	Severely altered GI function; bowel rest; tube feeding, TPN or hospitalization indicated; elective operative intervention indicated	Life-threatening consequences; urgent operative intervention indicated	Death
Definition: A disorder characterized by an abnormal communication between the stomach and another organ or anatomic site.					
Gastric hemorrhage	Mild; intervention not indicated	Moderate symptoms; medical intervention or minor cauterization indicated	Transfusion, radiologic, endoscopic, or elective operative intervention indicated	Life-threatening consequences; urgent intervention indicated	Death
Definition: A disorder characterized by bleeding from the gastric wall.					

Gastrointestinal disorders

	Grade				
**Adverse Event**	**1**	**2**	**3**	**4**	**5**
Gastric necrosis	-	-	Inability to aliment adequately by GI tract; radiologic, endoscopic, or operative intervention indicated	Life-threatening consequences; urgent operative intervention indicated	Death
Definition: A disorder characterized by a necrotic process occurring in the gastric wall.					
Gastric perforation	-	Symptomatic; medical intervention indicated	Severe symptoms; elective operative intervention indicated	Life-threatening consequences; urgent operative intervention indicated	Death
Definition: A disorder characterized by a rupture in the stomach wall.					
Gastric stenosis	Asymptomatic; clinical or diagnostic observations only; intervention not indicated	Symptomatic; altered GI function	Severely altered GI function; tube feeding; hospitalization indicated; elective operative intervention indicated	Life-threatening consequences; urgent operative intervention indicated	Death
Definition: A disorder characterized by a narrowing of the lumen of the stomach.					
Gastric ulcer	Asymptomatic; clinical or diagnostic observations only; intervention not indicated	Symptomatic; altered GI function; medical intervention indicated; limiting instrumental ADL	Severely altered GI function; TPN indicated; elective operative or endoscopic intervention indicated; limiting self care ADL; disabling	Life-threatening consequences; urgent operative intervention indicated	Death
Definition: A disorder characterized by a circumscribed, inflammatory and necrotic erosive lesion on the mucosal surface of the stomach.					

Gastrointestinal disorders					
	Grade				
**Adverse Event**	**1**	**2**	**3**	**4**	**5**
Gastritis	Asymptomatic; clinical or diagnostic observations only; intervention not indicated	Symptomatic; altered GI function; medical intervention indicated	Severely altered eating or gastric function; TPN or hospitalization indicated	Life-threatening consequences; urgent operative intervention indicated	Death
Definition: A disorder characterized by inflammation of the stomach.					
Gastroesophageal reflux disease	Mild symptoms; intervention not indicated	Moderate symptoms; medical intervention indicated	Severe symptoms; surgical intervention indicated	-	-
Definition: A disorder characterized by reflux of the gastric and/or duodenal contents into the distal esophagus. It is chronic in nature and usually caused by incompetence of the lower esophageal sphincter, and may result in injury to the esophageal mucosal. Symptoms include heartburn and acid indigestion.					
Gastrointestinal fistula	Asymptomatic; clinical or diagnostic observations only; intervention not indicated	Symptomatic; altered GI function	Severely altered GI function; tube feeding, TPN or hospitalization indicated	Life-threatening consequences; urgent operative intervention indicated	Death
Definition: A disorder characterized by an abnormal communication between any part of the gastrointestinal system and another organ or anatomic site.					
Gastrointestinal pain	Mild pain	Moderate pain; limiting instrumental ADL	Severe pain; limiting self care ADL	-	-
Definition: A disorder characterized by a sensation of marked discomfort in the gastrointestinal region.					
Gastroparesis	Mild nausea, early satiety and bloating, able to maintain caloric intake on regular diet	Moderate symptoms; able to maintain nutrition with dietary and lifestyle modifications; may need pharmacologic intervention	Weight loss; refractory to medical intervention; unable to maintain nutrition orally	-	-

Gastrointestinal disorders					
	Grade				
**Adverse Event**	**1**	**2**	**3**	**4**	**5**
Definition: A disorder characterized by an incomplete paralysis of the muscles of the stomach wall resulting in delayed emptying of the gastric contents into the small intestine.					
Gingival pain	Mild pain	Moderate pain interfering with oral intake	Severe pain; inability to aliment orally	-	-
Definition: A disorder characterized by a sensation of marked discomfort in the gingival region.					
Hemorrhoidal hemorrhage	Mild; intervention not indicated	Moderate symptoms; medical intervention or minor cauterization indicated	Transfusion, radiologic, endoscopic, or elective operative intervention indicated	Life-threatening consequences; urgent intervention indicated	Death
Definition: A disorder characterized by bleeding from the hemorrhoids.					
Hemorrhoids	Asymptomatic; clinical or diagnostic observations only; intervention not indicated	Symptomatic; banding or medical intervention indicated	Severe symptoms; radiologic, endoscopic or elective operative intervention indicated	-	-
Definition: A disorder characterized by the presence of dilated veins in the rectum and surrounding area.					
Ileal fistula	Asymptomatic; clinical or diagnostic observations only; intervention not indicated	Symptomatic; altered GI function	Severely altered GI function; TPN or hospitalization indicated; elective operative intervention indicated	Life-threatening consequences; urgent intervention indicated	Death
Definition: A disorder characterized by an abnormal communication between the ileum and another organ or anatomic site.					

Gastrointestinal disorders

	Grade				
**Adverse Event**	**1**	**2**	**3**	**4**	**5**
Ileal hemorrhage	Mild; intervention not indicated	Moderate symptoms; medical intervention or minor cauterization indicated	Transfusion, radiologic, endoscopic, or elective operative intervention indicated	Life-threatening consequences; urgent intervention indicated	Death
Definition: A disorder characterized by bleeding from the ileal wall.					
Ileal obstruction	Asymptomatic; clinical or diagnostic observations only; intervention not indicated	Symptomatic; altered GI function; limiting instrumental ADL	Hospitalization indicated; elective operative intervention indicated; limiting self care ADL; disabling	Life-threatening consequences; urgent operative intervention indicated	Death
Definition: A disorder characterized by blockage of the normal flow of the intestinal contents in the ileum.					
Ileal perforation	-	Symptomatic; medical intervention indicated	Severe symptoms; elective operative intervention indicated	Life-threatening consequences; urgent operative intervention indicated	Death
Definition: A disorder characterized by a rupture in the ileal wall.					
Ileal stenosis	Asymptomatic; clinical or diagnostic observations only; intervention not indicated	Symptomatic; altered GI function	Severely altered GI function; tube feeding or hospitalization indicated; elective operative intervention indicated	Life-threatening consequences; urgent operative intervention indicated	Death
Definition: A disorder characterized by a narrowing of the lumen of the ileum.					

Gastrointestinal disorders					
	Grade				
**Adverse Event**	**1**	**2**	**3**	**4**	**5**
Ileal ulcer	Asymptomatic; clinical or diagnostic observations only; intervention not indicated	Symptomatic; altered GI function	Severely altered GI function; TPN indicated; elective operative or endoscopic intervention indicated; disabling	Life-threatening consequences; urgent operative intervention indicated	Death
Definition: A disorder characterized by a circumscribed, inflammatory and necrotic erosive lesion on the mucosal surface of the ileum.					
Ileus	-	Symptomatic; altered GI function; bowel rest indicated	Severely altered GI function; TPN indicated	Life-threatening consequences; urgent intervention indicated	Death
Definition: A disorder characterized by failure of the ileum to transport intestinal contents.					
Intra-abdominal hemorrhage	-	Medical intervention or minor cauterization indicated	Transfusion, radiologic, endoscopic, or elective operative intervention indicated	Life-threatening consequences; urgent intervention indicated	Death
Definition: A disorder characterized by bleeding in the abdominal cavity.					
Jejunal fistula	Asymptomatic; clinical or diagnostic observations only; intervention not indicated	Symptomatic; altered GI function	Severely altered GI function; TPN or hospitalization indicated; elective operative intervention indicated	Life-threatening consequences; urgent intervention indicated	Death
Definition: A disorder characterized by an abnormal communication between the jejunum and another organ or anatomic site.					

Gastrointestinal disorders

	Grade				
**Adverse Event**	**1**	**2**	**3**	**4**	**5**
Jejunal hemorrhage	Mild; intervention not indicated	Moderate symptoms; medical intervention or minor cauterization indicated	Transfusion, radiologic, endoscopic, or elective operative intervention indicated	Life-threatening consequences; urgent intervention indicated	Death
Definition: A disorder characterized by bleeding from the jejunal wall.					
Jejunal obstruction	Asymptomatic; clinical or diagnostic observations only; intervention not indicated	Symptomatic; altered GI function; limiting instrumental ADL	Hospitalization indicated; elective operative intervention indicated; limiting self care ADL; disabling	Life-threatening consequences; urgent operative intervention indicated	Death
Definition: A disorder characterized by blockage of the normal flow of the intestinal contents in the jejunum.					
Jejunal perforation	-	Symptomatic; medical intervention indicated	Severe symptoms; elective operative intervention indicated	Life-threatening consequences; urgent operative intervention indicated	Death
Definition: A disorder characterized by a rupture in the jejunal wall.					
Jejunal stenosis	Asymptomatic; clinical or diagnostic observations only; intervention not indicated	Symptomatic; altered GI function	Severely altered GI function; tube feeding or hospitalization indicated; elective operative intervention indicated	Life-threatening consequences; urgent operative intervention indicated	Death
Definition: A disorder characterized by a narrowing of the lumen of the jejunum.					

Gastrointestinal disorders

	Grade				
**Adverse Event**	**1**	**2**	**3**	**4**	**5**
Jejunal ulcer	Asymptomatic; clinical or diagnostic observations only; intervention not indicated	Symptomatic; altered GI function	Severely altered GI function; TPN indicated; elective operative or endoscopic intervention indicated; disabling	Life-threatening consequences; urgent operative intervention indicated	Death
Definition: A disorder characterized by a circumscribed, inflammatory and necrotic erosive lesion on the mucosal surface of the jejunum.					
Lip pain	Mild pain	Moderate pain; limiting instrumental ADL	Severe pain; limiting self care ADL	-	-
Definition: A disorder characterized by a sensation of marked discomfort of the lip.					
Lower gastrointestinal hemorrhage	Mild; intervention not indicated	Moderate symptoms; medical intervention or minor cauterization indicated	Transfusion, radiologic, endoscopic, or elective operative intervention indicated	Life-threatening consequences; urgent intervention indicated	Death
Definition: A disorder characterized by bleeding from the lower gastrointestinal tract (small intestine, large intestine, and anus).					
Malabsorption	-	Altered diet; oral intervention indicated	Inability to aliment adequately; TPN indicated	Life-threatening consequences; urgent intervention indicated	Death
Definition: A disorder characterized by inadequate absorption of nutrients in the small intestine. Symptoms include abdominal marked discomfort, bloating and diarrhea.					
Mucositis oral	Asymptomatic or mild symptoms; intervention not indicated	Moderate pain; not interfering with oral intake; modified diet indicated	Severe pain; interfering with oral intake	Life-threatening consequences; urgent intervention indicated	Death
Definition: A disorder characterized by inflammation of the oral mucosal.					

Gastrointestinal disorders

	Grade				
**Adverse Event**	**1**	**2**	**3**	**4**	**5**
Nausea	Loss of appetite without alteration in eating habits	Oral intake decreased without significant weight loss, dehydration or malnutrition	Inadequate oral caloric or fluid intake; tube feeding, TPN, or hospitalization indicated	-	-
Definition: A disorder characterized by a queasy sensation and/or the urge to vomit.					
Obstruction gastric	Asymptomatic; clinical or diagnostic observations only; intervention not indicated	Symptomatic; altered GI function; limiting instrumental ADL	Hospitalization indicated; elective operative intervention indicated; limiting self care ADL; disabling	Life-threatening consequences; urgent operative intervention indicated	Death
Definition: A disorder characterized by blockage of the normal flow of the contents in the stomach.					
Oral cavity fistula	Asymptomatic; clinical or diagnostic observations only; intervention not indicated	Symptomatic; altered GI function	Severely altered GI function; TPN or hospitalization indicated; elective operative intervention indicated	Life-threatening consequences; urgent intervention indicated	Death
Definition: A disorder characterized by an abnormal communication between the oral cavity and another organ or anatomic site.					
Oral dysesthesia	Mild discomfort; not interfering with oral intake	Moderate pain; interfering with oral intake	Disabling pain; tube feeding or TPN indicated	-	-
Definition: A disorder characterized by a burning or tingling sensation on the lips, tongue or entire mouth.					
Oral hemorrhage	Mild; intervention not indicated	Moderate symptoms; medical intervention or minor cauterization indicated	Transfusion, radiologic, endoscopic, or elective operative intervention indicated	Life-threatening consequences; urgent intervention indicated	Death
Definition: A disorder characterized by bleeding from the mouth.					

Gastrointestinal disorders

	Grade				
**Adverse Event**	**1**	**2**	**3**	**4**	**5**
Oral pain	Mild pain	Moderate pain; limiting instrumental ADL	Severe pain; limiting self care ADL	-	-
Definition: A disorder characterized by a sensation of marked discomfort in the mouth, tongue or lips.					
Pancreatic duct stenosis	Asymptomatic; clinical or diagnostic observations only; intervention not indicated	Symptomatic; altered GI function	Severely altered GI function; tube feeding or hospitalization indicated; elective operative intervention indicated	Life-threatening consequences; urgent operative intervention indicated	Death
Definition: A disorder characterized by a narrowing of the lumen of the pancreatic duct.					
Pancreatic fistula	Asymptomatic; clinical or diagnostic observations only; intervention not indicated	Symptomatic; altered GI function	Severely altered GI function; tube feeding or TPN or hospitalization indicated; elective operative intervention indicated	Life-threatening consequences; urgent operative intervention indicated	Death
Definition: A disorder characterized by an abnormal communication between the pancreas and another organ or anatomic site.					
Pancreatic hemorrhage	Mild; intervention not indicated	Moderate symptoms; medical intervention or minor cauterization indicated	Transfusion, radiologic, endoscopic, or elective operative intervention indicated	Life-threatening consequences; urgent intervention indicated	Death
Definition: A disorder characterized by bleeding from the pancreas.					
Pancreatic necrosis	-	-	Tube feeding or TPN indicated; radiologic, endoscopic, or operative intervention indicated	Life-threatening consequences; urgent operative intervention indicated	Death

Gastrointestinal disorders

	Grade				
**Adverse Event**	**1**	**2**	**3**	**4**	**5**
Definition: A disorder characterized by a necrotic process occurring in the pancreas.					
Pancreatitis	-	Enzyme elevation or radiologic findings only	Severe pain; vomiting; medical intervention indicated (e.g., analgesia, nutritional support)	Life-threatening consequences; urgent intervention indicated	Death
Definition: A disorder characterized by inflammation of the pancreas.					
Periodontal disease	Gingival recession or gingivitis; limited bleeding on probing; mild local bone loss	Moderate gingival recession or gingivitis; multiple sites of bleeding on probing; moderate bone loss	Spontaneous bleeding; severe bone loss with or without tooth loss; osteonecrosis of maxilla or mandible	-	-
Definition: A disorder in the gingival tissue around the teeth.					
Peritoneal necrosis	-	-	Tube feeding or TPN indicated; radiologic, endoscopic, or operative intervention indicated	Life-threatening consequences; urgent operative intervention indicated	Death
Definition: A disorder characterized by a necrotic process occurring in the peritoneum.					
Proctitis	Rectal discomfort, intervention not indicated	Symptoms (e.g., rectal discomfort, passing blood or mucus); medical intervention indicated; limiting instrumental ADL	Severe symptoms; fecal urgency or stool incontinence; limiting self care ADL	Life-threatening consequences; urgent intervention indicated	Death
Definition: A disorder characterized by inflammation of the rectum.					

Gastrointestinal disorders

	Grade				
**Adverse Event**	**1**	**2**	**3**	**4**	**5**
Rectal fistula	Asymptomatic; clinical or diagnostic observations only; intervention not indicated	Symptomatic; altered GI function	Severely altered GI function; TPN or hospitalization indicated; elective operative intervention indicated	Life-threatening consequences; urgent intervention indicated	Death
Definition: A disorder characterized by an abnormal communication between the rectum and another organ or anatomic site.					
Rectal hemorrhage	Mild; intervention not indicated	Moderate symptoms; medical intervention or minor cauterization indicated	Transfusion, radiologic, endoscopic, or elective operative intervention indicated	Life-threatening consequences; urgent intervention indicated	Death
Definition: A disorder characterized by bleeding from the rectal wall and discharged from the anus.					
Rectal mucositis	Asymptomatic or mild symptoms; intervention not indicated	Symptomatic; medical intervention indicated; limiting instrumental ADL	Severe symptoms; limiting self care ADL	Life-threatening consequences; urgent operative intervention indicated	Death
Definition: A disorder characterized by inflammation of the mucous membrane of the rectum.					
Rectal necrosis	-	-	Tube feeding or TPN indicated; radiologic, endoscopic, or operative intervention indicated	Life-threatening consequences; urgent operative intervention indicated	Death
Definition: A disorder characterized by a necrotic process occurring in the rectal wall.					

Gastrointestinal disorders

	Grade				
**Adverse Event**	**1**	**2**	**3**	**4**	**5**
Rectal obstruction	Asymptomatic; clinical or diagnostic observations only; intervention not indicated	Symptomatic; altered GI function; limiting instrumental ADL	Hospitalization indicated; elective operative intervention indicated; limiting self care ADL; disabling	Life-threatening consequences; urgent operative intervention indicated	Death
Definition: A disorder characterized by blockage of the normal flow of the intestinal contents in the rectum.					
Rectal pain	Mild pain	Moderate pain; limiting instrumental ADL	Severe pain; limiting self care ADL	-	-
Definition: A disorder characterized by a sensation of marked discomfort in the rectal region.					
Rectal perforation	-	Symptomatic; medical intervention indicated	Severe symptoms; elective operative intervention indicated	Life-threatening consequences; urgent operative intervention indicated	Death
Definition: A disorder characterized by a rupture in the rectal wall.					
Rectal stenosis	Asymptomatic; clinical or diagnostic observations only; intervention not indicated	Symptomatic; altered GI function	Severely altered GI function; tube feeding or hospitalization indicated; elective operative intervention indicated	Life-threatening consequences; urgent operative intervention indicated	Death
Definition: A disorder characterized by a narrowing of the lumen of the rectum.					
Rectal ulcer	Asymptomatic; clinical or diagnostic observations only; intervention not indicated	Symptomatic; altered GI function (e.g. altered dietary habits, vomiting, diarrhea)	Severely altered GI function; TPN indicated; elective operative or endoscopic intervention indicated; disabling	Life-threatening consequences; urgent operative intervention indicated	Death

Gastrointestinal disorders					
	Grade				
**Adverse Event**	**1**	**2**	**3**	**4**	**5**
Definition: A disorder characterized by a circumscribed, inflammatory and necrotic erosive lesion on the mucosal surface of the rectum.					
Retroperitoneal hemorrhage	-	Self-limited; intervention indicated	Transfusion, medical, radiologic, endoscopic, or elective operative intervention indicated	Life-threatening consequences; urgent intervention indicated	Death
Definition: A disorder characterized by bleeding from the retroperitoneal area.					
Salivary duct inflammation	Slightly thickened saliva; slightly altered taste (e.g., metallic)	Thick, ropy, sticky saliva; markedly altered taste; alteration in diet indicated; secretion-induced symptoms; limiting instrumental ADL	Acute salivary gland necrosis; severe secretion-induced symptoms (e.g., thick saliva/oral secretions or gagging); tube feeding or TPN indicated; limiting self care ADL; disabling	Life-threatening consequences; urgent intervention indicated	Death
Definition: A disorder characterized by inflammation of the salivary duct.					
Salivary gland fistula	Asymptomatic; clinical or diagnostic observations only; intervention not indicated	Symptomatic; altered GI function; tube feeding indicated	Severely altered GI function; hospitalization indicated; elective operative intervention indicated	Life-threatening consequences; urgent operative intervention indicated	Death
Definition: A disorder characterized by an abnormal communication between a salivary gland and another organ or anatomic site.					
Small intestinal mucositis	Asymptomatic or mild symptoms; intervention not indicated	Symptomatic; medical intervention indicated; limiting instrumental ADL	Severe pain; interfering with oral intake; tube feeding, TPN or hospitalization indicated; limiting self care ADL	Life-threatening consequences; urgent intervention indicated	Death

Gastrointestinal disorders					
	Grade				
**Adverse Event**	**1**	**2**	**3**	**4**	**5**
Definition: A disorder characterized by inflammation of the mucous membrane of the small intestine.					
Small intestinal obstruction	Asymptomatic; clinical or diagnostic observations only; intervention not indicated	Symptomatic; altered GI function; limiting instrumental ADL	Hospitalization indicated; elective operative intervention indicated; limiting self care ADL; disabling	Life-threatening consequences; urgent operative intervention indicated	Death
Definition: A disorder characterized by blockage of the normal flow of the intestinal contents.					
Small intestinal perforation	-	Symptomatic; medical intervention indicated	Severe symptoms; elective operative intervention indicated	Life-threatening consequences; urgent operative intervention indicated	Death
Definition: A disorder characterized by a rupture in the small intestine wall.					
Small intestinal stenosis	Asymptomatic; clinical or diagnostic observations only; intervention not indicated	Symptomatic; altered GI function	Symptomatic and severely altered GI function; tube feeding, TPN or hospitalization indicated; non-emergent operative intervention indicated	Life-threatening consequences; urgent operative intervention indicated	Death
Definition: A disorder characterized by a narrowing of the lumen of the small intestine.					
Small intestine ulcer	Asymptomatic; clinical or diagnostic observations only; intervention not indicated	Symptomatic; altered GI function; limiting instrumental ADL	Severely altered GI function; TPN indicated; elective operative or endoscopic intervention indicated; limiting self care ADL; disabling	Life-threatening consequences; urgent operative intervention indicated	Death

Gastrointestinal disorders					
	Grade				
**Adverse Event**	**1**	**2**	**3**	**4**	**5**
Definition: A disorder characterized by a circumscribed, inflammatory and necrotic erosive lesion on the mucosal surface of the small intestine.					
Stomach pain	Mild pain	Moderate pain; limiting instrumental ADL	Severe pain; limiting self care ADL	-	-
Definition: A disorder characterized by a sensation of marked discomfort in the stomach.					
Tooth development disorder	Asymptomatic; hypoplasia of tooth or enamel	Impairment correctable with oral surgery	Maldevelopment with impairment not surgically correctable; disabling	-	-
Definition: A disorder characterized by a pathological process of the teeth occurring during tooth development.					
Tooth discoloration	Surface stains	-	-	-	-
Definition: A disorder characterized by a change in tooth hue or tint.					
Toothache	Mild pain	Moderate pain; limiting instrumental ADL	Severe pain; limiting self care ADL	-	-
Definition: A disorder characterized by a sensation of marked discomfort in the tooth.					
Typhlitis	-	-	Symptomatic (e.g., abdominal pain, fever, change in bowel habits with ileus); peritoneal signs	Life-threatening consequences; urgent operative intervention indicated	Death
Definition: A disorder characterized by inflammation of the cecum.					
Upper gastrointestinal hemorrhage	Mild; intervention not indicated	Moderate symptoms; medical intervention or minor cauterization indicated	Transfusion, radiologic, endoscopic, or elective operative intervention indicated	Life-threatening consequences; urgent intervention indicated	Death

Gastrointestinal disorders

	Grade				
**Adverse Event**	**1**	**2**	**3**	**4**	**5**
Definition: A disorder characterized by bleeding from the upper gastrointestinal tract (oral cavity, pharynx, esophagus, and stomach).					
Vomiting	1 - 2 episodes (separated by 5 minutes) in 24 hrs	3 - 5 episodes (separated by 5 minutes) in 24 hrs	>=6 episodes (separated by 5 minutes) in 24 hrs; tube feeding, TPN or hospitalization indicated	Life-threatening consequences; urgent intervention indicated	Death
Definition: A disorder characterized by the reflexive act of ejecting the contents of the stomach through the mouth.					
Gastrointestinal disorders - Other, specify	Asymptomatic or mild symptoms; clinical or diagnostic observations only; intervention not indicated	Moderate; minimal, local or noninvasive intervention indicated; limiting age-appropriate instrumental ADL	Severe or medically significant but not immediately life-threatening; hospitalization or prolongation of existing hospitalization indicated; disabling; limiting self care ADL	Life-threatening consequences; urgent intervention indicated	Death

General disorders and administration site conditions

	Grade				
**Adverse Event**	**1**	**2**	**3**	**4**	**5**
Chills	Mild sensation of cold; shivering; chattering of teeth	Moderate tremor of the entire body; narcotics indicated	Severe or prolonged, not responsive to narcotics	-	-
Definition: A disorder characterized by a sensation of cold that often marks a physiologic response to sweating after a fever.					
Death neonatal	-	-	-	-	Death
Definition: A disorder characterized by cessation of life occurring during the first 28 days of life.					
Death NOS	-	-	-	-	Death
Definition: A cessation of life that cannot be attributed to a CTCAE term associated with Grade 5.					
Edema face	Localized facial edema	Moderate localized facial edema; limiting instrumental ADL	Severe swelling; limiting self care ADL	-	-
Definition: A disorder characterized by swelling due to excessive fluid accumulation in facial tissues.					
Edema limbs	5 - 10% inter-limb discrepancy in volume or circumference at point of greatest visible difference; swelling or obscuration of anatomic architecture on close inspection	>10 - 30% inter-limb discrepancy in volume or circumference at point of greatest visible difference; readily apparent obscuration of anatomic architecture; obliteration of skin folds; readily apparent deviation from normal anatomic contour; limiting instrumental ADL	>30% inter-limb discrepancy in volume; gross deviation from normal anatomic contour; limiting self care ADL	-	-
Definition: A disorder characterized by swelling due to excessive fluid accumulation in the upper or lower extremities.					

General disorders and administration site conditions

	Grade				
**Adverse Event**	**1**	**2**	**3**	**4**	**5**
Edema trunk	Swelling or obscuration of anatomic architecture on close inspection	Readily apparent obscuration of anatomic architecture; obliteration of skin folds; readily apparent deviation from normal anatomic contour; limiting instrumental ADL	Gross deviation from normal anatomic contour; limiting self care ADL	-	-
Definition: A disorder characterized by swelling due to excessive fluid accumulation in the trunk area.					
Facial pain	Mild pain	Moderate pain; limiting instrumental ADL	Severe pain; limiting self care ADL	-	-
Definition: A disorder characterized by a sensation of marked discomfort in the face.					
Fatigue	Fatigue relieved by rest	Fatigue not relieved by rest; limiting instrumental ADL	Fatigue not relieved by rest, limiting self care ADL	-	-
Definition: A disorder characterized by a state of generalized weakness with a pronounced inability to summon sufficient energy to accomplish daily activities.					
Fever	38.0 - 39.0 degrees C (100.4 - 102.2 degrees F)	>39.0 - 40.0 degrees C (102.3 - 104.0 degrees F)	>40.0 degrees C (>104.0 degrees F) for <=24 hrs	>40.0 degrees C (>104.0 degrees F) for >24 hrs	Death
Definition: A disorder characterized by elevation of the body's temperature above the upper limit of normal.					
Flu like symptoms	Mild flu-like symptoms present	Moderate symptoms; limiting instrumental ADL	Severe symptoms; limiting self care ADL	-	-
Definition: A disorder characterized by a group of symptoms similar to those observed in patients with the flu. It includes fever, chills, body aches, malaise, loss of appetite and dry cough.					

General disorders and administration site conditions

	Grade				
**Adverse Event**	**1**	**2**	**3**	**4**	**5**
Gait disturbance	Mild change in gait (e.g., wide-based, limping or hobbling)	Moderate change in gait (e.g., wide-based, limping or hobbling); assistive device indicated; limiting instrumental ADL	Disabling; limiting self care ADL	-	-
Definition: A disorder characterized by walking difficulties.					
Hypothermia	-	35 - >32 degrees C; 95 - >89.6 degrees F	32 - >28 degrees C; 89.6 - >82.4 degrees F	<=28 degrees C; 82.4 degrees F; life-threatening consequences (e.g., coma, hypotension, pulmonary edema, acidemia, ventricular fibrillation)	Death
Definition: A disorder characterized by an abnormally low body temperature. Treatment is required when the body temperature is 35C (95F) or below.					
Infusion related reaction	Mild transient reaction; infusion interruption not indicated; intervention not indicated	Therapy or infusion interruption indicated but responds promptly to symptomatic treatment (e.g., antihistamines, NSAIDS, narcotics, IV fluids); prophylactic medications indicated for <=24 hrs	Prolonged (e.g., not rapidly responsive to symptomatic medication and/or brief interruption of infusion); recurrence of symptoms following initial improvement; hospitalization indicated for clinical sequelae	Life-threatening consequences; urgent intervention indicated	Death
Definition: A disorder characterized by adverse reaction to the infusion of pharmacological or biological substances.					

General disorders and administration site conditions

	Grade				
**Adverse Event**	**1**	**2**	**3**	**4**	**5**
Infusion site extravasation	-	Erythema with associated symptoms (e.g., edema, pain, induration, phlebitis)	Ulceration or necrosis; severe tissue damage; operative intervention indicated	Life-threatening consequences; urgent intervention indicated	Death
Definition: A disorder characterized by leakage of a pharmacologic or a biologic substance from the infusion site into the surrounding tissue. Signs and symptoms include induration, erythema, swelling, burning sensation and marked discomfort at the infusion site.					
Injection site reaction	Tenderness with or without associated symptoms (e.g., warmth, erythema, itching)	Pain; lipodystrophy; edema; phlebitis	Ulceration or necrosis; severe tissue damage; operative intervention indicated	Life-threatening consequences; urgent intervention indicated	Death
Definition: A disorder characterized by an intense adverse reaction (usually immunologic) developing at the site of an injection.					
Irritability	Mild; easily consolable	Moderate; limiting instrumental ADL; increased attention indicated	Severe abnormal or excessive response; limiting self care ADL; inconsolable	-	-
Definition: A disorder characterized by an abnormal responsiveness to stimuli or physiological arousal; may be in response to pain, fright, a drug, an emotional situation or a medical condition.					
Localized edema	Localized to dependent areas, no disability or functional impairment	Moderate localized edema and intervention indicated; limiting instrumental ADL	Severe localized edema and intervention indicated; limiting self care ADL	-	-
Definition: A disorder characterized by swelling due to excessive fluid accumulation at a specific anatomic site.					
Malaise	Uneasiness or lack of well being	Uneasiness or lack of well being; limiting instrumental ADL	-	-	-
Definition: A disorder characterized by a feeling of general discomfort or uneasiness, an out-of-sorts feeling.					

General disorders and administration site conditions

	Grade				
**Adverse Event**	**1**	**2**	**3**	**4**	**5**
Multi-organ failure	-	-	Shock with azotemia and acid-base disturbances; significant coagulation abnormalities	Life-threatening consequences (e.g., vasopressor dependent and oliguric or anuric or ischemic colitis or lactic acidosis)	Death
Definition: A disorder characterized by progressive deterioration of the lungs, liver, kidney and clotting mechanisms.					
Neck edema	Asymptomatic localized neck edema	Moderate neck edema; slight obliteration of anatomic landmarks; limiting instrumental ADL	Generalized neck edema (e.g., difficulty in turning neck); limiting self care ADL	-	-
Definition: A disorder characterized by swelling due to an accumulation of excessive fluid in the neck.					
Non-cardiac chest pain	Mild pain	Moderate pain; limiting instrumental ADL	Severe pain; limiting self care ADL	-	-
Definition: A disorder characterized by discomfort in the chest unrelated to a heart disorder.					
Pain	Mild pain	Moderate pain; limiting instrumental ADL	Severe pain; limiting self care ADL	-	-
Definition: A disorder characterized by the sensation of marked discomfort, distress or agony.					
Sudden death NOS	-	-	-	-	Death
Definition: An unexpected cessation of life that cannot be attributed to a CTCAE term associated with Grade 5.					

General disorders and administration site conditions

	Grade				
**Adverse Event**	**1**	**2**	**3**	**4**	**5**
General disorders and administration site conditions - Other, specify	Asymptomatic or mild symptoms; clinical or diagnostic observations only; intervention not indicated	Moderate; minimal, local or noninvasive intervention indicated; limiting age-appropriate instrumental ADL	Severe or medically significant but not immediately life-threatening; hospitalization or prolongation of existing hospitalization indicated; disabling; limiting self care ADL	Life-threatening consequences; urgent intervention indicated	Death

Hepatobiliary disorders

	Grade				
**Adverse Event**	**1**	**2**	**3**	**4**	**5**
Bile duct stenosis	Asymptomatic; clinical or diagnostic observations only; intervention not indicated	Symptomatic; altered GI function; IV fluids indicated <24 hrs	Severely altered GI function; radiologic, endoscopic or elective operative intervention indicated	Life-threatening consequences; urgent operative intervention indicated	Death
Definition: A disorder characterized by a narrowing of the lumen of the bile duct.					
Biliary fistula	-	Symptomatic and intervention not indicated	Severely altered GI function; TPN indicated; endoscopic intervention indicated; elective operative intervention indicated	Life-threatening consequences; urgent operative intervention indicated	Death
Definition: A disorder characterized by an abnormal communication between the bile ducts and another organ or anatomic site.					
Cholecystitis	-	Symptomatic; medical intervention indicated	Severe symptoms; radiologic, endoscopic or elective operative intervention indicated	Life-threatening consequences; urgent operative intervention indicated	Death
Definition: A disorder characterized by inflammation involving the gallbladder. It may be associated with the presence of gallstones.					
Gallbladder fistula	Asymptomatic clinical or diagnostic observations only; intervention not indicated	Symptomatic and intervention not indicated	Symptomatic or severely altered GI function; TPN indicated; radiologic, endoscopic or elective operative intervention indicated	Life-threatening consequences; urgent operative intervention indicated	Death
Definition: A disorder characterized by an abnormal communication between the gallbladder and another organ or anatomic site.					

Hepatobiliary disorders

	Grade				
**Adverse Event**	**1**	**2**	**3**	**4**	**5**
Gallbladder necrosis	-	-	-	Life-threatening consequences; urgent radiologic or operative intervention indicated	Death
Definition: A disorder characterized by a necrotic process occurring in the gallbladder.					
Gallbladder obstruction	Asymptomatic; clinical or diagnostic observations only; intervention not indicated	Symptomatic; altered GI function; IV fluids indicated <24 hrs	Symptomatic and severely altered GI function; tube feeding, TPN or hospitalization indicated; non-emergent operative intervention indicated	Life-threatening consequences; urgent operative intervention indicated	Death
Definition: A disorder characterized by blockage of the normal flow of the contents of the gallbladder.					
Gallbladder pain	Mild pain	Moderate pain; limiting instrumental ADL	Severe pain; limiting self care ADL	-	-
Definition: A disorder characterized by a sensation of marked discomfort in the gallbladder region.					
Gallbladder perforation	-	-	-	Life-threatening consequences; urgent intervention indicated	Death
Definition: A disorder characterized by a rupture in the gallbladder wall.					
Hepatic failure	-	-	Asterixis; mild encephalopathy; limiting self care ADL	Moderate to severe encephalopathy; coma; life-threatening consequences	Death

Hepatobiliary disorders

	Grade				
**Adverse Event**	**1**	**2**	**3**	**4**	**5**
Definition: A disorder characterized by the inability of the liver to metabolize chemicals in the body. Laboratory test results reveal abnormal plasma levels of ammonia, bilirubin, lactic dehydrogenase, and alkaline phosphatase.					
Hepatic hemorrhage	Mild; intervention not indicated	Symptomatic; medical intervention indicated	Transfusion indicated	Life-threatening consequences; urgent intervention indicated	Death
Definition: A disorder characterized by bleeding from the liver.					
Hepatic necrosis	-	-	-	Life-threatening consequences; urgent radiologic or operative intervention indicated	Death
Definition: A disorder characterized by a necrotic process occurring in the hepatic parenchyma.					
Hepatic pain	Mild pain	Moderate pain; limiting instrumental ADL	Severe pain; limiting self care ADL	-	-
Definition: A disorder characterized by a sensation of marked discomfort in the liver region.					
Perforation bile duct	-	-	Radiologic, endoscopic or elective operative intervention indicated	Life-threatening consequences; urgent operative intervention indicated	Death
Definition: A disorder characterized by a rupture in the wall of the extrahepatic or intrahepatic bile duct.					
Portal hypertension	-	Decreased portal vein flow	Reversal/retrograde portal vein flow; associated with varices and/or ascites	Life-threatening consequences; urgent intervention indicated	Death

Hepatobiliary disorders

	Grade				
**Adverse Event**	**1**	**2**	**3**	**4**	**5**
Definition: A disorder characterized by an increase in blood pressure in the portal venous system.					
Portal vein thrombosis	-	Intervention not indicated	Medical intervention indicated	Life-threatening consequences; urgent intervention indicated	Death
Definition: A disorder characterized by the formation of a thrombus (blood clot) in the portal vein.					
Hepatobiliary disorders - Other, specify	Asymptomatic or mild symptoms; clinical or diagnostic observations only; intervention not indicated	Moderate; minimal, local or noninvasive intervention indicated; limiting age-appropriate instrumental ADL	Severe or medically significant but not immediately life-threatening; hospitalization or prolongation of existing hospitalization indicated; disabling; limiting self care ADL	Life-threatening consequences; urgent intervention indicated	Death

Immune system disorders

	Grade				
**Adverse Event**	**1**	**2**	**3**	**4**	**5**
Allergic reaction	Transient flushing or rash, drug fever <38 degrees C (<100.4 degrees F); intervention not indicated	Intervention or infusion interruption indicated; responds promptly to symptomatic treatment (e.g., antihistamines, NSAIDS, narcotics); prophylactic medications indicated for <=24 hrs	Prolonged (e.g., not rapidly responsive to symptomatic medication and/or brief interruption of infusion); recurrence of symptoms following initial improvement; hospitalization indicated for clinical sequelae (e.g., renal impairment, pulmonary infiltrates)	Life-threatening consequences; urgent intervention indicated	Death
Definition: A disorder characterized by an adverse local or general response from exposure to an allergen.					
Anaphylaxis	-	-	Symptomatic bronchospasm, with or without urticaria; parenteral intervention indicated; allergy-related edema/angioedema; hypotension	Life-threatening consequences; urgent intervention indicated	Death
Definition: A disorder characterized by an acute inflammatory reaction resulting from the release of histamine and histamine-like substances from mast cells, causing a hypersensitivity immune response. Clinically, it presents with breathing difficulty, dizziness, hypotension, cyanosis and loss of consciousness and may lead to death.					
Autoimmune disorder	Asymptomatic; serologic or other evidence of autoimmune reaction, with normal organ function; intervention not indicated	Evidence of autoimmune reaction involving a non-essential organ or function (e.g., hypothyroidism)	Autoimmune reactions involving major organ (e.g., colitis, anemia, myocarditis, kidney)	Life-threatening consequences; urgent intervention indicated	Death

Immune system disorders

	Grade				
**Adverse Event**	**1**	**2**	**3**	**4**	**5**
Definition: A disorder resulting from loss of function or tissue destruction of an organ or multiple organs, arising from humoral or cellular immune responses of the individual to his own tissue constituents.					
Cytokine release syndrome	Mild reaction; infusion interruption not indicated; intervention not indicated	Therapy or infusion interruption indicated but responds promptly to symptomatic treatment (e.g., antihistamines, NSAIDS, narcotics, IV fluids); prophylactic medications indicated for <=24 hrs	Prolonged (e.g., not rapidly responsive to symptomatic medication and/or brief interruption of infusion); recurrence of symptoms following initial improvement; hospitalization indicated for clinical sequelae (e.g., renal impairment, pulmonary infiltrates)	Life-threatening consequences; pressor or ventilatory support indicated	Death
Definition: A disorder characterized by nausea, headache, tachycardia, hypotension, rash, and shortness of breath; it is caused by the release of cytokines from the cells.					
Serum sickness	Asymptomatic; clinical or diagnostic observations only; intervention not indicated	Moderate arthralgia; fever, rash, urticaria, antihistamines indicated	Severe arthralgia or arthritis; extensive rash; steroids or IV fluids indicated	Life-threatening consequences; pressor or ventilatory support indicated	Death
Definition: A disorder characterized by a delayed-type hypersensitivity reaction to foreign proteins derived from an animal serum. It occurs approximately six to twenty-one days following the administration of the foreign antigen. Symptoms include fever, arthralgias, myalgias, skin eruptions, lymphadenopathy, chest marked discomfort and dyspnea.					

Immune system disorders

	Grade				
**Adverse Event**	**1**	**2**	**3**	**4**	**5**
Immune system disorders - Other, specify	Asymptomatic or mild symptoms; clinical or diagnostic observations only; intervention not indicated	Moderate; minimal, local or noninvasive intervention indicated; limiting age-appropriate instrumental ADL	Severe or medically significant but not immediately life-threatening; hospitalization or prolongation of existing hospitalization indicated; disabling; limiting self care ADL	Life-threatening consequences; urgent intervention indicated	Death

Infections and infestations

	Grade				
**Adverse Event**	**1**	**2**	**3**	**4**	**5**
Abdominal infection	-	-	IV antibiotic, antifungal, or antiviral intervention indicated; radiologic or operative intervention indicated	Life-threatening consequences; urgent intervention indicated	Death
Definition: A disorder characterized by an infectious process involving the abdominal cavity.					
Anorectal infection	Localized; local intervention indicated	Oral intervention indicated (e.g., antibiotic, antifungal, antiviral)	IV antibiotic, antifungal, or antiviral intervention indicated; radiologic, endoscopic, or operative intervention indicated	Life-threatening consequences; urgent intervention indicated	Death
Definition: A disorder characterized by an infectious process involving the anal area and the rectum.					
Appendicitis	-	-	IV antibiotic, antifungal, or antiviral intervention indicated; radiologic or operative intervention indicated	Life-threatening consequences; urgent intervention indicated	Death
Definition: A disorder characterized by acute inflammation to the vermiform appendix caused by a pathogenic agent.					
Appendicitis perforated	-	Symptomatic; medical intervention indicated	Severe symptoms; elective operative intervention indicated	Life-threatening consequences; urgent intervention indicated	Death
Definition: A disorder characterized by acute inflammation to the vermiform appendix caused by a pathogenic agent with gangrenous changes resulting in the rupture of the appendiceal wall. The appendiceal wall rupture causes the release of inflammatory and bacterial contents from the appendiceal lumen into the abdominal cavity.					

Infections and infestations					
	Grade				
**Adverse Event**	**1**	**2**	**3**	**4**	**5**
Arteritis infective	-	-	IV antibiotic, antifungal, or antiviral intervention indicated; radiologic or operative intervention indicated	Life-threatening consequences; urgent intervention indicated	Death
Definition: A disorder characterized by an infectious process involving an artery.					
Biliary tract infection	-	-	IV antibiotic, antifungal, or antiviral intervention indicated; radiologic or operative intervention indicated	Life-threatening consequences; urgent intervention indicated	Death
Definition: A disorder characterized by an infectious process involving the biliary tract.					
Bladder infection	-	Oral intervention indicated (e.g., antibiotic, antifungal, antiviral)	IV antibiotic, antifungal, or antiviral intervention indicated; radiologic, endoscopic, or operative intervention indicated	Life-threatening consequences; urgent intervention indicated	Death
Definition: A disorder characterized by an infectious process involving the bladder.					
Bone infection	-	-	IV antibiotic, antifungal, or antiviral intervention indicated; radiologic or operative intervention indicated	Life-threatening consequences; urgent intervention indicated	Death
Definition: A disorder characterized by an infectious process involving the bones.					

Infections and infestations

	Grade				
**Adverse Event**	**1**	**2**	**3**	**4**	**5**
Breast infection	-	Local infection with moderate symptoms; oral intervention indicated (e.g., antibiotic, antifungal, antiviral)	Severe infection; axillary adenitis; IV antibacterial, antifungal, or antiviral intervention indicated	Life-threatening consequences; urgent intervention indicated	Death
Definition: A disorder characterized by an infectious process involving the breast.					
Bronchial infection	-	Moderate symptoms; oral intervention indicated (e.g., antibiotic, antifungal, antiviral)	IV antibiotic, antifungal, or antiviral intervention indicated; radiologic, endoscopic, or operative intervention indicated	Life-threatening consequences; urgent intervention indicated	Death
Definition: A disorder characterized by an infectious process involving the bronchi.					
Catheter related infection	-	Localized; local intervention indicated; oral intervention indicated (e.g., antibiotic, antifungal, antiviral)	IV antibiotic, antifungal, or antiviral intervention indicated; radiologic or operative intervention indicated	Life-threatening consequences; urgent intervention indicated	Death
Definition: A disorder characterized by an infectious process that arises secondary to catheter use.					
Cecal infection	-	-	IV antibiotic, antifungal, or antiviral intervention indicated; radiologic, endoscopic, or operative intervention indicated	Life-threatening consequences; urgent intervention indicated	Death
Definition: A disorder characterized by an infectious process involving the cecum.					

Infections and infestations

	Grade				
**Adverse Event**	**1**	**2**	**3**	**4**	**5**
Cervicitis infection	-	Localized; local intervention indicated (e.g., topical antibiotic, antifungal, or antiviral)	IV antibiotic, antifungal, or antiviral intervention indicated; radiologic or operative intervention indicated	Life-threatening consequences; urgent intervention indicated	Death
Definition: A disorder characterized by an infectious process involving the uterine cervix.					
Conjunctivitis infective	-	Localized; local intervention indicated (e.g., topical antibiotic, antifungal, or antiviral)	IV antibiotic, antifungal, or antiviral intervention indicated; radiologic or operative intervention indicated	Life-threatening consequences; urgent intervention indicated	Death
Definition: A disorder characterized by an infectious process involving the conjunctiva. Clinical manifestations include pink or red color in the eyes.					
Corneal infection	-	Localized; local intervention indicated (e.g., topical antibiotic, antifungal, or antiviral)	IV antibiotic, antifungal, or antiviral intervention indicated; radiologic or operative intervention indicated	Life-threatening consequences; urgent intervention indicated	Death
Definition: A disorder characterized by an infectious process involving the cornea.					
Cranial nerve infection	-	-	IV antibiotic, antifungal, or antiviral intervention indicated; radiologic or operative intervention indicated	Life-threatening consequences; urgent intervention indicated	Death
Definition: A disorder characterized by an infectious process involving a cranial nerve.					

Infections and infestations

	Grade				
**Adverse Event**	**1**	**2**	**3**	**4**	**5**
Device related infection	-	-	IV antibiotic, antifungal, or antiviral intervention indicated; radiologic or operative intervention indicated	Life-threatening consequences; urgent intervention indicated	Death
Definition: A disorder characterized by an infectious process involving the use of a medical device.					
Duodenal infection	-	Moderate symptoms; medical intervention indicated (e.g., oral antibiotics)	IV antibiotic, antifungal, or antiviral intervention indicated; radiologic or operative intervention indicated	Life-threatening consequences; urgent intervention indicated	Death
Definition: A disorder characterized by an infectious process involving the duodenum.					
Encephalitis infection	-	-	IV antibiotic, antifungal, or antiviral intervention indicated; severe changes in mental status; self-limited seizure activity; focal neurologic abnormalities	Life-threatening consequences; urgent intervention indicated	Death
Definition: A disorder characterized by an infectious process involving the brain tissue.					
Encephalomyelitis infection	-	-	IV antibiotic, antifungal, or antiviral intervention indicated; radiologic or operative intervention indicated	Life-threatening consequences; urgent intervention indicated	Death
Definition: A disorder characterized by an infectious process involving the brain and spinal cord tissues.					

Infections and infestations

	Grade				
**Adverse Event**	**1**	**2**	**3**	**4**	**5**
Endocarditis infective	-	-	IV antibiotic, antifungal, or antiviral intervention indicated; radiologic or operative intervention indicated	Life-threatening consequences; urgent intervention indicated	Death
Definition: A disorder characterized by an infectious process involving the endocardial layer of the heart.					
Endophthalmitis	-	Local intervention indicated	Systemic intervention or hospitalization indicated	Blindness (20/200 or worse)	-
Definition: A disorder characterized by an infectious process involving the internal structures of the eye.					
Enterocolitis infectious	-	Passage of >3 unformed stools per 24 hrs or duration of illness >48 hrs; moderate abdominal pain	IV antibiotic, antifungal, or antiviral intervention indicated; radiologic, endoscopic, or operative intervention indicated; profuse watery diarrhea with signs of hypovolemia; bloody diarrhea; fever; severe abdominal pain; hospitalization indicated	Life-threatening consequences; urgent intervention indicated	Death
Definition: A disorder characterized by an infectious process involving the small and large intestines.					
Esophageal infection	-	Local intervention indicated (e.g., oral antibiotic, antifungal, antiviral)	IV antibiotic, antifungal, or antiviral intervention indicated; radiologic or operative intervention indicated	Life-threatening consequences; urgent intervention indicated	Death

Infections and infestations

	Grade				
**Adverse Event**	**1**	**2**	**3**	**4**	**5**
Definition: A disorder characterized by an infectious process involving the esophagus.					
Eye infection	-	Localized; local intervention indicated (e.g., topical antibiotic, antifungal, or antiviral)	IV antibiotic, antifungal, or antiviral intervention indicated; radiologic or operative intervention indicated	Life-threatening consequences; urgent intervention indicated; enucleation	Death
Definition: A disorder characterized by an infectious process involving the eye.					
Gallbladder infection	-	-	IV antibiotic, antifungal, or antiviral intervention indicated; radiologic, endoscopic, or operative intervention indicated	Life-threatening consequences; urgent intervention indicated	Death
Definition: A disorder characterized by an infectious process involving the gallbladder.					
Gum infection	Local therapy indicated (swish and swallow)	Moderate symptoms; oral intervention indicated (e.g., antibiotic, antifungal, antiviral)	IV antibiotic, antifungal, or antiviral intervention indicated; radiologic or operative intervention indicated	Life-threatening consequences; urgent intervention indicated	Death
Definition: A disorder characterized by an infectious process involving the gums.					
Hepatic infection	-	-	IV antibiotic, antifungal, or antiviral intervention indicated; radiologic or operative intervention indicated	Life-threatening consequences; urgent intervention indicated	Death
Definition: A disorder characterized by an infectious process involving the liver.					

Infections and infestations

	Grade				
**Adverse Event**	**1**	**2**	**3**	**4**	**5**
Hepatitis viral	Asymptomatic, treatment not indicated	-	Symptomatic liver dysfunction; fibrosis by biopsy; compensated cirrhosis; reactivation of chronic hepatitis	Decompensated liver function (e.g., ascites, coagulopathy, encephalopathy, coma)	Death
Definition: A disorder characterized by a viral pathologic process involving the liver parenchyma.					
Infective myositis	-	Localized; local intervention indicated (e.g., topical antibiotic, antifungal, or antiviral)	IV antibiotic, antifungal, or antiviral intervention indicated; radiologic or operative intervention indicated	Life-threatening consequences; urgent intervention indicated	Death
Definition: A disorder characterized by an infectious process involving the skeletal muscles.					
Joint infection	-	Localized; local intervention indicated; oral intervention indicated (e.g., antibiotic, antifungal, antiviral); needle aspiration indicated (single or multiple)	Arthroscopic intervention indicated (e.g., drainage) or arthrotomy (e.g., open surgical drainage)	Life-threatening consequences; urgent intervention indicated	Death
Definition: A disorder characterized by an infectious process involving a joint.					
Kidney infection	-	-	IV antibiotic, antifungal, or antiviral intervention indicated; radiologic, endoscopic, or operative intervention indicated	Life-threatening consequences; urgent intervention indicated	Death

Infections and infestations

	Grade				
**Adverse Event**	**1**	**2**	**3**	**4**	**5**
Definition: A disorder characterized by an infectious process involving the kidney.					
Laryngitis	-	Moderate symptoms; oral intervention indicated (e.g., antibiotic, antifungal, antiviral)	IV antibiotic, antifungal, or antiviral intervention indicated; radiologic or operative intervention indicated	Life-threatening consequences; urgent intervention indicated	Death
Definition: A disorder characterized by an inflammatory process involving the larynx.					
Lip infection	Localized, local intervention indicated	Oral intervention indicated (e.g., antibiotic, antifungal, antiviral)	IV antibiotic, antifungal, or antiviral intervention indicated; radiologic or operative intervention indicated	-	-
Definition: A disorder characterized by an infectious process involving the lips.					
Lung infection	-	Moderate symptoms; oral intervention indicated (e.g., antibiotic, antifungal, antiviral)	IV antibiotic, antifungal, or antiviral intervention indicated; radiologic, endoscopic, or operative intervention indicated	Life-threatening consequences; urgent intervention indicated	Death
Definition: A disorder characterized by an infectious process involving the lungs.					
Lymph gland infection	-	Localized; local intervention indicated (e.g., topical antibiotic, antifungal, or antiviral)	IV antibiotic, antifungal, or antiviral intervention indicated; radiologic or operative intervention indicated	Life-threatening consequences; urgent intervention indicated	Death
Definition: A disorder characterized by an infectious process involving the lymph nodes.					

Infections and infestations

	Grade				
**Adverse Event**	**1**	**2**	**3**	**4**	**5**
Mediastinal infection	-	-	IV antibiotic, antifungal, or antiviral intervention indicated; radiologic or operative intervention indicated	Life-threatening consequences; urgent intervention indicated	Death
Definition: A disorder characterized by an infectious process involving the mediastinum.					
Meningitis	-	-	IV antibiotic, antifungal, or antiviral intervention indicated; radiologic or operative intervention indicated; focal neurologic deficit	Life-threatening consequences; urgent intervention indicated	Death
Definition: A disorder characterized by acute inflammation of the meninges of the brain and/or spinal cord.					
Mucosal infection	Localized, local intervention indicated	Oral intervention indicated (e.g., antibiotic, antifungal, antiviral)	IV antibiotic, antifungal, or antiviral intervention indicated; radiologic or operative intervention indicated	Life-threatening consequences; urgent intervention indicated	Death
Definition: A disorder characterized by an infectious process involving a mucosal surface.					
Nail infection	Localized, local intervention indicated	Oral intervention indicated (e.g., antibiotic, antifungal, antiviral)	IV antibiotic, antifungal, or antiviral intervention indicated; radiologic or operative intervention indicated	-	-
Definition: A disorder characterized by an infectious process involving the nail.					

Infections and infestations

	Grade				
**Adverse Event**	**1**	**2**	**3**	**4**	**5**
Otitis externa	-	Localized; local intervention indicated (e.g., topical antibiotic, antifungal, or antiviral)	IV antibiotic, antifungal, or antiviral intervention indicated; radiologic or operative intervention indicated	Life-threatening consequences; urgent intervention indicated	Death
Definition: A disorder characterized by an infectious process involving the outer ear and ear canal. Contributory factors include excessive water exposure (swimmer's ear infection) and cuts in the ear canal. Symptoms include fullness, itching, swelling and marked discomfort in the ear and ear drainage.					
Otitis media	-	Localized; local intervention indicated (e.g., topical antibiotic, antifungal, or antiviral)	IV antibiotic, antifungal, or antiviral intervention indicated; radiologic or operative intervention indicated	Life-threatening consequences; urgent intervention indicated	Death
Definition: A disorder characterized by an infectious process involving the middle ear.					
Ovarian infection	-	Localized; local intervention indicated (e.g., topical antibiotic, antifungal, or antiviral)	IV antibiotic, antifungal, or antiviral intervention indicated; radiologic or operative intervention indicated	Life-threatening consequences; urgent intervention indicated	Death
Definition: A disorder characterized by an infectious process involving the ovary.					
Pancreas infection	-	-	IV antibiotic, antifungal, or antiviral intervention indicated; radiologic or operative intervention indicated	Life-threatening consequences; urgent intervention indicated	Death
Definition: A disorder characterized by an infectious process involving the pancreas.					

Infections and infestations

	Grade				
**Adverse Event**	**1**	**2**	**3**	**4**	**5**
Papulopustular rash	Papules and/or pustules covering <10% BSA, which may or may not be associated with symptoms of pruritus or tenderness	Papules and/or pustules covering 10-30% BSA, which may or may not be associated with symptoms of pruritus or tenderness; associated with psychosocial impact; limiting instrumental ADL	Papules and/or pustules covering >30% BSA, which may or may not be associated with symptoms of pruritus or tenderness; limiting self-care ADL; associated with local superinfection with oral antibiotics indicated	Papules and/or pustules covering any % BSA, which may or may not be associated with symptoms of pruritus or tenderness and are associated with extensive superinfection with IV antibiotics indicated; life-threatening consequences	Death
Definition: A disorder characterized by an eruption consisting of papules (a small, raised pimple) and pustules (a small pus filled blister), typically appearing in face, scalp, and upper chest and back Unlike acne, this rash does not present with whiteheads or blackheads, and can be symptomatic, with itchy or tender lesions.					
Paronychia	Nail fold edema or erythema; disruption of the cuticle	Localized intervention indicated; oral intervention indicated (e.g., antibiotic, antifungal, antiviral); nail fold edema or erythema with pain; associated with discharge or nail plate separation; limiting instrumental ADL	Surgical intervention or IV antibiotics indicated; limiting self care ADL	-	-
Definition: A disorder characterized by an infectious process involving the soft tissues around the nail.					
Pelvic infection	-	Moderate symptoms; oral intervention indicated (e.g., antibiotic, antifungal, antiviral)	IV antibiotic, antifungal, or antiviral intervention indicated; radiologic or operative intervention indicated	Life-threatening consequences; urgent intervention indicated	Death

Infections and infestations

	Grade				
Adverse Event	1	2	3	4	5
Definition: A disorder characterized by an infectious process involving the pelvic cavity.					
Penile infection	-	Localized; local intervention indicated (e.g., topical antibiotic, antifungal, or antiviral)	IV antibiotic, antifungal, or antiviral intervention indicated; radiologic or operative intervention indicated	Life-threatening consequences; urgent intervention indicated	Death
Definition: A disorder characterized by an infectious process involving the penis.					
Periorbital infection	-	Localized; local intervention indicated (e.g., topical antibiotic, antifungal, or antiviral)	IV antibiotic, antifungal, or antiviral intervention indicated; radiologic or operative intervention indicated	Life-threatening consequences; urgent intervention indicated	Death
Definition: A disorder characterized by an infectious process involving the orbit of the eye.					
Peripheral nerve infection	-	Localized; local intervention indicated (e.g., topical antibiotic, antifungal, or antiviral)	IV antibiotic, antifungal, or antiviral intervention indicated; radiologic or operative intervention indicated	Life-threatening consequences; urgent intervention indicated	Death
Definition: A disorder characterized by an infectious process involving the peripheral nerves.					
Peritoneal infection	-	-	IV antibiotic, antifungal, or antiviral intervention indicated; radiologic, endoscopic, or operative intervention indicated	Life-threatening consequences; urgent intervention indicated	Death
Definition: A disorder characterized by an infectious process involving the peritoneum.					

Infections and infestations

	Grade				
**Adverse Event**	**1**	**2**	**3**	**4**	**5**
Pharyngitis	-	Localized; local intervention indicated (e.g., topical antibiotic, antifungal, or antiviral)	IV antibiotic, antifungal, or antiviral intervention indicated; radiologic or operative intervention indicated	Life-threatening consequences; urgent intervention indicated	Death
Definition: A disorder characterized by inflammation of the throat.					
Phlebitis infective	-	Localized; local intervention indicated (e.g., topical antibiotic, antifungal, or antiviral)	IV antibiotic, antifungal, or antiviral intervention indicated; radiologic or operative intervention indicated	Life-threatening consequences; urgent intervention indicated	Death
Definition: A disorder characterized by an infectious process involving the vein. Clinical manifestations include erythema, marked discomfort, swelling, and induration along the course of the infected vein.					
Pleural infection	-	Localized; local intervention indicated (e.g., topical antibiotic, antifungal, or antiviral)	IV antibiotic, antifungal, or antiviral intervention indicated; radiologic, endoscopic, or operative intervention indicated	Life-threatening consequences; urgent intervention indicated	Death
Definition: A disorder characterized by an infectious process involving the pleura.					
Prostate infection	-	Moderate symptoms; oral intervention indicated (e.g., antibiotic, antifungal, antiviral)	IV antibiotic, antifungal, or antiviral intervention indicated; radiologic, endoscopic, or operative intervention indicated	Life-threatening consequences; urgent intervention indicated	Death
Definition: A disorder characterized by an infectious process involving the prostate gland.					

Infections and infestations

	Grade				
**Adverse Event**	**1**	**2**	**3**	**4**	**5**
Rash pustular	-	Localized; local intervention indicated (e.g., topical antibiotic, antifungal, or antiviral)	IV antibiotic, antifungal, or antiviral intervention indicated; radiologic or operative intervention indicated	-	-
Definition: A disorder characterized by a circumscribed and elevated skin lesion filled with pus.					
Rhinitis infective	-	Localized; local intervention indicated (e.g., topical antibiotic, antifungal, or antiviral)	-	-	-
Definition: A disorder characterized by an infectious process involving the nasal mucosal.					
Salivary gland infection	-	Moderate symptoms; oral intervention indicated (e.g., antibiotic, antifungal, antiviral)	IV antibiotic, antifungal, or antiviral intervention indicated; radiologic or operative intervention indicated	Life-threatening consequences; urgent intervention indicated	Death
Definition: A disorder characterized by an infectious process involving the salivary gland.					
Scrotal infection	-	Localized; local intervention indicated (e.g., topical antibiotic, antifungal, or antiviral)	IV antibiotic, antifungal, or antiviral intervention indicated; radiologic or operative intervention indicated	Life-threatening consequences; urgent intervention indicated	Death
Definition: A disorder characterized by an infectious process involving the scrotum.					
Sepsis	-	-	-	Life-threatening consequences; urgent intervention indicated	Death

Infections and infestations

	Grade				
**Adverse Event**	**1**	**2**	**3**	**4**	**5**
Definition: A disorder characterized by the presence of pathogenic microorganisms in the blood stream that cause a rapidly progressing systemic reaction that may lead to shock.					
Sinusitis	-	Localized; local intervention indicated (e.g., topical antibiotic, antifungal, or antiviral)	IV antibiotic, antifungal, or antiviral intervention indicated; radiologic, endoscopic, or operative intervention indicated	Life-threatening consequences; urgent intervention indicated	Death
Definition: A disorder characterized by an infectious process involving the mucous membranes of the paranasal sinuses.					
Skin infection	Localized, local intervention indicated	Oral intervention indicated (e.g., antibiotic, antifungal, antiviral)	IV antibiotic, antifungal, or antiviral intervention indicated; radiologic or operative intervention indicated	Life-threatening consequences; urgent intervention indicated	Death
Definition: A disorder characterized by an infectious process involving the skin.					
Small intestine infection	-	Moderate symptoms; oral intervention indicated (e.g., antibiotic, antifungal, antiviral)	IV antibiotic, antifungal, or antiviral intervention indicated; radiologic or operative intervention indicated	Life-threatening consequences; urgent intervention indicated	Death
Definition: A disorder characterized by an infectious process involving the small intestine.					
Soft tissue infection	-	Localized; local intervention indicated (e.g., topical antibiotic, antifungal, or antiviral)	IV antibiotic, antifungal, or antiviral intervention indicated; radiologic or operative intervention indicated	Life-threatening consequences; urgent intervention indicated	Death
Definition: A disorder characterized by an infectious process involving soft tissues.					

Infections and infestations

	Grade				
**Adverse Event**	**1**	**2**	**3**	**4**	**5**
Splenic infection	-	-	IV antibiotic, antifungal, or antiviral intervention indicated; radiologic or operative intervention indicated	Life-threatening consequences; urgent intervention indicated	Death
Definition: A disorder characterized by an infectious process involving the spleen.					
Stoma site infection	Localized, local intervention indicated	Oral intervention indicated (e.g., antibiotic, antifungal, antiviral)	IV antibiotic, antifungal, or antiviral intervention indicated; radiologic, endoscopic, or operative intervention indicated	Life-threatening consequences; urgent intervention indicated	Death
Definition: A disorder characterized by an infectious process involving a stoma (surgically created opening on the surface of the body).					
Tooth infection	-	Localized; local intervention indicated (e.g., topical antibiotic, antifungal, or antiviral)	IV antibiotic, antifungal, or antiviral intervention indicated; radiologic or operative intervention indicated	Life-threatening consequences; urgent intervention indicated	Death
Definition: A disorder characterized by an infectious process involving a tooth.					
Tracheitis	-	Moderate symptoms; oral intervention indicated (e.g., antibiotic, antifungal, antiviral)	IV antibiotic, antifungal, or antiviral intervention indicated; radiologic, endoscopic, or operative intervention indicated	Life-threatening consequences; urgent intervention indicated	Death
Definition: A disorder characterized by an infectious process involving the trachea.					

Infections and infestations

	Grade				
**Adverse Event**	**1**	**2**	**3**	**4**	**5**
Upper respiratory infection	-	Moderate symptoms; oral intervention indicated (e.g., antibiotic, antifungal, antiviral)	IV antibiotic, antifungal, or antiviral intervention indicated; radiologic, endoscopic, or operative intervention indicated	Life-threatening consequences; urgent intervention indicated	Death
Definition: A disorder characterized by an infectious process involving the upper respiratory tract (nose, paranasal sinuses, pharynx, larynx, or trachea).					
Urethral infection	-	Localized; local intervention indicated (e.g., topical antibiotic, antifungal, or antiviral)	IV antibiotic, antifungal, or antiviral intervention indicated; radiologic, endoscopic, or operative intervention indicated	Life-threatening consequences; urgent intervention indicated	Death
Definition: A disorder characterized by an infectious process involving the urethra.					
Urinary tract infection	-	Localized; local intervention indicated (e.g., topical antibiotic, antifungal, or antiviral)	IV antibiotic, antifungal, or antiviral intervention indicated; radiologic or operative intervention indicated	Life-threatening consequences; urgent intervention indicated	Death
Definition: A disorder characterized by an infectious process involving the urinary tract, most commonly the bladder and the urethra.					
Uterine infection	-	Moderate symptoms; oral intervention indicated (e.g., antibiotic, antifungal, antiviral)	IV antibiotic, antifungal, or antiviral intervention indicated; radiologic or operative intervention indicated	Life-threatening consequences; urgent intervention indicated	Death
Definition: A disorder characterized by an infectious process involving the endometrium. It may extend to the myometrium and parametrial tissues.					

Infections and infestations

	Grade				
**Adverse Event**	**1**	**2**	**3**	**4**	**5**
Vaginal infection	-	Localized; local intervention indicated (e.g., topical antibiotic, antifungal, or antiviral)	IV antibiotic, antifungal, or antiviral intervention indicated; radiologic or operative intervention indicated	Life-threatening consequences; urgent intervention indicated	Death
Definition: A disorder characterized by an infectious process involving the vagina.					
Vulval infection	Localized, local intervention indicated	Oral intervention indicated (e.g., antibiotic, antifungal, antiviral)	IV antibiotic, antifungal, or antiviral intervention indicated; radiologic or operative intervention indicated	Life-threatening consequences; urgent intervention indicated	Death
Definition: A disorder characterized by an infectious process involving the vulva.					
Wound infection	-	Localized; local intervention indicated (e.g., topical antibiotic, antifungal, or antiviral)	IV antibiotic, antifungal, or antiviral intervention indicated; radiologic or operative intervention indicated	Life-threatening consequences; urgent intervention indicated	Death
Definition: A disorder characterized by an infectious process involving the wound.					
Infections and infestations - Other, specify	Asymptomatic or mild symptoms; clinical or diagnostic observations only; intervention not indicated	Moderate; minimal, local or noninvasive intervention indicated; limiting age-appropriate instrumental ADL	Severe or medically significant but not immediately life-threatening; hospitalization or prolongation of existing hospitalization indicated; disabling; limiting self care ADL	Life-threatening consequences; urgent intervention indicated	Death

Injury, poisoning and procedural complications

	Grade				
**Adverse Event**	**1**	**2**	**3**	**4**	**5**
Ankle fracture	Mild; non-surgical intervention indicated	Limiting instrumental ADL; operative intervention indicated	Limiting self care ADL; elective surgery indicated	-	-
Definition: A finding of damage to the ankle joint characterized by a break in the continuity of the ankle bone. Symptoms include marked discomfort, swelling and difficulty moving the affected leg and foot.					
Aortic injury	-	-	Severe symptoms; limiting self care ADL; disabling; repair or revision indicated	Life-threatening consequences; evidence of end organ damage; urgent operative intervention indicated	Death
Definition: A finding of damage to the aorta.					
Arterial injury	Asymptomatic diagnostic finding; intervention not indicated	Symptomatic (e.g., claudication); repair or revision not indicated	Severe symptoms; limiting self care ADL; disabling; repair or revision indicated	Life-threatening consequences; evidence of end organ damage; urgent operative intervention indicated	Death
Definition: A finding of damage to an artery.					
Biliary anastomotic leak	Asymptomatic diagnostic observations only; intervention not indicated	Symptomatic; medical intervention indicated	Severe symptoms; radiologic, endoscopic or elective operative intervention indicated	Life-threatening consequences; urgent operative intervention indicated	Death
Definition: A finding of leakage of bile due to breakdown of a biliary anastomosis (surgical connection of two separate anatomic structures).					

Injury, poisoning and procedural complications

	Grade				
**Adverse Event**	**1**	**2**	**3**	**4**	**5**
Bladder anastomotic leak	Asymptomatic diagnostic observations only; intervention not indicated	Symptomatic; medical intervention indicated	Severe symptoms; radiologic, endoscopic or elective operative intervention indicated	Life-threatening consequences; urgent operative intervention indicated	Death
Definition: A finding of leakage of urine due to breakdown of a bladder anastomosis (surgical connection of two separate anatomic structures).					
Bruising	Localized or in a dependent area	Generalized	-	-	-
Definition: A finding of injury of the soft tissues or bone characterized by leakage of blood into surrounding tissues.					
Burn	Minimal symptoms; intervention not indicated	Medical intervention; minimal debridement indicated	Moderate to major debridement or reconstruction indicated	Life-threatening consequences	Death
Definition: A finding of impaired integrity to the anatomic site of an adverse thermal reaction. Burns can be caused by exposure to chemicals, direct heat, electricity, flames and radiation. The extent of damage depends on the length and intensity of exposure and time until provision of treatment.					
Dermatitis radiation	Faint erythema or dry desquamation	Moderate to brisk erythema; patchy moist desquamation, mostly confined to skin folds and creases; moderate edema	Moist desquamation in areas other than skin folds and creases; bleeding induced by minor trauma or abrasion	Life-threatening consequences; skin necrosis or ulceration of full thickness dermis; spontaneous bleeding from involved site; skin graft indicated	Death
Definition: A finding of cutaneous inflammatory reaction occurring as a result of exposure to biologically effective levels of ionizing radiation.					

Injury, poisoning and procedural complications

	Grade				
**Adverse Event**	**1**	**2**	**3**	**4**	**5**
Esophageal anastomotic leak	Asymptomatic diagnostic observations only; intervention not indicated	Symptomatic; medical intervention indicated	Severe symptoms; radiologic, endoscopic or elective operative intervention indicated	Life-threatening consequences; urgent operative intervention indicated	Death
Definition: A finding of leakage due to breakdown of an esophageal anastomosis (surgical connection of two separate anatomic structures).					
Fall	Minor with no resultant injuries; intervention not indicated	Symptomatic; noninvasive intervention indicated	Hospitalization indicated	-	-
Definition: A finding of sudden movement downward, usually resulting in injury.					
Fallopian tube anastomotic leak	Asymptomatic; clinical or diagnostic observations only; intervention not indicated	Symptomatic; medical intervention indicated	Severe symptoms; radiologic, endoscopic or elective operative intervention indicated	Life-threatening consequences; urgent operative intervention indicated	Death
Definition: A finding of leakage due to breakdown of a fallopian tube anastomosis (surgical connection of two separate anatomic structures).					
Fallopian tube perforation	Asymptomatic diagnostic observations only; intervention not indicated	Symptomatic and intervention not indicated	Severe symptoms; elective operative intervention indicated	Life-threatening consequences; urgent operative intervention indicated (e.g., organ resection)	Death
Definition: A finding of rupture of the fallopian tube wall.					

Injury, poisoning and procedural complications

	Grade				
**Adverse Event**	**1**	**2**	**3**	**4**	**5**
Fracture	Asymptomatic; clinical or diagnostic observations only; intervention not indicated	Symptomatic but non-displaced; immobilization indicated	Severe symptoms; displaced or open wound with bone exposure; disabling; operative intervention indicated	Life-threatening consequences; urgent intervention indicated	Death
Definition: A finding of traumatic injury to the bone in which the continuity of the bone is broken.					
Gastric anastomotic leak	Asymptomatic diagnostic observations only; intervention not indicated	Symptomatic; medical intervention indicated	Severe symptoms; radiologic, endoscopic or elective operative intervention indicated	Life-threatening consequences; urgent operative intervention indicated	Death
Definition: A finding of leakage due to breakdown of a gastric anastomosis (surgical connection of two separate anatomic structures).					
Gastrointestinal anastomotic leak	Asymptomatic diagnostic observations only; intervention not indicated	Symptomatic; medical intervention indicated	Severe symptoms; radiologic, endoscopic or elective operative intervention indicated	Life-threatening consequences; urgent operative intervention indicated	Death
Definition: A finding of leakage due to breakdown of a gastrointestinal anastomosis (surgical connection of two separate anatomic structures).					
Gastrointestinal stoma necrosis	-	Superficial necrosis; intervention not indicated	Severe symptoms; hospitalization or elective operative intervention indicated	Life-threatening consequences; urgent intervention indicated	Death
Definition: A finding of a necrotic process occurring in the gastrointestinal tract stoma.					

Injury, poisoning and procedural complications

	Grade				
**Adverse Event**	**1**	**2**	**3**	**4**	**5**
Hip fracture	-	Hairline fracture; mild pain; limiting instrumental ADL; non-surgical intervention indicated	Severe pain; hospitalization or intervention indicated for pain control (e.g., traction); operative intervention indicated	Life-threatening consequences; symptoms associated with neurovascular compromise	-
Definition: A finding of traumatic injury to the hip in which the continuity of either the femoral head, femoral neck, intertrochanteric or subtrochanteric regions is broken.					
Injury to carotid artery	-	-	Severe symptoms; limiting self care ADL (e.g., transient cerebral ischemia); repair or revision indicated	Life-threatening consequences; urgent intervention indicated	Death
Definition: A finding of damage to the carotid artery.					
Injury to inferior vena cava	-	-	-	Life-threatening consequences; urgent intervention indicated	Death
Definition: A finding of damage to the inferior vena cava.					
Injury to jugular vein	-	-	Symptomatic limiting self care ADL; disabling; repair or revision indicated	Life-threatening consequences; urgent intervention indicated	Death
Definition: A finding of damage to the jugular vein.					

Injury, poisoning and procedural complications

	Grade				
**Adverse Event**	**1**	**2**	**3**	**4**	**5**
Injury to superior vena cava	Asymptomatic diagnostic finding; intervention not indicated	Symptomatic; repair or revision not indicated	Severe symptoms; limiting self care ADL; disabling; repair or revision indicated	Life-threatening consequences; evidence of end organ damage; urgent operative intervention indicated	Death
Definition: A finding of damage to the superior vena cava.					
Intestinal stoma leak	Asymptomatic diagnostic observations only; intervention not indicated	Symptomatic; medical intervention indicated	Severe symptoms; radiologic, endoscopic or elective operative intervention indicated	Life-threatening consequences; urgent operative intervention indicated	Death
Definition: A finding of leakage of contents from an intestinal stoma (surgically created opening on the surface of the body).					
Intestinal stoma obstruction	-	Self-limited; intervention not indicated	Severe symptoms; IV fluids, tube feeding, or TPN indicated >=24 hrs; elective operative intervention indicated	Life-threatening consequences; urgent operative intervention indicated	Death
Definition: A finding of blockage of the normal flow of the contents of the intestinal stoma.					
Intestinal stoma site bleeding	Minimal bleeding identified on clinical exam; intervention not indicated	Moderate bleeding; medical intervention indicated	Severe bleeding; transfusion indicated; radiologic or endoscopic intervention indicated	Life-threatening consequences; urgent intervention indicated	Death
Definition: A finding of blood leakage from the intestinal stoma.					

Injury, poisoning and procedural complications

	Grade				
**Adverse Event**	**1**	**2**	**3**	**4**	**5**
Intraoperative arterial injury	Primary repair of injured organ/structure indicated	Partial resection of injured organ/structure indicated	Complete resection or reconstruction of injured organ/structure indicated; disabling	Life-threatening consequences; urgent intervention indicated	Death
Definition: A finding of damage to an artery during a surgical procedure.					
Intraoperative breast injury	Primary repair of injured organ/structure indicated	Partial resection of injured organ/structure indicated	Complete resection or reconstruction of injured organ/structure indicated; disabling	Life-threatening consequences; urgent intervention indicated	Death
Definition: A finding of damage to the breast parenchyma during a surgical procedure.					
Intraoperative cardiac injury	-	-	Primary repair of injured organ/structure indicated	Life-threatening consequences; urgent intervention indicated	Death
Definition: A finding of damage to the heart during a surgical procedure.					
Intraoperative ear injury	Primary repair of injured organ/structure indicated	Partial resection of injured organ/structure indicated	Complete resection of injured organ/structure indicated; disabling (e.g., impaired hearing; impaired balance)	Life-threatening consequences; urgent intervention indicated	Death
Definition: A finding of damage to the ear during a surgical procedure.					
Intraoperative endocrine injury	Primary repair of injured organ/structure indicated	Partial resection of injured organ/structure indicated	Complete resection or reconstruction of injured organ/structure indicated; disabling	Life-threatening consequences; urgent intervention indicated	Death

Injury, poisoning and procedural complications

	Grade				
**Adverse Event**	**1**	**2**	**3**	**4**	**5**
Definition: A finding of damage to the endocrine gland during a surgical procedure.					
Intraoperative gastrointestinal injury	Primary repair of injured organ/structure indicated	Partial resection of injured organ/structure indicated	Complete resection or reconstruction of injured organ/structure indicated; disabling	Life-threatening consequences; urgent intervention indicated	Death
Definition: A finding of damage to the gastrointestinal system during a surgical procedure.					
Intraoperative head and neck injury	Primary repair of injured organ/structure indicated	Partial resection of injured organ/structure indicated	Complete resection or reconstruction of injured organ/structure indicated; disabling	Life-threatening consequences; urgent intervention indicated	Death
Definition: A finding of damage to the head and neck during a surgical procedure.					
Intraoperative hemorrhage	-	-	Postoperative radiologic, endoscopic, or operative intervention indicated	Life-threatening consequences; urgent intervention indicated	Death
Definition: A finding of uncontrolled bleeding during a surgical procedure.					
Intraoperative hepatobiliary injury	Primary repair of injured organ/structure indicated	Partial resection of injured organ/structure indicated	Complete resection or reconstruction of injured organ/structure indicated; disabling	Life-threatening consequences; urgent intervention indicated	Death
Definition: A finding of damage to the hepatic parenchyma and/or biliary tract during a surgical procedure.					

Injury, poisoning and procedural complications

	Grade				
**Adverse Event**	**1**	**2**	**3**	**4**	**5**
Intraoperative musculoskeletal injury	Primary repair of injured organ/structure indicated	Partial resection of injured organ/structure indicated	Complete resection or reconstruction of injured organ/structure indicated; disabling	Life-threatening consequences; urgent intervention indicated	Death
Definition: A finding of damage to the musculoskeletal system during a surgical procedure.					
Intraoperative neurological injury	Primary repair of injured organ/structure indicated	Partial resection of injured organ/structure indicated	Complete resection or reconstruction of injured organ/structure indicated; disabling	Life-threatening consequences; urgent intervention indicated	Death
Definition: A finding of damage to the nervous system during a surgical procedure.					
Intraoperative ocular injury	Primary repair of injured organ/structure indicated	Partial resection of injured organ/structure indicated	Complete resection or reconstruction of injured organ/structure indicated; disabling	Life-threatening consequences; urgent intervention indicated	Death
Definition: A finding of damage to the eye during a surgical procedure.					
Intraoperative renal injury	Primary repair of injured organ/structure indicated	Partial resection of injured organ/structure indicated	Complete resection or reconstruction of injured organ/structure indicated; disabling	Life-threatening consequences; urgent intervention indicated	Death
Definition: A finding of damage to the kidney during a surgical procedure.					

Injury, poisoning and procedural complications

	Grade				
**Adverse Event**	**1**	**2**	**3**	**4**	**5**
Intraoperative reproductive tract injury	Primary repair of injured organ/structure indicated	Partial resection of injured organ/structure indicated	Complete resection or reconstruction of injured organ/structure indicated; disabling	Life-threatening consequences; urgent intervention indicated	Death
Definition: A finding of damage to the reproductive organs during a surgical procedure.					
Intraoperative respiratory injury	Primary repair of injured organ/structure indicated	Partial resection of injured organ/structure indicated	Complete resection or reconstruction of injured organ/structure indicated; disabling	Life-threatening consequences; urgent intervention indicated	Death
Definition: A finding of damage to the respiratory system during a surgical procedure.					
Intraoperative skin injury	Primary repair of injured organ/structure indicated	Partial resection of injured organ/structure indicated	Complete resection or reconstruction of injured organ/structure indicated; disabling	Life-threatening consequences; urgent intervention indicated	Death
Definition: A finding of damage to the skin during a surgical procedure.					
Intraoperative splenic injury	-	Primary repair of injured organ/structure indicated	Resection or reconstruction of injured organ/structure indicated; disabling	Life-threatening consequences; urgent intervention indicated	Death
Definition: A finding of damage to the spleen during a surgical procedure.					
Intraoperative urinary injury	Primary repair of injured organ/structure indicated	Partial resection of injured organ/structure indicated	Complete resection or reconstruction of injured organ/structure indicated; disabling	Life-threatening consequences; urgent intervention indicated	Death

Injury, poisoning and procedural complications

	Grade				
**Adverse Event**	**1**	**2**	**3**	**4**	**5**
Definition: A finding of damage to the urinary system during a surgical procedure.					
Intraoperative venous injury	Primary repair of injured organ/structure indicated	Partial resection of injured organ/structure indicated	Complete resection or reconstruction of injured organ/structure indicated; disabling	Life-threatening consequences; urgent intervention indicated	Death
Definition: A finding of damage to a vein during a surgical procedure.					
Kidney anastomotic leak	Asymptomatic diagnostic observations only; intervention not indicated	Symptomatic; medical intervention indicated	Severe symptoms; radiologic, endoscopic or elective operative intervention indicated	Life-threatening consequences; urgent operative intervention indicated	Death
Definition: A finding of leakage of urine due to breakdown of a kidney anastomosis (surgical connection of two separate anatomic structures).					
Large intestinal anastomotic leak	Asymptomatic diagnostic observations only; intervention not indicated	Symptomatic; medical intervention indicated	Severe symptoms; radiologic, endoscopic or elective operative intervention indicated	Life-threatening consequences; urgent operative intervention indicated	Death
Definition: A finding of leakage due to breakdown of an anastomosis (surgical connection of two separate anatomic structures) in the large intestine.					
Pancreatic anastomotic leak	Asymptomatic diagnostic observations only; intervention not indicated	Symptomatic; medical intervention indicated	Severe symptoms; radiologic, endoscopic or elective operative intervention indicated	Life-threatening consequences; urgent operative intervention indicated	Death
Definition: A finding of leakage due to breakdown of a pancreatic anastomosis (surgical connection of two separate anatomic structures).					

Injury, poisoning and procedural complications

	Grade				
**Adverse Event**	**1**	**2**	**3**	**4**	**5**
Pharyngeal anastomotic leak	Asymptomatic diagnostic observations only; intervention not indicated	Symptomatic; medical intervention indicated	Severe symptoms; radiologic, endoscopic or elective operative intervention indicated	Life-threatening consequences; urgent operative intervention indicated	Death
Definition: A finding of leakage due to breakdown of a pharyngeal anastomosis (surgical connection of two separate anatomic structures).					
Postoperative hemorrhage	Minimal bleeding identified on clinical exam; intervention not indicated	Moderate bleeding; radiologic, endoscopic, or operative intervention indicated	Transfusion indicated of >=2 units (10 cc/kg for pediatrics) pRBCs beyond protocol specification; urgent radiologic, endoscopic, or operative intervention indicated	Life-threatening consequences; urgent intervention indicated	Death
Definition: A finding of bleeding occurring after a surgical procedure.					
Postoperative thoracic procedure complication	-	Extubated within 24 - 72 hrs postoperatively	Extubated >72 hrs postoperatively, but before tracheostomy indicated	Life-threatening airway compromise; urgent intervention indicated (e.g., tracheotomy or intubation)	Death
Definition: A finding of a previously undocumented problem that occurs after a thoracic procedure.					
Prolapse of intestinal stoma	Asymptomatic; reducible	Recurrent after manual reduction; local irritation or stool leakage; difficulty to fit appliance; limiting instrumental ADL	Severe symptoms; elective operative intervention indicated; limiting self care ADL	Life-threatening consequences; urgent operative intervention indicated	Death

Injury, poisoning and procedural complications

	Grade				
**Adverse Event**	**1**	**2**	**3**	**4**	**5**
Definition: A finding of protrusion of the intestinal stoma (surgically created opening on the surface of the body) above the abdominal surface.					
Prolapse of urostomy	Asymptomatic; clinical or diagnostic observations only; intervention not indicated	Local care or maintenance; minor revision indicated	Dysfunctional stoma; elective operative intervention or major stomal revision indicated	Life-threatening consequences; urgent intervention indicated	Death
Definition: A finding of displacement of the urostomy.					
Radiation recall reaction (dermatologic)	Faint erythema or dry desquamation	Moderate to brisk erythema; patchy moist desquamation, mostly confined to skin folds and creases; moderate edema	Moist desquamation in areas other than skin folds and creases; bleeding induced by minor trauma or abrasion	Life-threatening consequences; skin necrosis or ulceration of full thickness dermis; spontaneous bleeding from involved site; skin graft indicated	Death
Definition: A finding of acute skin inflammatory reaction caused by drugs, especially chemotherapeutic agents, for weeks or months following radiotherapy. The inflammatory reaction is confined to the previously irradiated skin and the symptoms disappear after the removal of the pharmaceutical agent.					
Rectal anastomotic leak	Asymptomatic diagnostic observations only; intervention not indicated	Symptomatic; medical intervention indicated	Severe symptoms; radiologic, endoscopic or elective operative intervention indicated	Life-threatening consequences; urgent operative intervention indicated	Death
Definition: A finding of leakage due to breakdown of a rectal anastomosis (surgical connection of two separate anatomic structures).					
Seroma	Asymptomatic; clinical or diagnostic observations only; intervention not indicated	Symptomatic; simple aspiration indicated	Symptomatic, elective radiologic or operative intervention indicated	-	-
Definition: A finding of tumor-like collection of serum in the tissues.					

Injury, poisoning and procedural complications

	Grade				
**Adverse Event**	**1**	**2**	**3**	**4**	**5**
Small intestinal anastomotic leak	Asymptomatic diagnostic observations only; intervention not indicated	Symptomatic; medical intervention indicated	Severe symptoms; radiologic, endoscopic or elective operative intervention indicated	Life-threatening consequences; urgent operative intervention indicated	Death
Definition: A finding of leakage due to breakdown of an anastomosis (surgical connection of two separate anatomic structures) in the small bowel.					
Spermatic cord anastomotic leak	Asymptomatic diagnostic observations only; intervention not indicated	Symptomatic; medical intervention indicated	Severe symptoms; radiologic, endoscopic or elective operative intervention indicated	Life-threatening consequences; urgent operative intervention indicated	Death
Definition: A finding of leakage due to breakdown of a spermatic cord anastomosis (surgical connection of two separate anatomic structures).					
Spinal fracture	Mild back pain; nonprescription analgesics indicated	Moderate back pain; prescription analgesics indicated; limiting instrumental ADL	Severe back pain; hospitalization or intervention indicated for pain control (e.g., vertebroplasty); limiting self care ADL; disability	Life-threatening consequences; symptoms associated with neurovascular compromise	Death
Definition: A finding of traumatic injury to the spine in which the continuity of a vertebral bone is broken.					
Stenosis of gastrointestinal stoma	-	Symptomatic; IV fluids indicated <24 hrs; manual dilation at bedside	Severely altered GI function; tube feeding, TPN or hospitalization indicated; elective operative intervention indicated	Life-threatening consequences; urgent operative intervention indicated	Death
Definition: A finding of narrowing of the gastrointestinal stoma (surgically created opening on the surface of the body).					

Injury, poisoning and procedural complications

	Grade				
**Adverse Event**	**1**	**2**	**3**	**4**	**5**
Stomal ulcer	Asymptomatic; clinical or diagnostic observations only; intervention not indicated	Symptomatic; medical intervention indicated	Severe symptoms; elective operative intervention indicated	-	-
Definition: A disorder characterized by a circumscribed, inflammatory and necrotic erosive lesion on the jejunal mucosal surface close to the anastomosis site following a gastroenterostomy procedure.					
Tracheal hemorrhage	Minimal bleeding identified on clinical or diagnostic exam; intervention not indicated	Moderate bleeding; medical intervention indicated	Severe bleeding; transfusion indicated; radiologic or endoscopic intervention indicated	Life-threatening consequences; urgent intervention indicated	Death
Definition: A finding of bleeding from the trachea.					
Tracheal obstruction	Partial asymptomatic obstruction on examination (e.g., visual, radiologic or endoscopic)	Symptomatic (e.g., noisy airway breathing), no respiratory distress; medical intervention indicated (e.g., steroids); limiting instrumental ADL	Stridor; radiologic or endoscopic intervention indicated (e.g., stent, laser); limiting self care ADL	Life-threatening airway compromise; urgent intervention indicated (e.g., tracheotomy or intubation)	Death
Definition: A finding of blockage of the lumen of the trachea.					
Tracheostomy site bleeding	Minimal bleeding identified on clinical exam; intervention not indicated	Moderate bleeding; medical intervention indicated	Severe bleeding; transfusion indicated; radiologic or endoscopic intervention indicated	Life-threatening consequences; urgent intervention indicated	Death
Definition: A finding of blood leakage from the tracheostomy site.					

Injury, poisoning and procedural complications

	Grade				
**Adverse Event**	**1**	**2**	**3**	**4**	**5**
Ureteric anastomotic leak	Asymptomatic diagnostic observations only; intervention not indicated	Symptomatic; medical intervention indicated	Severe symptoms; radiologic, endoscopic or elective operative intervention indicated	Life-threatening consequences; urgent operative intervention indicated	Death
Definition: A finding of leakage due to breakdown of a ureteral anastomosis (surgical connection of two separate anatomic structures).					
Urethral anastomotic leak	Asymptomatic diagnostic observations only; intervention not indicated	Symptomatic; medical intervention indicated	Severe symptoms; radiologic, endoscopic or elective operative intervention indicated	Life-threatening consequences; urgent operative intervention indicated	Death
Definition: A finding of leakage due to breakdown of a urethral anastomosis (surgical connection of two separate anatomic structures).					
Urostomy leak	Asymptomatic diagnostic observations only; intervention not indicated	Symptomatic; medical intervention indicated	Severe symptoms; radiologic, endoscopic or elective operative intervention indicated	Life-threatening consequences; urgent operative intervention indicated	Death
Definition: A finding of leakage of contents from a urostomy.					
Urostomy obstruction	Asymptomatic diagnostic observations only; intervention not indicated	Symptomatic; dilation or endoscopic repair or stent placement indicated	Altered organ function (e.g., sepsis or hydronephrosis, or renal dysfunction); elective operative intervention indicated	Life-threatening consequences; organ failure; urgent operative intervention indicated	Death
Definition: A finding of blockage of the urostomy.					

Injury, poisoning and procedural complications					
	Grade				
**Adverse Event**	**1**	**2**	**3**	**4**	**5**
Urostomy site bleeding	Minimal bleeding identified on clinical exam; intervention not indicated	Moderate bleeding; medical intervention indicated	Severe bleeding; transfusion indicated; radiologic or endoscopic intervention indicated	Life-threatening consequences; urgent intervention indicated	Death
Definition: A finding of bleeding from the urostomy site.					
Urostomy stenosis	-	Symptomatic but no hydronephrosis, no sepsis or no renal dysfunction; dilation or endoscopic repair or stent placement indicated	Symptomatic (e.g., hydronephrosis, or renal dysfunction); elective operative intervention indicated	Life-threatening consequences; urgent operative intervention indicated	Death
Definition: A finding of narrowing of the opening of a urostomy.					
Uterine anastomotic leak	Asymptomatic diagnostic observations only; intervention not indicated	Symptomatic; medical intervention indicated	Severe symptoms; radiologic, endoscopic or elective operative intervention indicated	Life-threatening consequences; urgent operative intervention indicated	Death
Definition: A finding of leakage due to breakdown of a uterine anastomosis (surgical connection of two separate anatomic structures).					
Uterine perforation	Asymptomatic diagnostic observations only; intervention not indicated	Symptomatic and intervention not indicated	Severe symptoms; elective operative intervention indicated	Life-threatening consequences; urgent intervention indicated	Death
Definition: A disorder characterized by a rupture in the uterine wall.					

Injury, poisoning and procedural complications

	Grade				
**Adverse Event**	**1**	**2**	**3**	**4**	**5**
Vaginal anastomotic leak	Asymptomatic diagnostic observations only; intervention not indicated	Symptomatic; medical intervention indicated	Severe symptoms; radiologic, endoscopic or elective operative intervention indicated	Life-threatening consequences; urgent operative intervention indicated	Death
Definition: A finding of leakage due to breakdown of a vaginal anastomosis (surgical connection of two separate anatomic structures).					
Vas deferens anastomotic leak	Asymptomatic diagnostic observations only; intervention not indicated	Symptomatic; medical intervention indicated	Severe symptoms; radiologic, endoscopic or elective operative intervention indicated	Life-threatening consequences; urgent operative intervention indicated	Death
Definition: A finding of leakage due to breakdown of a vas deferens anastomosis (surgical connection of two separate anatomic structures).					
Vascular access complication	-	Device dislodgement, blockage, leak, or malposition; device replacement indicated	Deep vein or cardiac thrombosis; intervention indicated (e.g., anticoagulation, lysis, filter, invasive procedure)	Embolic event including pulmonary embolism or life-threatening thrombus	Death
Definition: A finding of a previously undocumented problem related to the vascular access site.					
Venous injury	Asymptomatic diagnostic finding; intervention not indicated	Symptomatic (e.g., claudication); repair or revision not indicated	Severe symptoms; limiting self care ADL; repair or revision indicated; disabling	Life-threatening consequences; evidence of end organ damage; urgent operative intervention indicated	Death
Definition: A finding of damage to a vein.					

Injury, poisoning and procedural complications

	Grade				
**Adverse Event**	**1**	**2**	**3**	**4**	**5**
Wound complication	Incisional separation of <=25% of wound, no deeper than superficial fascia	Incisional separation >25% of wound; local care indicated	Hernia without evidence of strangulation; fascial disruption/dehiscence; primary wound closure or revision by operative intervention indicated	Hernia with evidence of strangulation; major reconstruction flap, grafting, resection, or amputation indicated	Death
Definition: A finding of development of a new problem at the site of an existing wound.					
Wound dehiscence	Incisional separation of <=25% of wound, no deeper than superficial fascia	Incisional separation >25% of wound with local care; asymptomatic hernia or symptomatic hernia without evidence of strangulation	Fascial disruption or dehiscence without evisceration; primary wound closure or revision by operative intervention indicated	Life-threatening consequences; symptomatic hernia with evidence of strangulation; fascial disruption with evisceration; major reconstruction flap, grafting, resection, or amputation indicated	Death
Definition: A finding of separation of the approximated margins of a surgical wound.					
Wrist fracture	Mild; non-surgical intervention indicated	Limiting instrumental ADL; operative intervention indicated	Limiting self care ADL; elective surgery indicated	-	-
Definition: A finding of traumatic injury to the wrist joint in which the continuity of a wrist bone is broken.					

Injury, poisoning and procedural complications

	Grade				
**Adverse Event**	**1**	**2**	**3**	**4**	**5**
Injury, poisoning and procedural complications - Other, specify	Asymptomatic or mild symptoms; clinical or diagnostic observations only; intervention not indicated	Moderate; minimal, local or noninvasive intervention indicated; limiting age-appropriate instrumental ADL	Severe or medically significant but not immediately life-threatening; hospitalization or prolongation of existing hospitalization indicated; disabling; limiting self care ADL	Life-threatening consequences; urgent intervention indicated	Death

Investigations

	Grade				
**Adverse Event**	**1**	**2**	**3**	**4**	**5**
Activated partial thromboplastin time prolonged	>ULN - 1.5 x ULN	>1.5 - 2.5 x ULN	>2.5 x ULN; hemorrhage	-	-
Definition: An abnormal laboratory test result in which the partial thromboplastin time is found to be greater than the control value. As a possible indicator of coagulopathy, a prolonged partial thromboplastin time (PTT) may occur in a variety of diseases and disorders, both primary and related to treatment.					
Alanine aminotransferase increased	>ULN - 3.0 x ULN	>3.0 - 5.0 x ULN	>5.0 - 20.0 x ULN	>20.0 x ULN	-
Definition: A finding based on laboratory test results that indicate an increase in the level of alanine aminotransferase (ALT or SGPT) in the blood specimen.					
Alkaline phosphatase increased	>ULN - 2.5 x ULN	>2.5 - 5.0 x ULN	>5.0 - 20.0 x ULN	>20.0 x ULN	-
Definition: A finding based on laboratory test results that indicate an increase in the level of alkaline phosphatase in a blood specimen.					
Aspartate aminotransferase increased	>ULN - 3.0 x ULN	>3.0 - 5.0 x ULN	>5.0 - 20.0 x ULN	>20.0 x ULN	-
Definition: A finding based on laboratory test results that indicate an increase in the level of aspartate aminotransferase (AST or SGOT) in a blood specimen.					
Blood antidiuretic hormone abnormal	Asymptomatic; clinical or diagnostic observations only; intervention not indicated	Symptomatic; medical intervention indicated	Hospitalization indicated	-	-
Definition: A finding based on laboratory test results that indicate abnormal levels of antidiuretic hormone in the blood specimen.					
Blood bilirubin increased	>ULN - 1.5 x ULN	>1.5 - 3.0 x ULN	>3.0 - 10.0 x ULN	>10.0 x ULN	-
Definition: A finding based on laboratory test results that indicate an abnormally high level of bilirubin in the blood. Excess bilirubin is associated with jaundice.					
Blood corticotrophin decreased	Asymptomatic; clinical or diagnostic observations only; intervention not indicated	Symptomatic; medical intervention indicated	Hospitalization indicated	-	-

Investigations

	Grade				
**Adverse Event**	**1**	**2**	**3**	**4**	**5**
Definition: A finding based on laboratory test results that indicate an decrease in levels of corticotrophin in a blood specimen.					
Blood gonadotrophin abnormal	Asymptomatic; clinical or diagnostic observations only; intervention not indicated	Symptomatic; medical intervention indicated; limiting instrumental ADL	Severe symptoms; limiting self care ADL	-	-
Definition: A finding based on laboratory test results that indicate abnormal levels of gonadotrophin hormone in a blood specimen.					
Blood prolactin abnormal	Asymptomatic; clinical or diagnostic observations only; intervention not indicated	Moderate symptoms; limiting instrumental ADL	-	-	-
Definition: A finding based on laboratory test results that indicate abnormal levels of prolactin hormone in a blood specimen.					
Carbon monoxide diffusing capacity decreased	3 - 5 units below LLN; for follow-up, a decrease of 3 - 5 units (ml/min/mm Hg) below the baseline value	6 - 8 units below LLN; for follow-up, an asymptomatic decrease of >5 - 8 units (ml/min/mm Hg) below the baseline value	Asymptomatic decrease of >8 units drop; >5 units drop along with the presence of pulmonary symptoms (e.g. , >Grade 2 hypoxia or >Grade 2 or higher dyspnea)	-	-
Definition: A finding based on lung function test results that indicate a decrease in the lung capacity to absorb carbon monoxide.					
Cardiac troponin I increased	Levels above the upper limit of normal and below the level of myocardial infarction as defined by the manufacturer	-	Levels consistent with myocardial infarction as defined by the manufacturer	-	-
Definition: A laboratory test result which indicates increased levels of cardiac troponin I in a biological specimen.					

Investigations

	Grade				
**Adverse Event**	**1**	**2**	**3**	**4**	**5**
Cardiac troponin T increased	Levels above the upper limit of normal and below the level of myocardial infarction as defined by the manufacturer	-	Levels consistent with myocardial infarction as defined by the manufacturer	-	-
Definition: A laboratory test result which indicates increased levels of cardiac troponin T in a biological specimen.					
CD4 lymphocytes decreased	<LLN - 500/mm3; <LLN - 0.5 x 10e9 /L	<500 - 200/mm3; <0.5 - 0.2 x 10e9 /L	<200 - 50/mm3; <0.2 x 0.05 - 10e9 /L	<50/mm3; <0.05 x 10e9 /L	-
Definition: A finding based on laboratory test results that indicate an decrease in levels of CD4 lymphocytes in a blood specimen.					
Cholesterol high	>ULN - 300 mg/dL; >ULN - 7.75 mmol/L	>300 - 400 mg/dL; >7.75 - 10.34 mmol/L	>400 - 500 mg/dL; >10.34 - 12.92 mmol/L	>500 mg/dL; >12.92 mmol/L	-
Definition: A finding based on laboratory test results that indicate higher than normal levels of cholesterol in a blood specimen.					
CPK increased	>ULN - 2.5 x ULN	>2.5 x ULN - 5 x ULN	>5 x ULN - 10 x ULN	>10 x ULN	-
Definition: A finding based on laboratory test results that indicate an increase in levels of creatine phosphokinase in a blood specimen.					
Creatinine increased	>1 - 1.5 x baseline; >ULN - 1.5 x ULN	>1.5 - 3.0 x baseline; >1.5 - 3.0 x ULN	>3.0 baseline; >3.0 - 6.0 x ULN	>6.0 x ULN	-
Definition: A finding based on laboratory test results that indicate increased levels of creatinine in a biological specimen.					
Ejection fraction decreased	-	Resting ejection fraction (EF) 50 - 40%; 10 - 19% drop from baseline	Resting ejection fraction (EF) 39 - 20%; >20% drop from baseline	Resting ejection fraction (EF) <20%	-
Definition: The percentage computed when the amount of blood ejected during a ventricular contraction of the heart is compared to the amount that was present prior to the contraction.					

Investigations

	Grade				
**Adverse Event**	**1**	**2**	**3**	**4**	**5**
Electrocardiogram QT corrected interval prolonged	QTc 450 - 480 ms	QTc 481 - 500 ms	QTc >= 501 ms on at least two separate ECGs	QTc >= 501 or >60 ms change from baseline and Torsade de pointes or polymorphic ventricular tachycardia or signs/symptoms of serious arrhythmia	-
Definition: A finding of a cardiac dysrhythmia characterized by an abnormally long corrected QT interval.					
Fibrinogen decreased	<1.0 - 0.75 x LLN or <25% decrease from baseline	<0.75 - 0.5 x LLN or 25 - <50% decrease from baseline	<0.5 - 0.25 x LLN or 50 - <75% decrease from baseline	<0.25 x LLN or 75% decrease from baseline or absolute value <50 mg/dL	-
Definition: A finding based on laboratory test results that indicate an decrease in levels of fibrinogen in a blood specimen.					
Forced expiratory volume decreased	FEV1% (percentages of observed FEV1 and FVC related to their respective predicted values) 99 - 70% predicted	FEV1 60 - 69%	50 - 59%	<= 49%	-
Definition: A finding based on test results that indicate a relative decrease in the fraction of the forced vital capacity that is exhaled in a specific number of seconds.					
GGT increased	>ULN - 2.5 x ULN	>2.5 - 5.0 x ULN	>5.0 - 20.0 x ULN	>20.0 x ULN	-
Definition: A finding based on laboratory test results that indicate higher than normal levels of the enzyme gamma-glutamyltransferase in the blood specimen. GGT (gamma-glutamyltransferase) catalyzes the transfer of a gamma glutamyl group from a gamma glutamyl peptide to another peptide, amino acids or water.					

Investigations

	Grade				
**Adverse Event**	**1**	**2**	**3**	**4**	**5**
Growth hormone abnormal	Asymptomatic; clinical or diagnostic observations only; intervention not indicated	Symptomatic; medical intervention indicated; limiting instrumental ADL	-	-	-
Definition: A finding based on laboratory test results that indicate abnormal levels of growth hormone in a biological specimen.					
Haptoglobin decreased	<LLN	-	-	-	-
Definition: A finding based on laboratory test results that indicate an decrease in levels of haptoglobin in a blood specimen.					
Hemoglobin increased	Increase in >0 - 2 gm/dL above ULN or above baseline if baseline is above ULN	Increase in >2 - 4 gm/dL above ULN or above baseline if baseline is above ULN	Increase in >4 gm/dL above ULN or above baseline if baseline is above ULN	-	-
Definition: A finding based on laboratory test results that indicate increased levels of hemoglobin in a biological specimen.					
INR increased	>1 - 1.5 x ULN; >1 - 1.5 times above baseline if on anticoagulation	>1.5 - 2.5 x ULN; >1.5 - 2.5 times above baseline if on anticoagulation	>2.5 x ULN; >2.5 times above baseline if on anticoagulation	-	-
Definition: A finding based on laboratory test results that indicate an increase in the ratio of the patient's prothrombin time to a control sample in the blood.					
Lipase increased	>ULN - 1.5 x ULN	>1.5 - 2.0 x ULN	>2.0 - 5.0 x ULN	>5.0 x ULN	-
Definition: A finding based on laboratory test results that indicate an increase in the level of lipase in a biological specimen.					
Lymphocyte count decreased	<LLN - 800/mm3; <LLN - 0.8 x 10e9 /L	<800 - 500/mm3; <0.8 - 0.5 x 10e9 /L	<500 - 200/mm3; <0.5 - 0.2 x 10e9 /L	<200/mm3; <0.2 x 10e9 /L	-
Definition: A finding based on laboratory test results that indicate a decrease in number of lymphocytes in a blood specimen.					
Lymphocyte count increased	-	>4000/mm3 - 20,000/mm3	>20,000/mm3	-	-
Definition: A finding based on laboratory test results that indicate an abnormal increase in the number of lymphocytes in the blood, effusions or bone marrow.					

Investigations

	Grade				
**Adverse Event**	**1**	**2**	**3**	**4**	**5**
Neutrophil count decreased	<LLN - 1500/mm3; <LLN - 1.5 x 10e9 /L	<1500 - 1000/mm3; <1.5 - 1.0 x 10e9 /L	<1000 - 500/mm3; <1.0 - 0.5 x 10e9 /L	<500/mm3; <0.5 x 10e9 /L	-
Definition: A finding based on laboratory test results that indicate a decrease in number of neutrophils in a blood specimen.					
Pancreatic enzymes decreased	<LLN and asymptomatic	Increase in stool frequency, bulk, or odor; steatorrhea	Sequelae of absorption deficiency	-	-
Definition: A finding based on laboratory test results that indicate an decrease in levels of pancreatic enzymes in a biological specimen.					
Platelet count decreased	<LLN - 75,000/mm3; <LLN - 75.0 x 10e9 /L	<75,000 - 50,000/mm3; <75.0 - 50.0 x 10e9 /L	<50,000 - 25,000/mm3; <50.0 - 25.0 x 10e9 /L	<25,000/mm3; <25.0 x 10e9 /L	-
Definition: A finding based on laboratory test results that indicate a decrease in number of platelets in a blood specimen.					
Serum amylase increased	>ULN - 1.5 x ULN	>1.5 - 2.0 x ULN	>2.0 - 5.0 x ULN	>5.0 x ULN	-
Definition: A finding based on laboratory test results that indicate an increase in the levels of amylase in a serum specimen.					
Urine output decreased	-	-	Oliguria (<80 ml in 8 hr)	Anuria (<240 ml in 24 hr)	-
Definition: A finding based on test results that indicate urine production is less relative to previous output.					
Vital capacity abnormal	90 - 75% of predicted value	<75 - 50% of predicted value; limiting instrumental ADL	<50% of predicted value; limiting self care ADL	-	-
Definition: A finding based on pulmonary function test results that indicate an abnormal vital capacity (amount of exhaled after a maximum inhalation) when compared to the predicted value.					
Weight gain	5 - <10% from baseline	10 - <20% from baseline	>=20% from baseline	-	-
Definition: A finding characterized by an increase in overall body weight; for pediatrics, greater than the baseline growth curve.					
Weight loss	5 to <10% from baseline; intervention not indicated	10 - <20% from baseline; nutritional support indicated	>=20% from baseline; tube feeding or TPN indicated	-	-

Investigations

	Grade				
**Adverse Event**	**1**	**2**	**3**	**4**	**5**
Definition: A finding characterized by a decrease in overall body weight; for pediatrics, less than the baseline growth curve.					
White blood cell decreased	<LLN - 3000/mm3; <LLN - 3.0 x 10e9 /L	<3000 - 2000/mm3; <3.0 - 2.0 x 10e9 /L	<2000 - 1000/mm3; <2.0 - 1.0 x 10e9 /L	<1000/mm3; <1.0 x 10e9 /L	-
Definition: A finding based on laboratory test results that indicate an decrease in number of white blood cells in a blood specimen.					
Investigations - Other, specify	Asymptomatic or mild symptoms; clinical or diagnostic observations only; intervention not indicated	Moderate; minimal, local or noninvasive intervention indicated; limiting age-appropriate instrumental ADL	Severe or medically significant but not immediately life-threatening; hospitalization or prolongation of existing hospitalization indicated; disabling; limiting self care ADL	Life-threatening consequences; urgent intervention indicated	Death

Metabolism and nutrition disorders

	Grade				
**Adverse Event**	**1**	**2**	**3**	**4**	**5**
Acidosis	pH <normal, but >=7.3	-	pH <7.3	Life-threatening consequences	Death
Definition: A disorder characterized by abnormally high acidity (high hydrogen-ion concentration) of the blood and other body tissues.					
Alcohol intolerance	-	Present	Severe symptoms; limiting self care ADL	Life-threatening consequences; urgent intervention indicated	Death
Definition: A disorder characterized by an increase in sensitivity to the adverse effects of alcohol, which can include nasal congestion, skin flushes, heart dysrhythmias, nausea, vomiting, indigestion and headaches.					
Alkalosis	pH >normal, but <=7.5	-	pH >7.5	Life-threatening consequences	Death
Definition: A disorder characterized by abnormally high alkalinity (low hydrogen-ion concentration) of the blood and other body tissues.					
Anorexia	Loss of appetite without alteration in eating habits	Oral intake altered without significant weight loss or malnutrition; oral nutritional supplements indicated	Associated with significant weight loss or malnutrition (e.g., inadequate oral caloric and/or fluid intake); tube feeding or TPN indicated	Life-threatening consequences; urgent intervention indicated	Death
Definition: A disorder characterized by a loss of appetite.					
Dehydration	Increased oral fluids indicated; dry mucous membranes; diminished skin turgor	IV fluids indicated <24 hrs	IV fluids or hospitalization indicated	Life-threatening consequences; urgent intervention indicated	Death
Definition: A disorder characterized by excessive loss of water from the body. It is usually caused by severe diarrhea, vomiting or diaphoresis.					

Metabolism and nutrition disorders

	Grade				
**Adverse Event**	**1**	**2**	**3**	**4**	**5**
Glucose intolerance	Asymptomatic; clinical or diagnostic observations only; intervention not indicated	Symptomatic; dietary modification or oral agent indicated	Severe symptoms; insulin indicated	Life-threatening consequences; urgent intervention indicated	Death
Definition: A disorder characterized by an inability to properly metabolize glucose.					
Hypercalcemia	Corrected serum calcium of >ULN - 11.5 mg/dL; >ULN - 2.9 mmol/L; Ionized calcium >ULN - 1.5 mmol/L	Corrected serum calcium of >11.5 - 12.5 mg/dL; >2.9 - 3.1 mmol/L; Ionized calcium >1.5 - 1.6 mmol/L; symptomatic	Corrected serum calcium of >12.5 - 13.5 mg/dL; >3.1 - 3.4 mmol/L; Ionized calcium >1.6 - 1.8 mmol/L; hospitalization indicated	Corrected serum calcium of >13.5 mg/dL; >3.4 mmol/L; Ionized calcium >1.8 mmol/L; life-threatening consequences	Death
Definition: A disorder characterized by laboratory test results that indicate an elevation in the concentration of calcium (corrected for albumin) in blood.					
Hyperglycemia	Fasting glucose value >ULN - 160 mg/dL; Fasting glucose value >ULN - 8.9 mmol/L	Fasting glucose value >160 - 250 mg/dL; Fasting glucose value >8.9 - 13.9 mmol/L	>250 - 500 mg/dL; >13.9 - 27.8 mmol/L; hospitalization indicated	>500 mg/dL; >27.8 mmol/L; life-threatening consequences	Death
Definition: A disorder characterized by laboratory test results that indicate an elevation in the concentration of blood sugar. It is usually an indication of diabetes mellitus or glucose intolerance.					
Hyperkalemia	>ULN - 5.5 mmol/L	>5.5 - 6.0 mmol/L	>6.0 - 7.0 mmol/L; hospitalization indicated	>7.0 mmol/L; life-threatening consequences	Death
Definition: A disorder characterized by laboratory test results that indicate an elevation in the concentration of potassium in the blood; associated with kidney failure or sometimes with the use of diuretic drugs.					
Hypermagnesemia	>ULN - 3.0 mg/dL; >ULN - 1.23 mmol/L	-	>3.0 - 8.0 mg/dL; >1.23 - 3.30 mmol/L	>8.0 mg/dL; >3.30 mmol/L; life-threatening consequences	Death
Definition: A disorder characterized by laboratory test results that indicate an elevation in the concentration of magnesium in the blood.					

Metabolism and nutrition disorders

	Grade				
**Adverse Event**	**1**	**2**	**3**	**4**	**5**
Hypernatremia	>ULN - 150 mmol/L	>150 - 155 mmol/L	>155 - 160 mmol/L; hospitalization indicated	>160 mmol/L; life-threatening consequences	Death
Definition: A disorder characterized by laboratory test results that indicate an elevation in the concentration of sodium in the blood.					
Hypertriglyceridemia	150 mg/dL - 300 mg/dL; 1.71 mmol/L - 3.42 mmol/L	>300 mg/dL - 500 mg/dL; >3.42 mmol/L - 5.7 mmol/L	>500 mg/dL - 1000 mg/dL; >5.7 mmol/L - 11.4 mmol/L	>1000 mg/dL; >11.4 mmol/L; life-threatening consequences	Death
Definition: A disorder characterized by laboratory test results that indicate an elevation in the concentration of triglyceride concentration in the blood.					
Hyperuricemia	>ULN - 10 mg/dL (0.59 mmol/L) without physiologic consequences	-	>ULN - 10 mg/dL (0.59 mmol/L) with physiologic consequences	>10 mg/dL; >0.59 mmol/L; life-threatening consequences	Death
Definition: A disorder characterized by laboratory test results that indicate an elevation in the concentration of uric acid.					
Hypoalbuminemia	<LLN - 3 g/dL; <LLN - 30 g/L	<3 - 2 g/dL; <30 - 20 g/L	<2 g/dL; <20 g/L	Life-threatening consequences; urgent intervention indicated	Death
Definition: A disorder characterized by laboratory test results that indicate a low concentration of albumin in the blood.					
Hypocalcemia	Corrected serum calcium of <LLN - 8.0 mg/dL; <LLN - 2.0 mmol/L; Ionized calcium <LLN - 1.0 mmol/L	Corrected serum calcium of <8.0 - 7.0 mg/dL; <2.0 - 1.75 mmol/L; Ionized calcium <1.0 - 0.9 mmol/L; symptomatic	Corrected serum calcium of <7.0 - 6.0 mg/dL; <1.75 - 1.5 mmol/L; Ionized calcium <0.9 - 0.8 mmol/L; hospitalization indicated	Corrected serum calcium of <6.0 mg/dL; <1.5 mmol/L; Ionized calcium <0.8 mmol/L; life-threatening consequences	Death
Definition: A disorder characterized by laboratory test results that indicate a low concentration of calcium (corrected for albumin) in the blood.					

Metabolism and nutrition disorders

	Grade				
**Adverse Event**	**1**	**2**	**3**	**4**	**5**
Hypoglycemia	<LLN - 55 mg/dL; <LLN - 3.0 mmol/L	<55 - 40 mg/dL; <3.0 - 2.2 mmol/L	<40 - 30 mg/dL; <2.2 - 1.7 mmol/L	<30 mg/dL; <1.7 mmol/L; life-threatening consequences; seizures	Death
Definition: A disorder characterized by laboratory test results that indicate a low concentration of glucose in the blood.					
Hypokalemia	<LLN - 3.0 mmol/L	<LLN - 3.0 mmol/L; symptomatic; intervention indicated	<3.0 - 2.5 mmol/L; hospitalization indicated	<2.5 mmol/L; life-threatening consequences	Death
Definition: A disorder characterized by laboratory test results that indicate a low concentration of potassium in the blood.					
Hypomagnesemia	<LLN - 1.2 mg/dL; <LLN - 0.5 mmol/L	<1.2 - 0.9 mg/dL; <0.5 - 0.4 mmol/L	<0.9 - 0.7 mg/dL; <0.4 - 0.3 mmol/L	<0.7 mg/dL; <0.3 mmol/L; life-threatening consequences	Death
Definition: A disorder characterized by laboratory test results that indicate a low concentration of magnesium in the blood.					
Hyponatremia	<LLN - 130 mmol/L	-	<130 - 120 mmol/L	<120 mmol/L; life-threatening consequences	Death
Definition: A disorder characterized by laboratory test results that indicate a low concentration of sodium in the blood.					
Hypophosphatemia	<LLN - 2.5 mg/dL; <LLN - 0.8 mmol/L	<2.5 - 2.0 mg/dL; <0.8 - 0.6 mmol/L	<2.0 - 1.0 mg/dL; <0.6 - 0.3 mmol/L	<1.0 mg/dL; <0.3 mmol/L; life-threatening consequences	Death
Definition: A disorder characterized by laboratory test results that indicate a low concentration of phosphates in the blood.					
Iron overload	-	Moderate symptoms; intervention not indicated	Severe symptoms; intervention indicated	Life-threatening consequences; urgent intervention indicated	Death
Definition: A disorder characterized by accumulation of iron in the tissues.					
Obesity	-	BMI 25 - 29.9 kg/m2	BMI 30 - 39.9 kg/m2	BMI >=40 kg/m2	-

Metabolism and nutrition disorders

	Grade				
**Adverse Event**	**1**	**2**	**3**	**4**	**5**
Definition: A disorder characterized by having a high amount of body fat.					
Tumor lysis syndrome	-	-	Present	Life-threatening consequences; urgent intervention indicated	Death
Definition: A disorder characterized by metabolic abnormalities that result from a spontaneous or therapy-related cytolysis of tumor cells.					
Metabolism and nutrition disorders - Other, specify	Asymptomatic or mild symptoms; clinical or diagnostic observations only; intervention not indicated	Moderate; minimal, local or noninvasive intervention indicated; limiting age-appropriate instrumental ADL	Severe or medically significant but not immediately life-threatening; hospitalization or prolongation of existing hospitalization indicated; disabling; limiting self care ADL	Life-threatening consequences; urgent intervention indicated	Death

Musculoskeletal and connective tissue disorders

	Grade				
**Adverse Event**	**1**	**2**	**3**	**4**	**5**
Abdominal soft tissue necrosis	-	Local wound care; medical intervention indicated (e.g., dressings or topical medications)	Operative debridement or other invasive intervention indicated (e.g. tissue reconstruction, flap or grafting)	Life-threatening consequences; urgent intervention indicated	Death
Definition: A disorder characterized by a necrotic process occurring in the soft tissues of the abdominal wall.					
Arthralgia	Mild pain	Moderate pain; limiting instrumental ADL	Severe pain; limiting self care ADL	-	-
Definition: A disorder characterized by a sensation of marked discomfort in a joint.					
Arthritis	Mild pain with inflammation, erythema, or joint swelling	Moderate pain associated with signs of inflammation, erythema, or joint swelling; limiting instrumental ADL	Severe pain associated with signs of inflammation, erythema, or joint swelling; irreversible joint damage; disabling; limiting self care ADL	-	-
Definition: A disorder characterized by inflammation involving a joint.					
Avascular necrosis	Asymptomatic; clinical or diagnostic observations only; intervention not indicated	Symptomatic; limiting instrumental ADL	Severe symptoms; limiting self care ADL; elective operative intervention indicated	Life-threatening consequences; urgent intervention indicated	Death
Definition: A disorder characterized by necrotic changes in the bone tissue due to interruption of blood supply. Most often affecting the epiphysis of the long bones, the necrotic changes result in the collapse and the destruction of the bone structure.					
Back pain	Mild pain	Moderate pain; limiting instrumental ADL	Severe pain; limiting self care ADL	-	-

Musculoskeletal and connective tissue disorders					
	Grade				
**Adverse Event**	**1**	**2**	**3**	**4**	**5**
Definition: A disorder characterized by marked discomfort sensation in the back region.					
Bone pain	Mild pain	Moderate pain; limiting instrumental ADL	Severe pain; limiting self care ADL	-	-
Definition: A disorder characterized by marked discomfort sensation in the bones.					
Buttock pain	Mild pain	Moderate pain; limiting instrumental ADL	Severe pain; limiting self care ADL	-	-
Definition: A disorder characterized by marked discomfort sensation in the buttocks.					
Chest wall pain	Mild pain	Moderate pain; limiting instrumental ADL	Severe pain; limiting self care ADL	-	-
Definition: A disorder characterized by marked discomfort sensation in the chest wall region.					
Exostosis	Asymptomatic; clinical or diagnostic observations only; intervention not indicated	Symptomatic; limiting instrumental ADL	Severe symptoms; limiting self care ADL; elective operative intervention indicated	-	-
Definition: A disorder characterized by non-neoplastic overgrowth of bone.					
Fibrosis deep connective tissue	Mild induration, able to move skin parallel to plane (sliding) and perpendicular to skin (pinching up)	Moderate induration, able to slide skin, unable to pinch skin; limiting instrumental ADL	Severe induration; unable to slide or pinch skin; limiting joint or orifice movement (e.g. mouth, anus); limiting self care ADL	Generalized; associated with signs or symptoms of impaired breathing or feeding	Death
Definition: A disorder characterized by fibrotic degeneration of the deep connective tissues.					
Flank pain	Mild pain	Moderate pain; limiting instrumental ADL	Severe pain; limiting self care ADL	-	-

Musculoskeletal and connective tissue disorders

	Grade				
**Adverse Event**	**1**	**2**	**3**	**4**	**5**
Definition: A disorder characterized by marked discomfort sensation on the lateral side of the body in the region below the ribs and above the hip.					
Generalized muscle weakness	Symptomatic; weakness perceived by patient but not evident on physical exam	Symptomatic; weakness evident on physical exam; weakness limiting instrumental ADL	Weakness limiting self care ADL; disabling	-	-
Definition: A disorder characterized by a reduction in the strength of muscles in multiple anatomic sites.					
Growth suppression	Reduction in growth velocity by 10 - 29% ideally measured over the period of a year	Reduction in growth velocity by 30 - 49% ideally measured over the period of a year or 0 - 49% reduction in growth from the baseline growth curve	Reduction in growth velocity of >=50% ideally measured over the period of a year	-	-
Definition: A disorder characterized by of stature that is smaller than normal as expected for age.					
Head soft tissue necrosis	-	Local wound care; medical intervention indicated (e.g., dressings or topical medications)	Operative debridement or other invasive intervention indicated (e.g., tissue reconstruction, flap or grafting)	Life-threatening consequences; urgent intervention indicated	Death
Definition: A disorder characterized by a necrotic process occurring in the soft tissues of the head.					
Joint effusion	Asymptomatic; clinical or diagnostic observations only; intervention not indicated	Symptomatic; limiting instrumental ADL	Severe symptoms; limiting self care ADL; elective operative intervention indicated; disabling	-	-
Definition: A disorder characterized by excessive fluid in a joint, usually as a result of joint inflammation.					

Musculoskeletal and connective tissue disorders

	Grade				
**Adverse Event**	**1**	**2**	**3**	**4**	**5**
Joint range of motion decreased	<=25% loss of ROM (range of motion); decreased ROM limiting athletic activity	>25 - 50% decrease in ROM; limiting instrumental ADL	>50% decrease in ROM; limiting self care ADL; disabling	-	-
Definition: A disorder characterized by a decrease in joint flexibility of any joint.					
Joint range of motion decreased cervical spine	Mild restriction of rotation or flexion between 60 - 70 degrees	Rotation <60 degrees to right or left; <60 degrees of flexion	Ankylosed/fused over multiple segments with no C-spine rotation	-	-
Definition: A disorder characterized by a decrease in flexibility of a cervical spine joint.					
Joint range of motion decreased lumbar spine	Stiffness; difficulty bending to the floor to pick up a very light object but able to do athletic activity	Pain with range of motion (ROM) in lumbar spine; requires a reaching aid to pick up a very light object from the floor	<50% lumbar spine flexion; associated with symptoms of ankylosis or fused over multiple segments with no L-spine flexion (e.g., unable to reach to floor to pick up a very light object)	-	-
Definition: A disorder characterized by a decrease in flexibility of a lumbar spine joint.					
Kyphosis	Asymptomatic; clinical or diagnostic observations only; intervention not indicated	Moderate accentuation; limiting instrumental ADL	Severe accentuation; operative intervention indicated; limiting self care ADL	-	-
Definition: A disorder characterized by an abnormal increase in the curvature of the thoracic portion of the spine.					

Musculoskeletal and connective tissue disorders

	Grade				
**Adverse Event**	**1**	**2**	**3**	**4**	**5**
Lordosis	Asymptomatic; clinical or diagnostic observations only; intervention not indicated	Moderate accentuation; limiting instrumental ADL	Severe accentuation; operative intervention indicated; limiting self care ADL	-	-
Definition: A disorder characterized by an abnormal increase in the curvature of the lumbar portion of the spine.					
Muscle weakness left-sided	Symptomatic; perceived by patient but not evident on physical exam	Symptomatic; evident on physical exam; limiting instrumental ADL	Limiting self care ADL; disabling	-	-
Definition: A disorder characterized by a reduction in the strength of the muscles on the left side of the body.					
Muscle weakness lower limb	Symptomatic; perceived by patient but not evident on physical exam	Symptomatic; evident on physical exam; limiting instrumental ADL	Limiting self care ADL; disabling	-	-
Definition: A disorder characterized by a reduction in the strength of the lower limb muscles.					
Muscle weakness right-sided	Symptomatic; perceived by patient but not evident on physical exam	Symptomatic; evident on physical exam; limiting instrumental ADL	Limiting self care ADL; disabling	-	-
Definition: A disorder characterized by a reduction in the strength of the muscles on the right side of the body.					
Muscle weakness trunk	Symptomatic; perceived by patient but not evident on physical exam	Symptomatic; evident on physical exam; limiting instrumental ADL	Limiting self care ADL; disabling	-	-
Definition: A disorder characterized by a reduction in the strength of the trunk muscles.					

Musculoskeletal and connective tissue disorders

	Grade				
**Adverse Event**	**1**	**2**	**3**	**4**	**5**
Muscle weakness upper limb	Symptomatic; perceived by patient but not evident on physical exam	Symptomatic; evident on physical exam; limiting instrumental ADL	Limiting self care ADL; disabling	-	-
Definition: A disorder characterized by a reduction in the strength of the upper limb muscles.					
Musculoskeletal deformity	Cosmetically and functionally insignificant hypoplasia	Deformity, hypoplasia, or asymmetry able to be remediated by prosthesis (e.g., shoe insert) or covered by clothing	Significant deformity, hypoplasia, or asymmetry, unable to be remediated by prosthesis or covered by clothing; disabling	-	-
Definition: A disorder characterized by of a malformation of the musculoskeletal system.					
Myalgia	Mild pain	Moderate pain; limiting instrumental ADL	Severe pain; limiting self care ADL	-	-
Definition: A disorder characterized by marked discomfort sensation originating from a muscle or group of muscles.					
Myositis	Mild pain	Moderate pain associated with weakness; pain limiting instrumental ADL	Pain associated with severe weakness; limiting self care ADL	-	-
Definition: A disorder characterized by inflammation involving the skeletal muscles.					
Neck pain	Mild pain	Moderate pain; limiting instrumental ADL	Severe pain; limiting self care ADL	-	-
Definition: A disorder characterized by marked discomfort sensation in the neck area.					

Musculoskeletal and connective tissue disorders

	Grade				
**Adverse Event**	**1**	**2**	**3**	**4**	**5**
Neck soft tissue necrosis	-	Local wound care; medical intervention indicated (e.g., dressings or topical medications)	Operative debridement or other invasive intervention indicated (e.g., tissue reconstruction, flap or grafting)	Life-threatening consequences; urgent intervention indicated	Death
Definition: A disorder characterized by a necrotic process occurring in the soft tissues of the neck.					
Osteonecrosis of jaw	Asymptomatic; clinical or diagnostic observations only; intervention not indicated	Symptomatic; medical intervention indicated (e.g., topical agents); limiting instrumental ADL	Severe symptoms; limiting self care ADL; elective operative intervention indicated	Life-threatening consequences; urgent intervention indicated	Death
Definition: A disorder characterized by a necrotic process occurring in the bone of the mandible.					
Osteoporosis	Radiologic evidence of osteoporosis or Bone Mineral Density (BMD) t-score -1 to -2.5 (osteopenia); no loss of height or intervention indicated	BMD t-score <-2.5; loss of height <2 cm; anti-osteoporotic therapy indicated; limiting instrumental ADL	Loss of height >=2 cm; hospitalization indicated; limiting self care ADL	-	-
Definition: A disorder characterized by reduced bone mass, with a decrease in cortical thickness and in the number and size of the trabeculae of cancellous bone (but normal chemical composition), resulting in increased fracture incidence.					
Pain in extremity	Mild pain	Moderate pain; limiting instrumental ADL	Severe pain; limiting self care ADL	-	-
Definition: A disorder characterized by marked discomfort sensation in the upper or lower extremities.					

Musculoskeletal and connective tissue disorders					
	Grade				
Adverse Event	1	2	3	4	5
Pelvic soft tissue necrosis	-	Local wound care; medical intervention indicated (e.g., dressings or topical medications)	Operative debridement or other invasive intervention indicated (e.g., tissue reconstruction, flap or grafting)	Life-threatening consequences; urgent intervention indicated	Death
Definition: A disorder characterized by a necrotic process occurring in the soft tissues of the pelvis.					
Scoliosis	<20 degrees; clinically undetectable	>20 - 45 degrees; visible by forward flexion; limiting instrumental ADL	>45 degrees; scapular prominence in forward flexion; operative intervention indicated; limiting self care ADL; disabling	-	-
Definition: A disorder characterized by a malformed, lateral curvature of the spine.					
Soft tissue necrosis lower limb	-	Local wound care; medical intervention indicated (e.g., dressings or topical medications)	Operative debridement or other invasive intervention indicated (e.g., tissue reconstruction, flap or grafting)	Life-threatening consequences; urgent intervention indicated	Death
Definition: A disorder characterized by a necrotic process occurring in the soft tissues of the lower extremity.					
Soft tissue necrosis upper limb	-	Local wound care; medical intervention indicated (e.g., dressings or topical medications)	Operative debridement or other invasive intervention indicated (e.g., tissue reconstruction, flap or grafting)	Life-threatening consequences; urgent intervention indicated	Death

Musculoskeletal and connective tissue disorders

	Grade				
**Adverse Event**	**1**	**2**	**3**	**4**	**5**
Definition: A disorder characterized by a necrotic process occurring in the soft tissues of the upper extremity.					
Superficial soft tissue fibrosis	Mild induration, able to move skin parallel to plane (sliding) and perpendicular to skin (pinching up)	Moderate induration, able to slide skin, unable to pinch skin; limiting instrumental ADL	Severe induration; unable to slide or pinch skin; limiting joint or orifice movement (e.g., mouth, anus); limiting self care ADL	Generalized; associated with signs or symptoms of impaired breathing or feeding	Death
Definition: A disorder characterized by fibrotic degeneration of the superficial soft tissues.					
Trismus	Decreased ROM (range of motion) without impaired eating	Decreased ROM requiring small bites, soft foods or purees	Decreased ROM with inability to adequately aliment or hydrate orally	-	-
Definition: A disorder characterized by lack of ability to open the mouth fully due to a decrease in the range of motion of the muscles of mastication.					
Unequal limb length	Mild length discrepancy <2 cm	Moderate length discrepancy 2 - 5 cm; shoe lift indicated; limiting instrumental ADL	Severe length discrepancy >5 cm; limiting self care ADL; disabling; operative intervention indicated	-	-
Definition: A disorder characterized by of a discrepancy between the lengths of the lower or upper extremities.					
Musculoskeletal and connective tissue disorder - Other, specify	Asymptomatic or mild symptoms; clinical or diagnostic observations only; intervention not indicated	Moderate; minimal, local or noninvasive intervention indicated; limiting age-appropriate instrumental ADL	Severe or medically significant but not immediately life-threatening; hospitalization or prolongation of existing hospitalization indicated; disabling; limiting self care ADL	Life-threatening consequences; urgent intervention indicated	Death

Neoplasms benign, malignant and unspecified (incl cysts and polyps)

	Grade				
**Adverse Event**	**1**	**2**	**3**	**4**	**5**
Leukemia secondary to oncology chemotherapy	-	-	-	Present	Death
Definition: A disorder characterized by leukemia arising as a result of the mutagenic effect of chemotherapy agents.					
Myelodysplastic syndrome	-	-	-	Life-threatening consequences; urgent intervention indicated	Death
Definition: A disorder characterized by insufficiently healthy hematapoietic cell production by the bone marrow.					
Treatment related secondary malignancy	-	-	Non life-threatening secondary malignancy	Acute life-threatening secondary malignancy; blast crisis in leukemia	Death
Definition: A disorder characterized by development of a malignancy most probably as a result of treatment for a previously existing malignancy.					
Tumor pain	Mild pain	Moderate pain; limiting instrumental ADL	Severe pain; limiting self care ADL	-	-
Definition: A disorder characterized by marked discomfort from a neoplasm that may be pressing on a nerve, blocking blood vessels, inflamed or fractured from metastasis.					
Neoplasms benign, malignant and unspecified (incl cysts and polyps) - Other, specify	Asymptomatic or mild symptoms; clinical or diagnostic observations only; intervention not indicated	Moderate; minimal, local or noninvasive intervention indicated; limiting age-appropriate instrumental ADL	Severe or medically significant but not immediately life-threatening; hospitalization or prolongation of existing hospitalization indicated; disabling; limiting self care ADL	Life-threatening consequences; urgent intervention indicated	Death

Nervous system disorders					
	Grade				
**Adverse Event**	**1**	**2**	**3**	**4**	**5**
Abducens nerve disorder	Asymptomatic; clinical or diagnostic observations only; intervention not indicated	Moderate symptoms; limiting instrumental ADL	Severe symptoms; limiting self care ADL	-	-
Definition: A disorder characterized by involvement of the abducens nerve (sixth cranial nerve).					
Accessory nerve disorder	Asymptomatic; clinical or diagnostic observations only; intervention not indicated	Moderate symptoms; limiting instrumental ADL	Severe symptoms; limiting self care ADL	-	-
Definition: A disorder characterized by involvement of the accessory nerve (eleventh cranial nerve).					
Acoustic nerve disorder NOS	Asymptomatic; clinical or diagnostic observations only; intervention not indicated	Moderate symptoms; limiting instrumental ADL	Severe symptoms; limiting self care ADL	-	-
Definition: A disorder characterized by involvement of the acoustic nerve (eighth cranial nerve).					
Akathisia	Mild restlessness or increased motor activity	Moderate restlessness or increased motor activity; limiting instrumental ADL	Severe restlessness or increased motor activity; limiting self care ADL	-	-
Definition: A disorder characterized by an uncomfortable feeling of inner restlessness and inability to stay still; this is a side effect of some psychotropic drugs.					
Amnesia	Mild; transient memory loss	Moderate; short term memory loss; limiting instrumental ADL	Severe; long term memory loss; limiting self care ADL	-	-
Definition: A disorder characterized by systematic and extensive loss of memory.					
Aphonia	-	-	Voicelessness; unable to speak	-	-
Definition: A disorder characterized by the inability to speak. It may result from injuries to the vocal cords or may be functional (psychogenic).					

Nervous system disorders

	Grade				
**Adverse Event**	**1**	**2**	**3**	**4**	**5**
Arachnoiditis	Mild symptoms	Moderate symptoms; limiting instrumental ADL	Severe symptoms; limiting self care ADL	Life-threatening consequences; urgent intervention indicated	Death
Definition: A disorder characterized by inflammation of the arachnoid membrane and adjacent subarachnoid space.					
Ataxia	Asymptomatic; clinical or diagnostic observations only; intervention not indicated	Moderate symptoms; limiting instrumental ADL	Severe symptoms; limiting self care ADL; mechanical assistance indicated	-	-
Definition: A disorder characterized by lack of coordination of muscle movements resulting in the impairment or inability to perform voluntary activities.					
Brachial plexopathy	Asymptomatic; clinical or diagnostic observations only; intervention not indicated	Moderate symptoms; limiting instrumental ADL	Severe symptoms; limiting self care ADL	-	-
Definition: A disorder characterized by regional paresthesia of the brachial plexus, marked discomfort and muscle weakness, and limited movement in the arm or hand.					
Central nervous system necrosis	Asymptomatic; clinical or diagnostic observations only; intervention not indicated	Moderate symptoms; corticosteroids indicated	Severe symptoms; medical intervention indicated	Life-threatening consequences; urgent intervention indicated	Death
Definition: A disorder characterized by a necrotic process occurring in the brain and/or spinal cord.					
Cerebrospinal fluid leakage	Post-craniotomy: asymptomatic; Post-lumbar puncture: transient headache; postural care indicated	Post-craniotomy: moderate symptoms; medical intervention indicated; Post-lumbar puncture: persistent moderate symptoms; blood patch indicated	Severe symptoms; medical intervention indicated	Life-threatening consequences; urgent intervention indicated	Death
Definition: A disorder characterized by loss of cerebrospinal fluid into the surrounding tissues.					

Nervous system disorders

	Grade				
**Adverse Event**	**1**	**2**	**3**	**4**	**5**
Cognitive disturbance	Mild cognitive disability; not interfering with work/school/life performance; specialized educational services/devices not indicated	Moderate cognitive disability; interfering with work/school/life performance but capable of independent living; specialized resources on part time basis indicated	Severe cognitive disability; significant impairment of work/school/life performance	-	-
Definition: A disorder characterized by a conspicuous change in cognitive function.					
Concentration impairment	Mild inattention or decreased level of concentration	Moderate impairment in attention or decreased level of concentration; limiting instrumental ADL	Severe impairment in attention or decreased level of concentration; limiting self care ADL	-	-
Definition: A disorder characterized by a deterioration in the ability to concentrate.					
Depressed level of consciousness	Decreased level of alertness	Sedation; slow response to stimuli; limiting instrumental ADL	Difficult to arouse	Life-threatening consequences	Death
Definition: A disorder characterized by a decrease in ability to perceive and respond.					
Dizziness	Mild unsteadiness or sensation of movement	Moderate unsteadiness or sensation of movement; limiting instrumental ADL	Severe unsteadiness or sensation of movement; limiting self care ADL	-	-
Definition: A disorder characterized by a disturbing sensation of lightheadedness, unsteadiness, giddiness, spinning or rocking.					
Dysarthria	Mild slurred speech	Moderate impairment of articulation or slurred speech	Severe impairment of articulation or slurred speech	-	-
Definition: A disorder characterized by slow and slurred speech resulting from an inability to coordinate the muscles used in speech.					

Nervous system disorders

	Grade				
**Adverse Event**	**1**	**2**	**3**	**4**	**5**
Dysesthesia	Mild sensory alteration	Moderate sensory alteration; limiting instrumental ADL	Severe sensory alteration; limiting self care ADL	-	-
Definition: A disorder characterized by distortion of sensory perception, resulting in an abnormal and unpleasant sensation.					
Dysgeusia	Altered taste but no change in diet	Altered taste with change in diet (e.g., oral supplements); noxious or unpleasant taste; loss of taste	-	-	-
Definition: A disorder characterized by abnormal sensual experience with the taste of foodstuffs; it can be related to a decrease in the sense of smell.					
Dysphasia	Awareness of receptive or expressive characteristics; not impairing ability to communicate	Moderate receptive or expressive characteristics; impairing ability to communicate spontaneously	Severe receptive or expressive characteristics; impairing ability to read, write or communicate intelligibly	-	-
Definition: A disorder characterized by impairment of verbal communication skills, often resulting from brain damage.					
Edema cerebral	-	-	-	Life-threatening consequences; urgent intervention indicated	-
Definition: A disorder characterized by swelling due to an excessive accumulation of fluid in the brain.					
Encephalopathy	Mild symptoms	Moderate symptoms; limiting instrumental ADL	Severe symptoms; limiting self care ADL	Life-threatening consequences; urgent intervention indicated	Death
Definition: A disorder characterized by a pathologic process involving the brain.					

Nervous system disorders

	Grade				
**Adverse Event**	**1**	**2**	**3**	**4**	**5**
Extrapyramidal disorder	Mild involuntary movements	Moderate involuntary movements; limiting instrumental ADL	Severe involuntary movements or torticollis; limiting self care ADL	Life-threatening consequences; urgent intervention indicated	Death
Definition: A disorder characterized by abnormal, repetitive, involuntary muscle movements, frenzied speech and extreme restlessness.					
Facial muscle weakness	Asymptomatic; clinical or diagnostic observations only; intervention not indicated	Moderate symptoms; limiting instrumental ADL	Severe symptoms; limiting self care ADL	-	-
Definition: A disorder characterized by a reduction in the strength of the facial muscles.					
Facial nerve disorder	Asymptomatic; clinical or diagnostic observations only; intervention not indicated	Moderate symptoms; limiting instrumental ADL	Severe symptoms; limiting self care ADL	-	-
Definition: A disorder characterized by involvement of the facial nerve (seventh cranial nerve).					
Glossopharyngeal nerve disorder	Asymptomatic; clinical or diagnostic observations only; intervention not indicated	Moderate symptoms; limiting instrumental ADL	Severe symptoms; limiting self care ADL	Life-threatening consequences; urgent intervention indicated	Death
Definition: A disorder characterized by involvement of the glossopharyngeal nerve (ninth cranial nerve).					
Headache	Mild pain	Moderate pain; limiting instrumental ADL	Severe pain; limiting self care ADL	-	-
Definition: A disorder characterized by a sensation of marked discomfort in various parts of the head, not confined to the area of distribution of any nerve.					
Hydrocephalus	Asymptomatic; clinical or diagnostic observations only; intervention not indicated	Moderate symptoms; intervention not indicated	Severe symptoms or neurological deficit; intervention indicated	Life-threatening consequences; urgent intervention indicated	Death

Nervous system disorders

	Grade				
**Adverse Event**	**1**	**2**	**3**	**4**	**5**
Definition: A disorder characterized by an abnormal increase of cerebrospinal fluid in the ventricles of the brain.					
Hypersomnia	Mild increased need for sleep	Moderate increased need for sleep	Severe increased need for sleep	-	-
Definition: A disorder characterized by characterized by excessive sleepiness during the daytime.					
Hypoglossal nerve disorder	Asymptomatic; clinical or diagnostic observations only; intervention not indicated	Moderate symptoms; limiting instrumental ADL	Severe symptoms; limiting self care ADL	-	-
Definition: A disorder characterized by involvement of the hypoglossal nerve (twelfth cranial nerve).					
Intracranial hemorrhage	Asymptomatic; clinical or diagnostic observations only; intervention not indicated	Moderate symptoms; medical intervention indicated	Ventriculostomy, ICP monitoring, intraventricular thrombolysis, or operative intervention indicated	Life-threatening consequences; urgent intervention indicated	Death
Definition: A disorder characterized by bleeding from the cranium.					
Ischemia cerebrovascular	Asymptomatic; clinical or diagnostic observations only; intervention not indicated	Moderate symptoms	-	-	-
Definition: A disorder characterized by a decrease or absence of blood supply to the brain caused by obstruction (thrombosis or embolism) of an artery resulting in neurological damage.					
IVth nerve disorder	Asymptomatic; clinical or diagnostic observations only; intervention not indicated	Moderate symptoms; limiting instrumental ADL	Severe symptoms; limiting self care ADL	-	-
Definition: A disorder characterized by involvement of the trochlear nerve (fourth cranial nerve).					

Nervous system disorders

	Grade				
**Adverse Event**	**1**	**2**	**3**	**4**	**5**
Lethargy	Mild symptoms; reduced alertness and awareness	Moderate symptoms; limiting instrumental ADL	-	-	-
Definition: A disorder characterized by a decrease in consciousness characterized by mental and physical inertness.					
Leukoencephalopathy	Asymptomatic; small focal T2/FLAIR hyperintensities; involving periventricular white matter or <1/3 of susceptible areas of cerebrum +/- mild increase in subarachnoid space (SAS) and/or mild ventriculomegaly	Moderate symptoms; focal T2/FLAIR hyperintensities, involving periventricular white matter extending into centrum semiovale or involving 1/3 to 2/3 of susceptible areas of cerebrum +/- moderate increase in SAS and/or moderate ventriculomegaly	Severe symptoms; extensive T2/FLAIR hyperintensities, involving periventricular white matter involving 2/3 or more of susceptible areas of cerebrum +/- moderate to severe increase in SAS and/or moderate to severe ventriculomegaly	Life-threatening consequences; extensive T2/FLAIR hyperintensities, involving periventricular white matter involving most of susceptible areas of cerebrum +/- moderate to severe increase in SAS and/or moderate to severe ventriculomegaly	Death
Definition: A disorder characterized by diffuse reactive astrocytosis with multiple areas of necrotic foci without inflammation.					
Memory impairment	Mild memory impairment	Moderate memory impairment; limiting instrumental ADL	Severe memory impairment; limiting self care ADL	-	-
Definition: A disorder characterized by a deterioration in memory function.					
Meningismus	Mild symptoms	Moderate symptoms; limiting instrumental ADL	Severe symptoms; limiting self care ADL	Life-threatening consequences; urgent intervention indicated	Death
Definition: A disorder characterized by neck stiffness, headache, and photophobia resulting from irritation of the cerebral meninges.					

Nervous system disorders

	Grade				
**Adverse Event**	**1**	**2**	**3**	**4**	**5**
Movements involuntary	Mild symptoms	Moderate symptoms; limiting instrumental ADL	Severe symptoms; limiting self care ADL	-	-
Definition: A disorder characterized by uncontrolled and purposeless movements.					
Myelitis	Asymptomatic; mild signs (e.g., Babinski's reflex or Lhermitte's sign)	Moderate weakness or sensory loss; limiting instrumental ADL	Severe weakness or sensory loss; limiting self care ADL	Life-threatening consequences; urgent intervention indicated	Death
Definition: A disorder characterized by inflammation involving the spinal cord. Symptoms include weakness, paresthesia, sensory loss, marked discomfort and incontinence.					
Neuralgia	Mild pain	Moderate pain; limiting instrumental ADL	Severe pain; limiting self care ADL	-	-
Definition: A disorder characterized by intense painful sensation along a nerve or group of nerves.					
Nystagmus	-	Moderate symptoms; limiting instrumental ADL	Severe symptoms; limiting self care ADL	-	-
Definition: A disorder characterized by involuntary movements of the eyeballs.					
Oculomotor nerve disorder	Asymptomatic; clinical or diagnostic observations only; intervention not indicated	Moderate symptoms; limiting instrumental ADL	Severe symptoms; limiting self care ADL	-	-
Definition: A disorder characterized by involvement of the oculomotor nerve (third cranial nerve).					
Olfactory nerve disorder	-	Moderate symptoms; limiting instrumental ADL	Severe symptoms; limiting self care ADL	-	-
Definition: A disorder characterized by involvement of the olfactory nerve (first cranial nerve).					
Paresthesia	Mild symptoms	Moderate symptoms; limiting instrumental ADL	Severe symptoms; limiting self care ADL	-	-

Nervous system disorders

	Grade				
**Adverse Event**	**1**	**2**	**3**	**4**	**5**
Definition: A disorder characterized by functional disturbances of sensory neurons resulting in abnormal cutaneous sensations of tingling, numbness, pressure, cold, and warmth that are experienced in the absence of a stimulus.					
Peripheral motor neuropathy	Asymptomatic; clinical or diagnostic observations only; intervention not indicated	Moderate symptoms; limiting instrumental ADL	Severe symptoms; limiting self care ADL; assistive device indicated	Life-threatening consequences; urgent intervention indicated	Death
Definition: A disorder characterized by inflammation or degeneration of the peripheral motor nerves.					
Peripheral sensory neuropathy	Asymptomatic; loss of deep tendon reflexes or paresthesia	Moderate symptoms; limiting instrumental ADL	Severe symptoms; limiting self care ADL	Life-threatening consequences; urgent intervention indicated	Death
Definition: A disorder characterized by inflammation or degeneration of the peripheral sensory nerves.					
Phantom pain	Mild pain	Moderate pain; limiting instrumental ADL	Severe pain; limiting self care ADL	-	-
Definition: A disorder characterized by marked discomfort related to a limb or an organ that is removed from or is not physically part of the body.					
Presyncope	-	Present (e.g., near fainting)	-	-	-
Definition: A disorder characterized by an episode of lightheadedness and dizziness which may precede an episode of syncope.					
Pyramidal tract syndrome	Asymptomatic; clinical or diagnostic observations only; intervention not indicated	Moderate symptoms; limiting instrumental ADL	Severe symptoms; limiting self care ADL	Life-threatening consequences; urgent intervention indicated	Death
Definition: A disorder characterized by dysfunction of the corticospinal (pyramidal) tracts of the spinal cord. Symptoms include an increase in the muscle tone in the lower extremities, hyperreflexia, positive Babinski and a decrease in fine motor coordination.					

Nervous system disorders

	Grade				
**Adverse Event**	**1**	**2**	**3**	**4**	**5**
Radiculitis	Mild symptoms	Moderate symptoms; limiting instrumental ADL; medical intervention indicated	Severe symptoms; limiting self care ADL	Life-threatening consequences; urgent intervention indicated	Death
Definition: A disorder characterized by inflammation involving a nerve root. Patients experience marked discomfort radiating along a nerve path because of spinal pressure on the connecting nerve root.					
Recurrent laryngeal nerve palsy	Asymptomatic; clinical or diagnostic observations only; intervention not indicated	Moderate symptoms	Severe symptoms; medical intervention indicated (e.g., thyroplasty, vocal cord injection)	Life-threatening consequences; urgent intervention indicated	Death
Definition: A disorder characterized by paralysis of the recurrent laryngeal nerve.					
Reversible posterior leukoencephalopathy syndrome	Asymptomatic; clinical or diagnostic observations only; intervention not indicated	Moderate symptoms; abnormal imaging studies; limiting instrumental ADL	Severe symptoms; very abnormal imaging studies; limiting self care ADL	Life-threatening consequences; urgent intervention indicated	Death
Definition: A disorder characterized by headaches, mental status changes, visual disturbances, and seizures associated with imaging findings of posterior leukoencephalopathy. It has been observed in association with hypertensive encephalopathy, eclampsia, and immunosuppressive and cytotoxic drug treatment. It is an acute or subacute reversible condition.					
Seizure	Brief partial seizure; no loss of consciousness	Brief generalized seizure	Multiple seizures despite medical intervention	Life-threatening; prolonged repetitive seizures	Death
Definition: A disorder characterized by a sudden, involuntary skeletal muscular contractions of cerebral or brain stem origin.					
Sinus pain	Mild pain	Moderate pain; limiting instrumental ADL	Severe pain; limiting self care ADL	-	-
Definition: A disorder characterized by marked discomfort in the face, between the eyes, or upper teeth originating from the sinuses.					

Nervous system disorders

	Grade				
**Adverse Event**	**1**	**2**	**3**	**4**	**5**
Somnolence	Mild but more than usual drowsiness or sleepiness	Moderate sedation; limiting instrumental ADL	Obtundation or stupor	Life-threatening consequences; urgent intervention indicated	Death
Definition: A disorder characterized by characterized by excessive sleepiness and drowsiness.					
Spasticity	Mild or slight increase in muscle tone	Moderate increase in muscle tone and increase in resistance through range of motion	Severe increase in muscle tone and increase in resistance through range of motion	Life-threatening; unable to move active or passive range of motion	Death
Definition: A disorder characterized by increased involuntary muscle tone that affects the regions interfering with voluntary movement. It results in gait, movement, and speech disturbances.					
Stroke	Asymptomatic or mild neurologic deficit; radiographic findings only	Moderate neurologic deficit	Severe neurologic deficit	Life-threatening consequences; urgent intervention indicated	Death
Definition: A disorder characterized by a sudden loss of sensory function due to an intracranial vascular event.					
Syncope	-	-	Fainting; orthostatic collapse	-	-
Definition: A disorder characterized by spontaneous loss of consciousness caused by insufficient blood supply to the brain.					
Transient ischemic attacks	Mild neurologic deficit with or without imaging confirmation	Moderate neurologic deficit with or without imaging confirmation	-	-	-
Definition: A disorder characterized by a brief attack (less than 24 hours) of cerebral dysfunction of vascular origin, with no persistent neurological deficit.					
Tremor	Mild symptoms	Moderate symptoms; limiting instrumental ADL	Severe symptoms; limiting self care ADL	-	-

Nervous system disorders

	Grade				
**Adverse Event**	**1**	**2**	**3**	**4**	**5**
Definition: A disorder characterized by the uncontrolled shaking movement of the whole body or individual parts.					
Trigeminal nerve disorder	Asymptomatic; clinical or diagnostic observations only; intervention not indicated	Moderate symptoms; limiting instrumental ADL	Severe symptoms; limiting self care ADL	-	-
Definition: A disorder characterized by involvement of the trigeminal nerve (fifth cranial nerve).					
Vagus nerve disorder	Asymptomatic; clinical or diagnostic observations only; intervention not indicated	Moderate symptoms; limiting instrumental ADL	Severe symptoms; limiting self care ADL	Life-threatening consequences; urgent intervention indicated	Death
Definition: A disorder characterized by involvement of the vagus nerve (tenth cranial nerve).					
Vasovagal reaction	-	-	Present	Life-threatening consequences; urgent intervention indicated	Death
Definition: A disorder characterized by a sudden drop of the blood pressure, bradycardia, and peripheral vasodilation that may lead to loss of consciousness. It results from an increase in the stimulation of the vagus nerve.					
Nervous system disorders - Other, specify	Asymptomatic or mild symptoms; clinical or diagnostic observations only; intervention not indicated	Moderate; minimal, local or noninvasive intervention indicated; limiting age-appropriate instrumental ADL	Severe or medically significant but not immediately life-threatening; hospitalization or prolongation of existing hospitalization indicated; disabling; limiting self care ADL	Life-threatening consequences; urgent intervention indicated	Death

Pregnancy, puerperium and perinatal conditions

	Grade				
**Adverse Event**	**1**	**2**	**3**	**4**	**5**
Fetal death	-	-	-	-	Fetal loss at any gestational age
Definition: A disorder characterized by death in utero; failure of the product of conception to show evidence of respiration, heartbeat, or definite movement of a voluntary muscle after expulsion from the uterus, without possibility of resuscitation.					
Fetal growth retardation	-	<10% percentile of weight for gestational age	<5% percentile of weight for gestational age	<1% percentile of weight for gestational age	-
Definition: A disorder characterized by inhibition of fetal growth resulting in the inability of the fetus to achieve its potential weight.					
Premature delivery	Delivery of a liveborn infant at >34 to 37 weeks gestation	Delivery of a liveborn infant at >28 to 34 weeks gestation	Delivery of a liveborn infant at 24 to 28 weeks gestation	Delivery of a liveborn infant at 24 weeks of gestation or less	-
Definition: A disorder characterized by delivery of a viable infant before the normal end of gestation. Typically, viability is achievable between the twentieth and thirty-seventh week of gestation.					
Unintended pregnancy	-	-	Unintended pregnancy	-	-
Definition: A disorder characterized by an unexpected pregnancy at the time of conception.					
Pregnancy, puerperium and perinatal conditions - Other, specify	Asymptomatic or mild symptoms; clinical or diagnostic observations only; intervention not indicated	Moderate, local or noninvasive intervention indicated; limiting instrumental ADL	Severe or medically significant but not immediately life-threatening; hospitalization or prolongation of existing hospitalization indicated; disabling; limiting self care ADL	Life-threatening consequences; urgent intervention indicated	Death

Psychiatric disorders

	Grade				
**Adverse Event**	**1**	**2**	**3**	**4**	**5**
Agitation	Mild mood alteration	Moderate mood alteration	Severe agitation; hospitalization not indicated	Life-threatening consequences; urgent intervention indicated	Death
Definition: A disorder characterized by a state of restlessness associated with unpleasant feelings of irritability and tension.					
Anorgasmia	Inability to achieve orgasm not adversely affecting relationship	Inability to achieve orgasm adversely affecting relationship	-	-	-
Definition: A disorder characterized by an inability to achieve orgasm.					
Anxiety	Mild symptoms; intervention not indicated	Moderate symptoms; limiting instrumental ADL	Severe symptoms; limiting self care ADL; hospitalization not indicated	Life-threatening; hospitalization indicated	Death
Definition: A disorder characterized by apprehension of danger and dread accompanied by restlessness, tension, tachycardia, and dyspnea unattached to a clearly identifiable stimulus.					
Confusion	Mild disorientation	Moderate disorientation; limiting instrumental ADL	Severe disorientation; limiting self care ADL	Life-threatening consequences; urgent intervention indicated	Death
Definition: A disorder characterized by a lack of clear and orderly thought and behavior.					
Delayed orgasm	Delay in achieving orgasm not adversely affecting relationship	Delay in achieving orgasm adversely affecting relationship	-	-	-
Definition: A disorder characterized by sexual dysfunction characterized by a delay in climax.					

Psychiatric disorders

	Grade				
**Adverse Event**	**1**	**2**	**3**	**4**	**5**
Delirium	Mild acute confusional state	Moderate and acute confusional state; limiting instrumental ADL	Severe and acute confusional state; limiting self care ADL; hospitalization indicated	Life-threatening consequences, threats of harm to self or others; hospitalization indicated	Death
Definition: A disorder characterized by the acute and sudden development of confusion, illusions, movement changes, inattentiveness, agitation, and hallucinations. Usually, it is a reversible condition.					
Delusions	-	Moderate delusional symptoms	Severe delusional symptoms; hospitalization not indicated	Life-threatening consequences, threats of harm to self or others; hospitalization indicated	Death
Definition: A disorder characterized by false personal beliefs held contrary to reality, despite contradictory evidence and common sense.					
Depression	Mild depressive symptoms	Moderate depressive symptoms; limiting instrumental ADL	Severe depressive symptoms; limiting self care ADL; hospitalization not indicated	Life-threatening consequences, threats of harm to self or others; hospitalization indicated	Death
Definition: A disorder characterized by melancholic feelings of grief or unhappiness.					
Euphoria	Mild mood elevation	Moderate mood elevation	Severe mood elevation (e.g., hypomania)	-	-
Definition: A disorder characterized by an exaggerated feeling of well-being which is disproportionate to events and stimuli.					
Hallucinations	Mild hallucinations (e.g., perceptual distortions)	Moderate hallucinations	Severe hallucinations; hospitalization not indicated	Life-threatening consequences, threats of harm to self or others; hospitalization indicated	Death

Psychiatric disorders

	Grade				
**Adverse Event**	**1**	**2**	**3**	**4**	**5**
Definition: A disorder characterized by a false sensory perception in the absence of an external stimulus.					
Insomnia	Mild difficulty falling asleep, staying asleep or waking up early	Moderate difficulty falling asleep, staying asleep or waking up early	Severe difficulty in falling asleep, staying asleep or waking up early	-	-
Definition: A disorder characterized by difficulty in falling asleep and/or remaining asleep.					
Libido decreased	Decrease in sexual interest not adversely affecting relationship	Decrease in sexual interest adversely affecting relationship	-	-	-
Definition: A disorder characterized by a decrease in sexual desire.					
Libido increased	Mild increase in sexual interest not adversely affecting relationship	Moderate increase in sexual interest adversely affecting relationship	Severe increase in sexual interest leading to dangerous behavior	-	-
Definition: A disorder characterized by an increase in sexual desire.					
Mania	Mild manic symptoms (e.g., elevated mood, rapid thoughts, rapid speech, decreased need for sleep)	Moderate manic symptoms (e.g., relationship and work difficulties; poor hygiene)	Severe manic symptoms (e.g., hypomania; major sexual or financial indiscretions); hospitalization not indicated	Life-threatening consequences, threats of harm to self or others; hospitalization indicated	Death
Definition: A disorder characterized by excitement of psychotic proportions manifested by mental and physical hyperactivity, disorganization of behavior and elevation of mood.					
Personality change	Mild personality change	Moderate personality change	Severe personality change; hospitalization not indicated	Life-threatening consequences, threats of harm to self or others; hospitalization indicated	Death

Psychiatric disorders

	Grade				
**Adverse Event**	**1**	**2**	**3**	**4**	**5**
Definition: A disorder characterized by a conspicuous change in a person's behavior and thinking.					
Psychosis	Mild psychotic symptoms	Moderate psychotic symptoms (e.g., disorganized speech; impaired reality testing)	Severe psychotic symptoms (e.g., paranoid; extreme disorganization); hospitalization not indicated	Life-threatening consequences, threats of harm to self or others; hospitalization indicated	Death
Definition: A disorder characterized by personality change, impaired functioning, and loss of touch with reality. It may be a manifestation of schizophrenia, bipolar disorder or brain tumor.					
Restlessness	Mild symptoms; intervention not indicated	Moderate symptoms; limiting instrumental ADL	Severe symptoms; limiting self care ADL	-	-
Definition: A disorder characterized by an inability to rest, relax or be still.					
Suicidal ideation	Increased thoughts of death but no wish to kill oneself	Suicidal ideation with no specific plan or intent	Specific plan to commit suicide without serious intent to die which may not require hospitalization	Specific plan to commit suicide with serious intent to die which requires hospitalization	-
Definition: A disorder characterized by thoughts of taking one's own life.					
Suicide attempt	-	-	Suicide attempt or gesture without intent to die which may not require hospitalization	Suicide attempt with intent to die which requires hospitalization	Death
Definition: A disorder characterized by self-inflicted harm in an attempt to end one's own life.					

Psychiatric disorders

	Grade				
**Adverse Event**	**1**	**2**	**3**	**4**	**5**
Psychiatric disorders - Other, specify	Asymptomatic or mild symptoms; clinical or diagnostic observations only; intervention not indicated	Moderate; minimal, local or noninvasive intervention indicated; limiting age-appropriate instrumental ADL	Severe or medically significant but not immediately life-threatening; disabling; limiting self care ADL	Life-threatening consequences; hospitalization or urgent intervention indicated	Death

Renal and urinary disorders					
	Grade				
**Adverse Event**	**1**	**2**	**3**	**4**	**5**
Acute kidney injury	Creatinine level increase of >0.3 mg/dL; creatinine 1.5 - 2.0 x above baseline	Creatinine 2 - 3 x above baseline	Creatinine >3 x baseline or >4.0 mg/dL; hospitalization indicated	Life-threatening consequences; dialysis indicated	Death
Definition: A disorder characterized by the acute loss of renal function and is traditionally classified as pre-renal (low blood flow into kidney), renal (kidney damage) and post-renal causes (ureteral or bladder outflow obstruction).					
Bladder perforation	-	Extraperitoneal perforation, indwelling catheter indicated	Intraperitoneal perforation; elective radiologic, endoscopic or operative intervention indicated	Life-threatening consequences; organ failure; urgent operative intervention indicated	Death
Definition: A disorder characterized by a rupture in the bladder wall.					
Bladder spasm	Intervention not indicated	Antispasmodics indicated	Hospitalization indicated	-	-
Definition: A disorder characterized by a sudden and involuntary contraction of the bladder wall.					
Chronic kidney disease	eGFR (estimated Glomerular Filtration Rate) or CrCl (creatinine clearance) <LLN - 60 ml/min/1.73 m2 or proteinuria 2+ present; urine protein/creatinine >0.5	eGFR or CrCl 59 - 30 ml/min/1.73 m2	eGFR or CrCl 29 - 15 ml/min/1.73 m2	eGFR or CrCl <15 ml/min/1.73 m2; dialysis or renal transplant indicated	Death
Definition: A disorder characterized by gradual and usually permanent loss of kidney function resulting in renal failure.					

Renal and urinary disorders

	Grade				
**Adverse Event**	**1**	**2**	**3**	**4**	**5**
Cystitis noninfective	Microscopic hematuria; minimal increase in frequency, urgency, dysuria, or nocturia; new onset of incontinence	Moderate hematuria; moderate increase in frequency, urgency, dysuria, nocturia or incontinence; urinary catheter placement or bladder irrigation indicated; limiting instrumental ADL	Gross hematuria; transfusion, IV medications or hospitalization indicated; elective endoscopic, radiologic or operative intervention indicated	Life-threatening consequences; urgent radiologic or operative intervention indicated	Death
Definition: A disorder characterized by inflammation of the bladder which is not caused by an infection of the urinary tract.					
Hematuria	Asymptomatic; clinical or diagnostic observations only; intervention not indicated	Symptomatic; urinary catheter or bladder irrigation indicated; limiting instrumental ADL	Gross hematuria; transfusion, IV medications or hospitalization indicated; elective endoscopic, radiologic or operative intervention indicated; limiting self care ADL	Life-threatening consequences; urgent radiologic or operative intervention indicated	Death
Definition: A disorder characterized by laboratory test results that indicate blood in the urine.					
Hemoglobinuria	Asymptomatic; clinical or diagnostic observations only; intervention not indicated	-	-	-	-
Definition: A disorder characterized by laboratory test results that indicate the presence of free hemoglobin in the urine.					

Renal and urinary disorders

	Grade				
**Adverse Event**	**1**	**2**	**3**	**4**	**5**
Proteinuria	1+ proteinuria; urinary protein <1.0 g/24 hrs	Adults: 2+ proteinuria; urinary protein 1.0 - 3.4 g/24 hrs;<br>Pediatric: urine P/C (Protein/Creatinine) ratio 0.5 - 1.9	Adults: urinary protein >=3.5 g/24 hrs;<br>Pediatric: urine P/C >1.9	-	-
Definition: A disorder characterized by laboratory test results that indicate the presence of excessive protein in the urine. It is predominantly albumin, but also globulin.					
Renal calculi	Asymptomatic or mild symptoms; occasional use of nonprescription analgesics indicated	Symptomatic; oral antiemetics indicated; around the clock nonprescription analgesics or any oral narcotic analgesics indicated	Hospitalization indicated; IV intervention (e.g., analgesics, antiemetics); elective endoscopic or radiologic intervention indicated	Life-threatening consequences; urgent radiologic, endoscopic or operative intervention indicated	Death
Definition: A disorder characterized by the formation of crystals in the pelvis of the kidney.					
Renal colic	Mild pain not interfering with activity; nonprescription medication indicated	Moderate pain; limiting instrumental ADL; prescription medication indicated	Hospitalization indicated; limiting self care ADL	-	-
Definition: A disorder characterized by paroxysmal and severe flank marked discomfort radiating to the inguinal area. Often, the cause is the passage of kidney stones.					
Renal hemorrhage	Mild symptoms; intervention not indicated	Analgesics and hematocrit monitoring indicated	Transfusion, radiation, or hospitalization indicated; elective radiologic, endoscopic or operative intervention indicated	Life-threatening consequences; urgent radiologic or operative intervention indicated	Death
Definition: A disorder characterized by bleeding from the kidney.					

Renal and urinary disorders

	Grade				
**Adverse Event**	**1**	**2**	**3**	**4**	**5**
Urinary fistula	-	Noninvasive intervention indicated; urinary or suprapubic catheter placement indicated	Limiting self care ADL; elective radiologic, endoscopic or operative intervention indicated; permanent urinary diversion indicated	Life-threatening consequences; urgent radiologic or operative intervention indicated	Death
Definition: A disorder characterized by an abnormal communication between any part of the urinary system and another organ or anatomic site.					
Urinary frequency	Present	Limiting instrumental ADL; medical management indicated	-	-	-
Definition: A disorder characterized by urination at short intervals.					
Urinary incontinence	Occasional (e.g., with coughing, sneezing, etc.), pads not indicated	Spontaneous; pads indicated; limiting instrumental ADL	Intervention indicated (e.g., clamp, collagen injections); operative intervention indicated; limiting self care ADL	-	-
Definition: A disorder characterized by inability to control the flow of urine from the bladder.					
Urinary retention	Urinary, suprapubic or intermittent catheter placement not indicated; able to void with some residual	Placement of urinary, suprapubic or intermittent catheter placement indicated; medication indicated	Elective operative or radiologic intervention indicated; substantial loss of affected kidney function or mass	Life-threatening consequences; organ failure; urgent operative intervention indicated	Death
Definition: A disorder characterized by accumulation of urine within the bladder because of the inability to urinate.					

Renal and urinary disorders

	Grade				
**Adverse Event**	**1**	**2**	**3**	**4**	**5**
Urinary tract obstruction	Asymptomatic; clinical or diagnostic observations only	Symptomatic but no hydronephrosis, sepsis or renal dysfunction; urethral dilation, urinary or suprapubic catheter indicated	Symptomatic and altered organ function (e.g., hydronephrosis, or renal dysfunction); elective radiologic, endoscopic or operative intervention indicated	Life-threatening consequences; urgent intervention indicated	Death
Definition: A disorder characterized by blockage of the normal flow of contents of the urinary tract.					
Urinary tract pain	Mild pain	Moderate pain; limiting instrumental ADL	Severe pain; limiting self care ADL	-	-
Definition: A disorder characterized by a sensation of marked discomfort in the urinary tract.					
Urinary urgency	Present	Limiting instrumental ADL; medical management indicated	-	-	-
Definition: A disorder characterized by a sudden compelling urge to urinate.					
Urine discoloration	Present	-	-	-	-
Definition: A disorder characterized by a change in the color of the urine.					

Renal and urinary disorders

	Grade				
**Adverse Event**	**1**	**2**	**3**	**4**	**5**
Renal and urinary disorders - Other, specify	Asymptomatic or mild symptoms; clinical or diagnostic observations only; intervention not indicated	Moderate, local or noninvasive intervention indicated; limiting instrumental ADL	Severe or medically significant but not immediately life-threatening; hospitalization or prolongation of existing hospitalization indicated; disabling; limiting self care ADL	Life-threatening consequences; urgent intervention indicated	Death

Reproductive system and breast disorders

	Grade				
**Adverse Event**	**1**	**2**	**3**	**4**	**5**
Azoospermia	-	-	Absence of sperm in ejaculate	-	-
Definition: A disorder characterized by laboratory test results that indicate complete absence of spermatozoa in the semen.					
Breast atrophy	Minimal asymmetry; minimal atrophy	Moderate asymmetry; moderate atrophy	Asymmetry >1/3 of breast volume; severe atrophy	-	-
Definition: A disorder characterized by underdevelopment of the breast.					
Breast pain	Mild pain	Moderate pain; limiting instrumental ADL	Severe pain; limiting self care ADL	-	-
Definition: A disorder characterized by marked discomfort sensation in the breast region.					
Dysmenorrhea	Mild symptoms; intervention not indicated	Moderate symptoms; limiting instrumental ADL	Severe symptoms; limiting self care ADL	-	-
Definition: A disorder characterized by abnormally painful abdominal cramps during menses.					
Dyspareunia	Mild discomfort or pain associated with vaginal penetration; discomfort relieved with use of vaginal lubricants or estrogen	Moderate discomfort or pain associated with vaginal penetration; discomfort or pain partially relieved with use of vaginal lubricants or estrogen	Severe discomfort or pain associated with vaginal penetration; discomfort or pain unrelieved by vaginal lubricants or estrogen	-	-
Definition: A disorder characterized by painful or difficult coitus.					
Ejaculation disorder	Diminished ejaculation	Anejaculation or retrograde ejaculation	-	-	-
Definition: A disorder characterized by problems related to ejaculation. This category includes premature, delayed, retrograde and painful ejaculation.					

Reproductive system and breast disorders

	Grade				
**Adverse Event**	**1**	**2**	**3**	**4**	**5**
Erectile dysfunction	Decrease in erectile function (frequency or rigidity of erections) but intervention not indicated (e.g., medication or use of mechanical device, penile pump)	Decrease in erectile function (frequency/rigidity of erections), erectile intervention indicated, (e.g., medication or mechanical devices such as penile pump)	Decrease in erectile function (frequency/rigidity of erections) but erectile intervention not helpful (e.g., medication or mechanical devices such as penile pump); placement of a permanent penile prosthesis indicated (not previously present)	-	-
Definition: A disorder characterized by the persistent or recurrent inability to achieve or to maintain an erection during sexual activity.					
Fallopian tube obstruction	Diagnostic observations only; intervention not indicated	Mild symptoms; elective intervention indicated	Severe symptoms; elective operative intervention indicated	-	-
Definition: A disorder characterized by blockage of the normal flow of the contents in the fallopian tube.					
Fallopian tube stenosis	Asymptomatic clinical or diagnostic observations only; intervention not indicated	Symptomatic and intervention not indicated	Severe symptoms; elective operative intervention indicated	Life-threatening consequences; urgent operative intervention indicated (e.g., organ resection)	Death
Definition: A disorder characterized by a narrowing of the fallopian tube lumen.					
Female genital tract fistula	Asymptomatic clinical or diagnostic observations only; intervention not indicated	Symptomatic and intervention not indicated	Severe symptoms; elective operative intervention indicated	Life-threatening consequences; urgent intervention indicated	Death

Reproductive system and breast disorders					
	Grade				
**Adverse Event**	**1**	**2**	**3**	**4**	**5**
Definition: A disorder characterized by an abnormal communication between a female reproductive system organ and another organ or anatomic site.					
Feminization acquired	Mild symptoms; intervention not indicated	Moderate symptoms; medical intervention indicated	-	-	-
Definition: A disorder characterized by the development of secondary female sex characteristics in males due to extrinsic factors.					
Genital edema	Mild swelling or obscuration of anatomic architecture on close inspection	Readily apparent obscuration of anatomic architecture; obliteration of skin folds; readily apparent deviation from normal anatomic contour	Lymphorrhea; gross deviation from normal anatomic contour; limiting self care ADL	-	-
Definition: A disorder characterized by swelling due to an excessive accumulation of fluid in the genitals.					
Gynecomastia	Asymptomatic breast enlargement	Symptomatic (e.g., pain or psychosocial impact)	Severe symptoms; elective operative intervention indicated	-	-
Definition: A disorder characterized by excessive development of the breasts in males.					
Hematosalpinx	Minimal bleeding identified on imaging study or laparoscopy; intervention not indicated	Moderate bleeding; medical intervention indicated	Severe bleeding; transfusion indicated; radiologic or endoscopic intervention indicated	Life-threatening consequences; urgent operative intervention indicated	Death
Definition: A disorder characterized by the presence of blood in a fallopian tube.					
Irregular menstruation	Intermittent menses with skipped menses for no more than 1 to 3 months	Intermittent menses with skipped menses for more than 4 to 6 months	Persistent amenorrhea for more than 6 months	-	-

Reproductive system and breast disorders

	Grade				
**Adverse Event**	**1**	**2**	**3**	**4**	**5**
Definition: A disorder characterized by irregular cycle or duration of menses.					
Lactation disorder	Mild changes in lactation, not significantly affecting production or expression of breast milk	Changes in lactation, significantly affecting breast production or expression of breast milk	-	-	-
Definition: A disorder characterized by disturbances of milk secretion. It is not necessarily related to pregnancy that is observed in females and can be observed in males.					
Menorrhagia	Mild; iron supplements indicated	Moderate symptoms; medical intervention indicated (e.g., hormones)	Severe; transfusion indicated; surgical intervention indicated (e.g., hysterectomy)	Life-threatening consequences; urgent intervention indicated	Death
Definition: A disorder characterized by abnormally heavy vaginal bleeding during menses.					
Nipple deformity	Asymptomatic; asymmetry with slight retraction and/or thickening of the nipple areolar complex	Symptomatic; asymmetry of nipple areolar complex with moderate retraction and/or thickening of the nipple areolar complex	-	-	-
Definition: A disorder characterized by a malformation of the nipple.					
Oligospermia	Sperm concentration >48 million/mL or motility >68%	Sperm concentration 13 - 48 million/mL or motility 32 - 68%	Sperm concentration <13 million/mL or motility <32%	-	-
Definition: A disorder characterized by a decrease in the number of spermatozoa in the semen.					
Ovarian hemorrhage	Minimal bleeding identified on imaging study or laproscopy; intervention not indicated	Moderate bleeding; medical intervention indicated	Severe bleeding; transfusion indicated; radiologic or endoscopic intervention indicated	Life-threatening consequences; urgent operative intervention indicated	Death

Reproductive system and breast disorders					
	Grade				
**Adverse Event**	**1**	**2**	**3**	**4**	**5**
Definition: A disorder characterized by bleeding from the ovary.					
Ovarian rupture	Asymptomatic clinical or diagnostic observations only; intervention not indicated	Symptomatic and intervention not indicated	Transfusion, radiologic, endoscopic, or elective operative intervention indicated	Life-threatening consequences; urgent intervention indicated	Death
Definition: A disorder characterized by tearing or disruption of the ovarian tissue.					
Ovulation pain	Mild pain	Moderate pain; limiting instrumental ADL	Severe pain; limiting self care ADL	-	-
Definition: A disorder characterized by marked discomfort sensation in one side of the abdomen between menstrual cycles, around the time of the discharge of the ovum from the ovarian follicle.					
Pelvic floor muscle weakness	Asymptomatic; clinical or diagnostic observations only; intervention not indicated	Symptomatic, not interfering with bladder, bowel, or vaginal function; limiting instrumental ADL	Severe symptoms; limiting self care ADL	Life-threatening consequences; urgent intervention indicated	Death
Definition: A disorder characterized by a reduction in the strength of the muscles of the pelvic floor.					
Pelvic pain	Mild pain	Moderate pain; limiting instrumental ADL	Severe pain; limiting self care ADL	-	-
Definition: A disorder characterized by marked discomfort sensation in the pelvis.					
Penile pain	Mild pain	Moderate pain; limiting instrumental ADL	Severe pain; limiting self care ADL	-	-
Definition: A disorder characterized by marked discomfort sensation in the penis.					

Reproductive system and breast disorders

	Grade				
**Adverse Event**	**1**	**2**	**3**	**4**	**5**
Perineal pain	Mild pain	Moderate pain; limiting instrumental ADL	Severe pain; limiting self care ADL	-	-
Definition: A disorder characterized by a sensation of marked discomfort in the area between the genital organs and the anus.					
Premature menopause	-	-	Present	-	-
Definition: A disorder characterized by ovarian failure before the age of 40. Symptoms include hot flashes, night sweats, mood swings and a decrease in sex drive.					
Prostatic hemorrhage	Minimal bleeding identified on imaging study; intervention not indicated	Moderate bleeding; medical intervention indicated	Severe bleeding; transfusion indicated; radiologic or endoscopic intervention indicated	Life-threatening consequences; urgent operative intervention indicated	Death
Definition: A disorder characterized by bleeding from the prostate gland.					
Prostatic obstruction	Diagnostic observations only; intervention not indicated	Mild symptoms; elective intervention indicated	Severe symptoms; elective operative intervention indicated	-	-
Definition: A disorder characterized by compression of the urethra secondary to enlargement of the prostate gland. This results in voiding difficulties (straining to void, slow urine stream, and incomplete emptying of the bladder).					
Prostatic pain	Mild pain	Moderate pain; limiting instrumental ADL	Severe pain; limiting self care ADL	-	-
Definition: A disorder characterized by a sensation of marked discomfort in the prostate gland.					
Scrotal pain	Mild pain	Moderate pain; limiting instrumental ADL	Severe pain; limiting self care ADL	-	-
Definition: A disorder characterized by marked discomfort sensation in the scrotal area.					

Reproductive system and breast disorders

	Grade				
**Adverse Event**	**1**	**2**	**3**	**4**	**5**
Spermatic cord hemorrhage	Minimal bleeding identified on imaging study; intervention not indicated	Moderate bleeding; medical intervention indicated	Severe bleeding; transfusion indicated; radiologic or endoscopic intervention indicated	Life-threatening consequences; urgent operative intervention indicated	Death
Definition: A disorder characterized by bleeding from the spermatic cord.					
Spermatic cord obstruction	Diagnostic observations only; intervention not indicated	Mild symptoms; elective intervention indicated	Severe symptoms; elective operative intervention indicated	-	-
Definition: A disorder characterized by blockage of the normal flow of the contents of the spermatic cord.					
Testicular disorder	Asymptomatic; clinical or diagnostic observations only; intervention not indicated	Symptomatic but not interfering with urination or sexual activities; intervention not indicated; limiting instrumental ADL	Severe symptoms; interfering with urination or sexual function; limiting self care ADL; intervention indicated	Life-threatening consequences; urgent intervention indicated	Death
Definition: A disorder characterized by involvement of the testis.					
Testicular hemorrhage	Minimal bleeding identified on imaging study; intervention not indicated	Moderate bleeding; medical intervention indicated	Severe bleeding; transfusion indicated; radiologic or endoscopic intervention indicated	Life-threatening consequences; urgent operative intervention indicated	Death
Definition: A disorder characterized by bleeding from the testis.					
Testicular pain	Mild pain	Moderate pain; limiting instrumental ADL	Severe pain; limiting self care ADL	-	-
Definition: A disorder characterized by a sensation of marked discomfort in the testis.					

Reproductive system and breast disorders

	Grade				
**Adverse Event**	**1**	**2**	**3**	**4**	**5**
Uterine fistula	Asymptomatic clinical or diagnostic observations only; intervention not indicated	Symptomatic and intervention not indicated	Severe symptoms; elective operative intervention indicated	Life-threatening consequences; urgent intervention indicated	Death
Definition: A disorder characterized by an abnormal communication between the uterus and another organ or anatomic site.					
Uterine hemorrhage	Minimal bleeding identified on imaging study; intervention not indicated	Moderate bleeding; medical intervention indicated	Severe bleeding; transfusion indicated; radiologic or endoscopic intervention indicated	Life-threatening consequences; urgent operative intervention indicated	Death
Definition: A disorder characterized by bleeding from the uterus.					
Uterine obstruction	Diagnostic observations only; intervention not indicated	Mild symptoms; elective intervention indicated	Severe symptoms; elective operative intervention indicated	-	-
Definition: A disorder characterized by blockage of the uterine outlet.					
Uterine pain	Mild pain	Moderate pain; limiting instrumental ADL	Severe pain; limiting self care ADL	-	-
Definition: A disorder characterized by a sensation of marked discomfort in the uterus.					
Vaginal discharge	Mild vaginal discharge (greater than baseline for patient)	Moderate to heavy vaginal discharge; use of perineal pad or tampon indicated	-	-	-
Definition: A disorder characterized by vaginal secretions. Mucus produced by the cervical glands is discharged from the vagina naturally, especially during the childbearing years.					

Reproductive system and breast disorders

	Grade				
**Adverse Event**	**1**	**2**	**3**	**4**	**5**
Vaginal dryness	Mild vaginal dryness not interfering with sexual function	Moderate vaginal dryness interfering with sexual function or causing frequent discomfort	Severe vaginal dryness resulting in dyspareunia or severe discomfort	-	-
Definition: A disorder characterized by an uncomfortable feeling of itching and burning in the vagina.					
Vaginal fistula	Asymptomatic clinical or diagnostic observations only; intervention not indicated	Symptomatic and intervention not indicated	Severe symptoms; elective operative intervention indicated	Life-threatening consequences; urgent intervention indicated	Death
Definition: A disorder characterized by an abnormal communication between the vagina and another organ or anatomic site.					
Vaginal hemorrhage	Minimal bleeding identified on clinical exam or imaging study; intervention not indicated	Moderate bleeding; medical intervention indicated	Severe bleeding; transfusion indicated; radiologic or endoscopic intervention indicated	Life-threatening consequences; urgent operative intervention indicated	Death
Definition: A disorder characterized by bleeding from the vagina.					
Vaginal inflammation	Mild discomfort or pain, edema, or redness	Moderate discomfort or pain, edema, or redness; limiting instrumental ADL	Severe discomfort or pain, edema, or redness; limiting self care ADL; small areas of mucosal ulceration	Widespread areas of mucosal ulceration; life-threatening consequences; urgent intervention indicated	Death
Definition: A disorder characterized by inflammation involving the vagina. Symptoms may include redness, edema, marked discomfort and an increase in vaginal discharge.					
Vaginal obstruction	Diagnostic observations only; intervention not indicated	Mild symptoms; elective intervention indicated	Severe symptoms; elective operative intervention indicated	-	-
Definition: A disorder characterized by blockage of vaginal canal.					

Reproductive system and breast disorders

	Grade				
**Adverse Event**	**1**	**2**	**3**	**4**	**5**
Vaginal pain	Mild pain	Moderate pain; limiting instrumental ADL	Severe pain; limiting self care ADL	-	-
Definition: A disorder characterized by a sensation of marked discomfort in the vagina.					
Vaginal perforation	Asymptomatic clinical or diagnostic observations only; intervention not indicated	Symptomatic and intervention not indicated	Severe symptoms; elective operative intervention indicated	Life-threatening consequences; urgent intervention indicated	Death
Definition: A disorder characterized by a rupture in the vaginal wall.					
Vaginal stricture	Asymptomatic; mild vaginal shortening or narrowing	Vaginal narrowing and/or shortening not interfering with physical examination	Vaginal narrowing and/or shortening interfering with the use of tampons, sexual activity or physical examination	-	Death
Definition: A disorder characterized by a narrowing of the vaginal canal.					
Vaginismus	Mild discomfort or pain associated with vaginal spasm/tightening; no impact upon sexual function or physical examination	Moderate discomfort or pain associated with vaginal spasm/tightening; disruption in sexual function and physical examination	Severe discomfort or pain associated with vaginal spasm/tightening; unable to tolerate vaginal penetration or physical examination	-	-
Definition: A disorder characterized by involuntary spasms of the pelvic floor muscles, resulting in pathologic tightness of the vaginal wall during penetration such as during sexual intercourse.					

Reproductive system and breast disorders

	Grade				
**Adverse Event**	**1**	**2**	**3**	**4**	**5**
Reproductive system and breast disorders - Other, specify	Asymptomatic or mild symptoms; clinical or diagnostic observations only; intervention not indicated	Moderate; minimal, local or noninvasive intervention indicated; limiting age-appropriate instrumental ADL	Severe or medically significant but not immediately life-threatening; hospitalization or prolongation of existing hospitalization indicated; disabling; limiting self care ADL	Life-threatening consequences; urgent intervention indicated	Death

Respiratory, thoracic and mediastinal disorders

	Grade				
**Adverse Event**	**1**	**2**	**3**	**4**	**5**
Adult respiratory distress syndrome	-	-	Present with radiologic findings; intubation not indicated	Life-threatening respiratory or hemodynamic compromise; intubation or urgent intervention indicated	Death
Definition: A disorder characterized by progressive and life-threatening pulmonary distress in the absence of an underlying pulmonary condition, usually following major trauma or surgery.					
Allergic rhinitis	Mild symptoms; intervention not indicated	Moderate symptoms; medical intervention indicated	-	-	-
Definition: A disorder characterized by an inflammation of the nasal mucous membranes caused by an IgE-mediated response to external allergens. The inflammation may also involve the mucous membranes of the sinuses, eyes, middle ear, and pharynx. Symptoms include sneezing, nasal congestion, rhinorrhea and itching.					
Apnea	-	-	Present; medical intervention indicated	Life-threatening respiratory or hemodynamic compromise; intubation or urgent intervention indicated	Death
Definition: A disorder characterized by cessation of breathing.					
Aspiration	Asymptomatic; clinical or diagnostic observations only; intervention not indicated	Altered eating habits; coughing or choking episodes after eating or swallowing; medical intervention indicated (e.g., suction or oxygen)	Dyspnea and pneumonia symptoms (e.g., aspiration pneumonia); hospitalization indicated; unable to aliment orally	Life-threatening respiratory or hemodynamic compromise; intubation or urgent intervention indicated	Death
Definition: A disorder characterized by inhalation of solids or liquids into the lungs.					

Respiratory, thoracic and mediastinal disorders

	Grade				
**Adverse Event**	**1**	**2**	**3**	**4**	**5**
Atelectasis	Asymptomatic; clinical or diagnostic observations only; intervention not indicated	Symptomatic (e.g., dyspnea, cough); medical intervention indicated (e.g., chest physiotherapy, suctioning); bronchoscopic suctioning	Oxygen indicated; hospitalization or elective operative intervention indicated (e.g., stent, laser)	Life-threatening respiratory or hemodynamic compromise; intubation or urgent intervention indicated	Death
Definition: A disorder characterized by the collapse of part or the entire lung.					
Bronchial fistula	Asymptomatic; clinical or diagnostic observations only; intervention not indicated	Symptomatic; tube thoracostomy or medical management indicated; limiting instrumental ADL	Severe symptoms; limiting self care ADL; endoscopic or operative intervention indicated (e.g., stent or primary closure)	Life-threatening consequences; urgent operative intervention with thoracoplasty, chronic open drainage or multiple thoracotomies indicated	Death
Definition: A disorder characterized by an abnormal communication between the bronchus and another organ or anatomic site.					
Bronchial obstruction	Asymptomatic; clinical or diagnostic observations only; intervention not indicated	Symptomatic (e.g., mild wheezing); endoscopic evaluation indicated; radiographic evidence of atelectasis/lobar collapse; medical management indicated (e.g., steroids, bronchodilators)	Shortness of breath with stridor; endoscopic intervention indicated (e.g., laser, stent placement)	Life-threatening respiratory or hemodynamic compromise; intubation or urgent intervention indicated	Death
Definition: A disorder characterized by blockage of a bronchus passage, most often by bronchial secretions and exudates.					

Respiratory, thoracic and mediastinal disorders

	Grade				
**Adverse Event**	**1**	**2**	**3**	**4**	**5**
Bronchial stricture	Asymptomatic; clinical or diagnostic observations only; intervention not indicated	Symptomatic (e.g., rhonchi or wheezing) but without respiratory distress; medical intervention indicated (e.g., steroids, bronchodilators)	Shortness of breath with stridor; endoscopic intervention indicated (e.g., laser, stent placement)	Life-threatening respiratory or hemodynamic compromise; intubation or urgent intervention indicated	Death
Definition: A disorder characterized by a narrowing of the bronchial tube.					
Bronchopleural fistula	Asymptomatic; clinical or diagnostic observations only; intervention not indicated	Symptomatic; tube thoracostomy or medical intervention indicated; limiting instrumental ADL	Severe symptoms; limiting self care ADL; endoscopic or operative intervention indicated (e.g., stent or primary closure)	Life-threatening consequences; urgent operative intervention with thoracoplasty, chronic open drainage or multiple thoracotomies indicated	Death
Definition: A disorder characterized by an abnormal communication between a bronchus and the pleural cavity.					
Bronchopulmonary hemorrhage	Mild symptoms; intervention not indicated	Moderate symptoms; medical intervention indicated	Transfusion, radiologic, endoscopic, or operative intervention indicated (e.g., hemostasis of bleeding site)	Life-threatening respiratory or hemodynamic compromise; intubation or urgent intervention indicated	Death
Definition: A disorder characterized by bleeding from the bronchial wall and/or lung parenchyma.					
Bronchospasm	Mild symptoms; intervention not indicated	Symptomatic; medical intervention indicated; limiting instrumental ADL	Limiting self care ADL; oxygen saturation decreased	Life-threatening respiratory or hemodynamic compromise; intubation or urgent intervention indicated	Death
Definition: A disorder characterized by a sudden contraction of the smooth muscles of the bronchial wall.					

Respiratory, thoracic and mediastinal disorders

	Grade				
**Adverse Event**	**1**	**2**	**3**	**4**	**5**
Chylothorax	Asymptomatic; clinical or diagnostic observations only; intervention not indicated	Symptomatic; thoracentesis or tube drainage indicated	Severe symptoms; elective operative intervention indicated	Life-threatening respiratory or hemodynamic compromise; intubation or urgent intervention indicated	Death
Definition: A disorder characterized by milky pleural effusion (abnormal collection of fluid) resulting from accumulation of lymph fluid in the pleural cavity.					
Cough	Mild symptoms; nonprescription intervention indicated	Moderate symptoms, medical intervention indicated; limiting instrumental ADL	Severe symptoms; limiting self care ADL	-	-
Definition: A disorder characterized by sudden, often repetitive, spasmodic contraction of the thoracic cavity, resulting in violent release of air from the lungs and usually accompanied by a distinctive sound.					
Dyspnea	Shortness of breath with moderate exertion	Shortness of breath with minimal exertion; limiting instrumental ADL	Shortness of breath at rest; limiting self care ADL	Life-threatening consequences; urgent intervention indicated	Death
Definition: A disorder characterized by an uncomfortable sensation of difficulty breathing.					
Epistaxis	Mild symptoms; intervention not indicated	Moderate symptoms; medical intervention indicated (e.g., nasal packing, cauterization; topical vasoconstrictors)	Transfusion, radiologic, endoscopic, or operative intervention indicated (e.g., hemostasis of bleeding site)	Life-threatening consequences; urgent intervention indicated	Death
Definition: A disorder characterized by bleeding from the nose.					
Hiccups	Mild symptoms; intervention not indicated	Moderate symptoms; medical intervention indicated; limiting instrumental ADL	Severe symptoms; interfering with sleep; limiting self care ADL	-	-
Definition: A disorder characterized by repeated gulp sounds that result from an involuntary opening and closing of the glottis. This is attributed to a spasm of the diaphragm.					

Respiratory, thoracic and mediastinal disorders

	Grade				
**Adverse Event**	**1**	**2**	**3**	**4**	**5**
Hoarseness	Mild or intermittent voice change; fully understandable; self-resolves	Moderate or persistent voice changes; may require occasional repetition but understandable on telephone; medical evaluation indicated	Severe voice changes including predominantly whispered speech	-	-
Definition: A disorder characterized by harsh and raspy voice arising from or spreading to the larynx.					
Hypoxia	-	Decreased oxygen saturation with exercise (e.g., pulse oximeter <88%); intermittent supplemental oxygen	Decreased oxygen saturation at rest (e.g., pulse oximeter <88% or PaO2 <=55 mm Hg)	Life-threatening airway compromise; urgent intervention indicated (e.g., tracheotomy or intubation)	Death
Definition: A disorder characterized by a decrease in the level of oxygen in the body.					
Laryngeal edema	Asymptomatic; clinical or diagnostic observations only; intervention not indicated	Symptomatic; medical intervention indicated (e.g., dexamethasone, epinephrine, antihistamines)	Stridor; respiratory distress; hospitalization indicated	Life-threatening airway compromise; urgent intervention indicated (e.g., tracheotomy or intubation)	Death
Definition: A disorder characterized by swelling due to an excessive accumulation of fluid in the larynx.					
Laryngeal fistula	Asymptomatic; clinical or diagnostic observations only; intervention not indicated	Symptomatic; tube thoracostomy or medical management indicated; limiting instrumental ADL	Severe symptoms; limiting self care ADL; endoscopic or operative intervention indicated (e.g., stent or primary closure)	Life-threatening consequences; urgent operative intervention indicated (e.g., thoracoplasty, chronic open drainage or multiple thoracotomies)	Death
Definition: A disorder characterized by an abnormal communication between the larynx and another organ or anatomic site.					

Respiratory, thoracic and mediastinal disorders

	Grade				
**Adverse Event**	**1**	**2**	**3**	**4**	**5**
Laryngeal hemorrhage	Mild cough or trace hemoptysis; laryngoscopic findings	Moderate symptoms; medical intervention indicated	Transfusion, radiologic, endoscopic, or operative intervention indicated (e.g., hemostasis of bleeding site)	Life-threatening airway compromise; urgent intervention indicated (e.g., tracheotomy or intubation)	Death
Definition: A disorder characterized by bleeding from the larynx.					
Laryngeal inflammation	Mild sore throat; raspy voice	Moderate sore throat; analgesics indicated	Severe throat pain; endoscopic intervention indicated	-	-
Definition: A disorder characterized by an inflammation involving the larynx.					
Laryngeal mucositis	Endoscopic findings only; mild discomfort with normal intake	Moderate discomfort; altered oral intake	Severe pain; severely altered eating/swallowing; medical intervention indicated	Life-threatening airway compromise; urgent intervention indicated (e.g., tracheotomy or intubation)	Death
Definition: A disorder characterized by an inflammation involving the mucous membrane of the larynx.					
Laryngeal obstruction	Asymptomatic; clinical or diagnostic observations only; intervention not indicated	Symptomatic (e.g., noisy airway breathing), but causing no respiratory distress; medical management indicated (e.g., steroids); limiting instrumental ADL	Limiting self care ADL; stridor; endoscopic intervention indicated (e.g., stent, laser)	Life-threatening consequences; urgent intervention indicated	Death
Definition: A disorder characterized by blockage of the laryngeal airway.					

Respiratory, thoracic and mediastinal disorders

	Grade				
**Adverse Event**	**1**	**2**	**3**	**4**	**5**
Laryngeal stenosis	Asymptomatic; clinical or diagnostic observations only; intervention not indicated	Symptomatic (e.g., noisy airway breathing), but causing no respiratory distress; medical management indicated (e.g., steroids)	Limiting self care ADL; stridor; endoscopic intervention indicated (e.g., stent, laser)	Life-threatening consequences; urgent intervention indicated	Death
Definition: A disorder characterized by a narrowing of the laryngeal airway.					
Laryngopharyngeal dysesthesia	Mild symptoms; no anxiety; intervention not indicated	Moderate symptoms; mild anxiety, but no dyspnea; short duration of observation and or anxiolytic indicated; limiting instrumental ADL	Severe symptoms; dyspnea and swallowing difficulty; limiting self care ADL	Life-threatening consequences	Death
Definition: A disorder characterized by an uncomfortable persistent sensation in the area of the laryngopharynx.					
Laryngospasm	-	Transient episode; intervention not indicated	Recurrent episodes; noninvasive intervention indicated (e.g., breathing technique, pressure point massage)	Persistent or severe episodes associated with syncope; urgent intervention indicated (e.g., fiberoptic laryngoscopy, intubation, botox injection)	Death
Definition: A disorder characterized by paroxysmal spasmodic muscular contraction of the vocal cords.					
Mediastinal hemorrhage	Radiologic evidence only; minimal symptoms; intervention not indicated	Moderate symptoms; medical intervention indicated	Transfusion, radiologic, endoscopic, or elective operative intervention indicated (e.g., hemostasis of bleeding site)	Life-threatening consequences; urgent intervention indicated	Death

Respiratory, thoracic and mediastinal disorders

	Grade				
**Adverse Event**	**1**	**2**	**3**	**4**	**5**
Definition: A disorder characterized by bleeding from the mediastinum.					
Nasal congestion	Mild symptoms; intervention not indicated	Moderate symptoms; medical intervention indicated	Associated with bloody nasal discharge or epistaxis	-	-
Definition: A disorder characterized by obstruction of the nasal passage due to mucosal edema.					
Pharyngeal fistula	Asymptomatic; clinical or diagnostic observations only; intervention not indicated	Symptomatic; tube thoracostomy or medical intervention indicated; limiting instrumental ADL	Severe symptoms; limiting self care ADL; endoscopic or operative intervention indicated (e.g., stent or primary closure)	Life-threatening consequences; urgent intervention indicated	Death
Definition: A disorder characterized by an abnormal communication between the pharynx and another organ or anatomic site.					
Pharyngeal hemorrhage	Mild symptoms; intervention not indicated	Moderate symptoms; medical intervention indicated	Transfusion, radiologic, endoscopic, or operative intervention indicated (e.g., hemostasis of bleeding site)	Life-threatening respiratory or hemodynamic compromise; intubation or urgent intervention indicated	Death
Definition: A disorder characterized by bleeding from the pharynx.					
Pharyngeal mucositis	Endoscopic findings only; minimal symptoms with normal oral intake; mild pain but analgesics not indicated	Moderate pain and analgesics indicated; altered oral intake; limiting instrumental ADL	Severe pain; unable to adequately aliment or hydrate orally; limiting self care ADL	Life-threatening consequences; urgent intervention indicated	Death
Definition: A disorder characterized by an inflammation involving the mucous membrane of the pharynx.					

Respiratory, thoracic and mediastinal disorders

	Grade				
**Adverse Event**	**1**	**2**	**3**	**4**	**5**
Pharyngeal necrosis	-	-	Inability to aliment adequately by GI tract; tube feeding or TPN indicated; radiologic, endoscopic, or operative intervention indicated	Life-threatening consequences; urgent operative intervention indicated	Death
Definition: A disorder characterized by a necrotic process occurring in the pharynx.					
Pharyngeal stenosis	Asymptomatic; clinical or diagnostic observations only; intervention not indicated	Symptomatic (e.g., noisy airway breathing), but causing no respiratory distress; medical management indicated (e.g., steroids); limiting instrumental ADL	Limiting self care ADL; stridor; endoscopic intervention indicated (e.g., stent, laser)	Life-threatening airway compromise; urgent intervention indicated (e.g., tracheotomy or intubation)	Death
Definition: A disorder characterized by a narrowing of the pharyngeal airway.					
Pharyngolaryngeal pain	Mild pain	Moderate pain; limiting instrumental ADL	Severe pain; limiting self care ADL	-	-
Definition: A disorder characterized by marked discomfort sensation in the pharyngolaryngeal region.					
Pleural effusion	Asymptomatic; clinical or diagnostic observations only; intervention not indicated	Symptomatic; intervention indicated (e.g., diuretics or limited therapeutic thoracentesis)	Symptomatic with respiratory distress and hypoxia; surgical intervention including chest tube or pleurodesis indicated	Life-threatening respiratory or hemodynamic compromise; intubation or urgent intervention indicated	Death
Definition: A disorder characterized by an increase in amounts of fluid within the pleural cavity. Symptoms include shortness of breath, cough and marked chest discomfort.					

Respiratory, thoracic and mediastinal disorders

	Grade				
**Adverse Event**	**1**	**2**	**3**	**4**	**5**
Pleural hemorrhage	Asymptomatic; mild hemorrhage confirmed by thoracentesis	Symptomatic or associated with pneumothorax; chest tube drainage indicated	>1000 ml of blood evacuated; persistent bleeding (150-200 ml/hr for 2 - 4 hr); persistent transfusion indicated; elective operative intervention indicated	Life-threatening respiratory or hemodynamic compromise; intubation or urgent intervention indicated	Death
Definition: A disorder characterized by bleeding from the pleural cavity.					
Pleuritic pain	Mild pain	Moderate pain; limiting instrumental ADL	Severe pain; limiting self care ADL	-	-
Definition: A disorder characterized by marked discomfort sensation in the pleura.					
Pneumonitis	Asymptomatic; clinical or diagnostic observations only; intervention not indicated	Symptomatic; medical intervention indicated; limiting instrumental ADL	Severe symptoms; limiting self care ADL; oxygen indicated	Life-threatening respiratory compromise; urgent intervention indicated (e.g., tracheotomy or intubation)	Death
Definition: A disorder characterized by inflammation focally or diffusely affecting the lung parenchyma.					
Pneumothorax	Asymptomatic; clinical or diagnostic observations only; intervention not indicated	Symptomatic; intervention indicated (e.g., tube placement without sclerosis)	Sclerosis and/or operative intervention indicated; hospitalization indicated	Life-threatening consequences; urgent intervention indicated	Death
Definition: A disorder characterized by abnormal presence of air in the pleural cavity resulting in the collapse of the lung.					
Postnasal drip	Mild symptoms; intervention not indicated	Moderate symptoms; medical intervention indicated	-	-	-
Definition: A disorder characterized by excessive mucous secretion in the back of the nasal cavity or throat, causing sore throat and/or coughing.					

Respiratory, thoracic and mediastinal disorders

	Grade				
**Adverse Event**	**1**	**2**	**3**	**4**	**5**
Productive cough	Occasional/minimal production of sputum with cough	Moderate sputum production; limiting instrumental ADL	Persistent or copious production of sputum; limiting self care ADL	-	-
Definition: A disorder characterized by expectorated secretions upon coughing.					
Pulmonary edema	Radiologic findings only; minimal dyspnea on exertion	Moderate dyspnea on exertion; medical intervention indicated; limiting instrumental ADL	Severe dyspnea or dyspnea at rest; oxygen indicated; limiting self care ADL	Life-threatening respiratory compromise; urgent intervention or intubation with ventilatory support indicated	Death
Definition: A disorder characterized by accumulation of fluid in the lung tissues that causes a disturbance of the gas exchange that may lead to respiratory failure.					
Pulmonary fibrosis	Mild hypoxemia; radiologic pulmonary fibrosis <25% of lung volume	Moderate hypoxemia; evidence of pulmonary hypertension; radiographic pulmonary fibrosis 25 - 50%	Severe hypoxemia; evidence of right-sided heart failure; radiographic pulmonary fibrosis >50 - 75%	Life-threatening consequences (e.g., hemodynamic/pulmonary complications); intubation with ventilatory support indicated; radiographic pulmonary fibrosis >75% with severe honeycombing	Death
Definition: A disorder characterized by the replacement of the lung tissue by connective tissue, leading to progressive dyspnea, respiratory failure or right heart failure.					
Pulmonary fistula	Asymptomatic; clinical or diagnostic observations only; intervention not indicated	Symptomatic; tube thoracostomy or medical management indicated; limiting instrumental ADL	Limiting self care ADL; endoscopic stenting or operative intervention indicated	Life-threatening consequences; urgent operative intervention indicated	Death
Definition: A disorder characterized by an abnormal communication between the lung and another organ or anatomic site.					

Respiratory, thoracic and mediastinal disorders

	Grade				
**Adverse Event**	**1**	**2**	**3**	**4**	**5**
Pulmonary hypertension	Minimal dyspnea; findings on physical exam or other evaluation	Moderate dyspnea, cough; requiring evaluation by cardiac catheterization and medical intervention	Severe symptoms, associated with hypoxemia, right heart failure; oxygen indicated	Life-threatening airway consequences; urgent intervention indicated (e.g., tracheotomy or intubation)	Death
Definition: A disorder characterized by an increase in pressure within the pulmonary circulation due to lung or heart disorder.					
Respiratory failure	-	-	-	Life-threatening consequences; urgent intervention, intubation, or ventilatory support indicated	Death
Definition: A disorder characterized by impaired gas exchange by the respiratory system resulting in hypoxemia and a decrease in oxygenation of the tissues that may be associated with an increase in arterial levels of carbon dioxide.					
Retinoic acid syndrome	Fluid retention; <3 kg of weight gain; intervention with fluid restriction and/or diuretics indicated	Moderate signs or symptoms; steroids indicated	Severe symptoms; hospitalization indicated	Life-threatening consequences; ventilatory support indicated	Death
Definition: A disorder characterized by weight gain, dyspnea, pleural and pericardial effusions, leukocytosis and/or renal failure originally described in patients treated with all-trans retinoic acid.					
Sinus disorder	Asymptomatic mucosal crusting; blood-tinged secretions	Symptomatic stenosis or edema/narrowing interfering with airflow; limiting instrumental ADL	Stenosis with significant nasal obstruction; limiting self care ADL	Necrosis of soft tissue or bone; urgent operative intervention indicated	Death
Definition: A disorder characterized by involvement of the paranasal sinuses.					

Respiratory, thoracic and mediastinal disorders

	Grade				
**Adverse Event**	**1**	**2**	**3**	**4**	**5**
Sleep apnea	Snoring and nocturnal sleep arousal without apneic periods	Moderate apnea and oxygen desaturation; excessive daytime sleepiness; medical evaluation indicated; limiting instrumental ADL	Oxygen desaturation; associated with hypertension; medical intervention indicated; limiting self care ADL	Cardiovascular or neuropsychiatric symptoms; urgent operative intervention indicated	Death
Definition: A disorder characterized by cessation of breathing for short periods during sleep.					
Sneezing	Mild symptoms; intervention not indicated	Moderate symptoms; medical intervention indicated	-	-	-
Definition: A disorder characterized by the involuntary expulsion of air from the nose.					
Sore throat	Mild pain	Moderate pain; limiting instrumental ADL	Severe pain; limiting self care ADL; limiting ability to swallow	-	-
Definition: A disorder characterized by of marked discomfort in the throat					
Stridor	-	-	Respiratory distress limiting self care ADL; medical intervention indicated	Life-threatening airway compromise; urgent intervention indicated (e.g., tracheotomy or intubation)	Death
Definition: A disorder characterized by a high pitched breathing sound due to laryngeal or upper airway obstruction.					
Tracheal fistula	Asymptomatic; clinical or diagnostic observations only; intervention not indicated	Symptomatic; tube thoracostomy or medical intervention indicated; limiting instrumental ADL	Severe symptoms; limiting self care ADL; endoscopic or operative intervention indicated (e.g., stent or primary closure)	Life-threatening consequences; urgent operative intervention indicated (e.g., thoracoplasty, chronic open drainage or multiple thoracotomies)	Death

Respiratory, thoracic and mediastinal disorders

	Grade				
**Adverse Event**	**1**	**2**	**3**	**4**	**5**
Definition: A disorder characterized by an abnormal communication between the trachea and another organ or anatomic site.					
Tracheal mucositis	Endoscopic findings only; minimal hemoptysis, pain, or respiratory symptoms	Moderate symptoms; medical intervention indicated; limiting instrumental ADL	Severe pain; hemorrhage or respiratory symptoms; limiting self care ADL	Life-threatening consequences; urgent intervention indicated	Death
Definition: A disorder characterized by an inflammation involving the mucous membrane of the trachea.					
Tracheal stenosis	Asymptomatic; clinical or diagnostic observations only; intervention not indicated	Symptomatic (e.g., noisy airway breathing), but causing no respiratory distress; medical management indicated (e.g., steroids)	Stridor or respiratory distress limiting self care ADL; endoscopic intervention indicated (e.g., stent, laser)	Life-threatening airway compromise; urgent intervention indicated (e.g., tracheotomy or intubation)	Death
Definition: A disorder characterized by a narrowing of the trachea.					
Voice alteration	Mild or intermittent change from normal voice	Moderate or persistent change from normal voice; still understandable	Severe voice changes including predominantly whispered speech; may require frequent repetition or face-to-face contact for understandability; may require assistive technology	-	-
Definition: A disorder characterized by a change in the sound and/or speed of the voice.					
Wheezing	Detectable airway noise with minimal symptoms	Moderate symptoms; medical intervention indicated; limiting instrumental ADL	Severe respiratory symptoms limiting self care ADL; oxygen therapy or hospitalization indicated	Life-threatening consequences; urgent intervention indicated	Death

Respiratory, thoracic and mediastinal disorders					
	Grade				
**Adverse Event**	**1**	**2**	**3**	**4**	**5**
Definition: A disorder characterized by a high-pitched, whistling sound during breathing. It results from the narrowing or obstruction of the respiratory airways.					
Respiratory, thoracic and mediastinal disorders - Other, specify	Asymptomatic or mild symptoms; clinical or diagnostic observations only; intervention not indicated	Moderate; minimal, local or noninvasive intervention indicated; limiting age-appropriate instrumental ADL	Severe or medically significant but not immediately life-threatening; hospitalization or prolongation of existing hospitalization indicated; disabling; limiting self care ADL	Life-threatening consequences; urgent intervention indicated	Death

Skin and subcutaneous tissue disorders

	Grade				
**Adverse Event**	**1**	**2**	**3**	**4**	**5**
Alopecia	Hair loss of <50% of normal for that individual that is not obvious from a distance but only on close inspection; a different hair style may be required to cover the hair loss but it does not require a wig or hair piece to camouflage	Hair loss of >=50% normal for that individual that is readily apparent to others; a wig or hair piece is necessary if the patient desires to completely camouflage the hair loss; associated with psychosocial impact	-	-	-
Definition: A disorder characterized by a decrease in density of hair compared to normal for a given individual at a given age and body location.					
Body odor	Mild odor; physician intervention not indicated; self care interventions	Pronounced odor; psychosocial impact; patient seeks medical intervention	-	-	-
Definition: A disorder characterized by an abnormal body smell resulting from the growth of bacteria on the body.					
Bullous dermatitis	Asymptomatic; blisters covering <10% BSA	Blisters covering 10 - 30% BSA; painful blisters; limiting instrumental ADL	Blisters covering >30% BSA; limiting self care ADL	Blisters covering >30% BSA; associated with fluid or electrolyte abnormalities; ICU care or burn unit indicated	Death
Definition: A disorder characterized by inflammation of the skin characterized by the presence of bullae which are filled with fluid.					
Dry skin	Covering <10% BSA and no associated erythema or pruritus	Covering 10 - 30% BSA and associated with erythema or pruritus; limiting instrumental ADL	Covering >30% BSA and associated with pruritus; limiting self care ADL	-	-
Definition: A disorder characterized by flaky and dull skin; the pores are generally fine, the texture is a papery thin texture.					

Skin and subcutaneous tissue disorders

	Grade				
**Adverse Event**	**1**	**2**	**3**	**4**	**5**
Erythema multiforme	Target lesions covering <10% BSA and not associated with skin tenderness	Target lesions covering 10 - 30% BSA and associated with skin tenderness	Target lesions covering >30% BSA and associated with oral or genital erosions	Target lesions covering >30% BSA; associated with fluid or electrolyte abnormalities; ICU care or burn unit indicated	Death
Definition: A disorder characterized by target lesions (a pink-red ring around a pale center).					
Erythroderma	-	Erythema covering >90% BSA without associated symptoms; limiting instrumental ADL	Erythema covering >90% BSA with associated symptoms (e.g., pruritus or tenderness); limiting self care ADL	Erythema covering >90% BSA with associated fluid or electrolyte abnormalities; ICU care or burn unit indicated	Death
Definition: A disorder characterized by generalized inflammatory erythema and exfoliation. The inflammatory process involves > 90% of the body surface area.					
Fat atrophy	Covering <10% BSA and asymptomatic	Covering 10 - 30% BSA and associated with erythema or tenderness; limiting instrumental ADL	Covering >30% BSA; associated with erythema or tenderness; limiting self-care ADL	-	-
Definition: A disorder characterized by shrinking of adipose tissue.					
Hirsutism	In women, increase in length, thickness or density of hair in a male distribution that the patient is able to camouflage by periodic shaving, bleaching, or removal of hair	In women, increase in length, thickness or density of hair in a male distribution that requires daily shaving or consistent destructive means of hair removal to camouflage; associated with psychosocial impact	-	-	-

Skin and subcutaneous tissue disorders					
	Grade				
**Adverse Event**	**1**	**2**	**3**	**4**	**5**
Definition: A disorder characterized by the presence of excess hair growth in women in anatomic sites where growth is considered to be a secondary male characteristic and under androgen control (beard, moustache, chest, abdomen)					
Hyperhidrosis	Limited to one site (palms, soles, or axillae); self care interventions	Involving >1 site; patient seeks medical intervention; associated with psychosocial impact	Generalized involving sites other than palms, soles, or axillae; associated with electrolyte/hemodynamic imbalance	-	-
Definition: A disorder characterized by excessive perspiration.					
Hypertrichosis	Increase in length, thickness or density of hair that the patient is either able to camouflage by periodic shaving or removal of hairs or is not concerned enough about the overgrowth to use any form of hair removal	Increase in length, thickness or density of hair at least on the usual exposed areas of the body [face (not limited to beard/moustache area) plus/minus arms] that requires frequent shaving or use of destructive means of hair removal to camouflage; associated with psychosocial impact	-	-	-
Definition: A disorder characterized by hair density or length beyond the accepted limits of normal in a particular body region, for a particular age or race.					
Hypohidrosis	-	Symptomatic; limiting instrumental ADL	Increase in body temperature; limiting self care ADL	Heat stroke	Death
Definition: A disorder characterized by reduced sweating.					

Skin and subcutaneous tissue disorders

	Grade				
**Adverse Event**	**1**	**2**	**3**	**4**	**5**
Lipohypertrophy	Asymptomatic and covering <10% BSA	Covering 10 - 30% BSA and associated tenderness; limiting instrumental ADL	Covering >30% BSA and associated tenderness and narcotics or NSAIDs indicated; lipohypertrophy; limiting self care ADL	-	-
Definition: A disorder characterized by hypertrophy of the subcutaneous adipose tissue at the site of multiple subcutaneous injections of insulin.					
Nail discoloration	Asymptomatic; clinical or diagnostic observations only; intervention not indicated	-	-	-	-
Definition: A disorder characterized by a change in the color of the nail plate.					
Nail loss	Asymptomatic separation of the nail bed from the nail plate or nail loss	Symptomatic separation of the nail bed from the nail plate or nail loss; limiting instrumental ADL	-	-	-
Definition: A disorder characterized by loss of all or a portion of the nail.					
Nail ridging	Asymptomatic; clinical or diagnostic observations only; intervention not indicated	-	-	-	-
Definition: A disorder characterized by vertical or horizontal ridges on the nails.					
Pain of skin	Mild pain	Moderate pain; limiting instrumental ADL	Severe pain; limiting self care ADL	-	-
Definition: A disorder characterized by marked discomfort sensation in the skin.					

Skin and subcutaneous tissue disorders

	Grade				
**Adverse Event**	**1**	**2**	**3**	**4**	**5**
Palmar-plantar erythrodysesthesia syndrome	Minimal skin changes or dermatitis (e.g., erythema, edema, or hyperkeratosis) without pain	Skin changes (e.g., peeling, blisters, bleeding, edema, or hyperkeratosis) with pain; limiting instrumental ADL	Severe skin changes (e.g., peeling, blisters, bleeding, edema, or hyperkeratosis) with pain; limiting self care ADL	-	-
Definition: A disorder characterized by redness, marked discomfort, swelling, and tingling in the palms of the hands or the soles of the feet.					
Periorbital edema	Soft or non-pitting	Indurated or pitting edema; topical intervention indicated	Edema associated with visual disturbance; increased intraocular pressure, glaucoma or retinal hemorrhage; optic neuritis; diuretics indicated; operative intervention indicated	-	-
Definition: A disorder characterized by swelling due to an excessive accumulation of fluid around the orbits of the face.					
Photosensitivity	Painless erythema and erythema covering <10% BSA	Tender erythema covering 10 - 30% BSA	Erythema covering >30% BSA and erythema with blistering; photosensitivity; oral corticosteroid therapy indicated; pain control indicated (e.g., narcotics or NSAIDs)	Life-threatening consequences; urgent intervention indicated	Death
Definition: A disorder characterized by an increase in sensitivity of the skin to light.					

Skin and subcutaneous tissue disorders

	Grade				
**Adverse Event**	**1**	**2**	**3**	**4**	**5**
Pruritus	Mild or localized; topical intervention indicated	Intense or widespread; intermittent; skin changes from scratching (e.g., edema, papulation, excoriations, lichenification, oozing/crusts); oral intervention indicated; limiting instrumental ADL	Intense or widespread; constant; limiting self care ADL or sleep; oral corticosteroid or immunosuppressive therapy indicated	-	-
Definition: A disorder characterized by an intense itching sensation.					
Purpura	Combined area of lesions covering <10% BSA	Combined area of lesions covering 10 - 30% BSA; bleeding with trauma	Combined area of lesions covering >30% BSA; spontaneous bleeding	-	-
Definition: A disorder characterized by hemorrhagic areas of the skin and mucous membrane. Newer lesions appear reddish in color. Older lesions are usually a darker purple color and eventually become a brownish-yellow color.					
Rash acneiform	Papules and/or pustules covering <10% BSA, which may or may not be associated with symptoms of pruritus or tenderness	Papules and/or pustules covering 10 - 30% BSA, which may or may not be associated with symptoms of pruritus or tenderness; associated with psychosocial impact; limiting instrumental ADL	Papules and/or pustules covering >30% BSA, which may or may not be associated with symptoms of pruritus or tenderness; limiting self care ADL; associated with local superinfection with oral antibiotics indicated	Papules and/or pustules covering any % BSA, which may or may not be associated with symptoms of pruritus or tenderness and are associated with extensive superinfection with IV antibiotics indicated; life-threatening consequences	Death
Definition: A disorder characterized by an eruption of papules and pustules, typically appearing in face, scalp, upper chest and back.					

Skin and subcutaneous tissue disorders

	Grade				
**Adverse Event**	**1**	**2**	**3**	**4**	**5**
Rash maculo-papular	Macules/papules covering <10% BSA with or without symptoms (e.g., pruritus, burning, tightness)	Macules/papules covering 10 - 30% BSA with or without symptoms (e.g., pruritus, burning, tightness); limiting instrumental ADL	Macules/papules covering >30% BSA with or without associated symptoms; limiting self care ADL	-	-
Definition: A disorder characterized by the presence of macules (flat) and papules (elevated). Also known as morbillform rash, it is one of the most common cutaneous adverse events, frequently affecting the upper trunk, spreading centripetally and associated with pruritus.					
Scalp pain	Mild pain	Moderate pain; limiting instrumental ADL	Severe pain; limiting self care ADL	-	-
Definition: A disorder characterized by marked discomfort sensation in the skin covering the top and the back of the head.					
Skin atrophy	Covering <10% BSA; associated with telangiectasias or changes in skin color	Covering 10 - 30% BSA; associated with striae or adnexal structure loss	Covering >30% BSA; associated with ulceration	-	-
Definition: A disorder characterized by the degeneration and thinning of the epidermis and dermis.					
Skin hyperpigmentation	Hyperpigmentation covering <10% BSA; no psychosocial impact	Hyperpigmentation covering >10% BSA; associated psychosocial impact	-	-	-
Definition: A disorder characterized by darkening of the skin due to excessive melanin deposition.					
Skin hypopigmentation	Hypopigmentation or depigmentation covering <10% BSA; no psychosocial impact	Hypopigmentation or depigmentation covering >10% BSA; associated psychosocial impact	-	-	-

Skin and subcutaneous tissue disorders

	Grade				
**Adverse Event**	**1**	**2**	**3**	**4**	**5**
Definition: A disorder characterized by loss of skin pigment.					
Skin induration	Mild induration, able to move skin parallel to plane (sliding) and perpendicular to skin (pinching up)	Moderate induration, able to slide skin, unable to pinch skin; limiting instrumental ADL	Severe induration, unable to slide or pinch skin; limiting joint movement or orifice (e.g., mouth, anus); limiting self care ADL	Generalized; associated with signs or symptoms of impaired breathing or feeding	Death
Definition: A disorder characterized by an area of hardness in the skin.					
Skin ulceration	Combined area of ulcers <1 cm; nonblanchable erythema of intact skin with associated warmth or edema	Combined area of ulcers 1 - 2 cm; partial thickness skin loss involving skin or subcutaneous fat	Combined area of ulcers >2 cm; full-thickness skin loss involving damage to or necrosis of subcutaneous tissue that may extend down to fascia	Any size ulcer with extensive destruction, tissue necrosis, or damage to muscle, bone, or supporting structures with or without full thickness skin loss	Death
Definition: A disorder characterized by circumscribed, inflammatory and necrotic erosive lesion on the skin.					
Stevens-Johnson syndrome	-	-	Skin sloughing covering <10% BSA with associated signs (e.g., erythema, purpura, epidermal detachment and mucous membrane detachment)	Skin sloughing covering 10 - 30% BSA with associated signs (e.g., erythema, purpura, epidermal detachment and mucous membrane detachment)	Death
Definition: A disorder characterized by less than 10% total body skin area separation of dermis. The syndrome is thought to be a hypersensitivity complex affecting the skin and the mucous membranes.					

Skin and subcutaneous tissue disorders

	Grade				
**Adverse Event**	**1**	**2**	**3**	**4**	**5**
Telangiectasia	Telangiectasias covering <10% BSA	Telangiectasias covering >10% BSA; associated with psychosocial impact	-	-	-
Definition: A disorder characterized by local dilatation of small vessels resulting in red discoloration of the skin or mucous membranes.					
Toxic epidermal necrolysis	-	-	-	Skin sloughing covering >=30% BSA with associated symptoms (e.g., erythema, purpura, or epidermal detachment)	Death
Definition: A disorder characterized by greater than 30% total body skin area separation of dermis. The syndrome is thought to be a hypersensitivity complex affecting the skin and the mucous membranes.					
Urticaria	Urticarial lesions covering <10% BSA; topical intervention indicated	Urticarial lesions covering 10 - 30% BSA; oral intervention indicated	Urticarial lesions covering >30% BSA; IV intervention indicated	-	-
Definition: A disorder characterized by an itchy skin eruption characterized by wheals with pale interiors and well-defined red margins.					
Skin and subcutaneous tissue disorders - Other, specify	Asymptomatic or mild symptoms; clinical or diagnostic observations only; intervention not indicated	Moderate; minimal, local or noninvasive intervention indicated; limiting age-appropriate instrumental ADL	Severe or medically significant but not immediately life-threatening; hospitalization or prolongation of existing hospitalization indicated; disabling; limiting self care ADL	Life-threatening consequences; urgent intervention indicated	Death

Social circumstances

	Grade				
**Adverse Event**	**1**	**2**	**3**	**4**	**5**
Menopause	Menopause occurring at age 46 - 53 years of age	Menopause occurring at age 40 - 45 years of age	Menopause occurring before age 40 years of age	-	-
Definition: A disorder characterized by the permanent cessation of menses, usually defined by 12 consecutive months of amenorrhea in a woman over 45 years of age.					
Social circumstances - Other, specify	Asymptomatic or mild symptoms; clinical or diagnostic observations only; intervention not indicated	Moderate; minimal, local or noninvasive intervention indicated; limiting age-appropriate instrumental ADL	Severe or medically significant but not immediately life-threatening; hospitalization or prolongation of existing hospitalization indicated; disabling; limiting self care ADL	Life-threatening consequences; urgent intervention indicated	Death

Surgical and medical procedures					
	Grade				
**Adverse Event**	**1**	**2**	**3**	**4**	**5**
Surgical and medical procedures - Other, specify	Asymptomatic or mild symptoms; clinical or diagnostic observations only; intervention not indicated	Moderate; minimal, local or noninvasive intervention indicated; limiting age-appropriate instrumental ADL	Severe or medically significant but not immediately life-threatening; hospitalization or prolongation of existing hospitalization indicated; disabling; limiting self care ADL	Life-threatening consequences; urgent intervention indicated	Death

Vascular disorders

	Grade				
**Adverse Event**	**1**	**2**	**3**	**4**	**5**
Capillary leak syndrome	-	Symptomatic; medical intervention indicated	Severe symptoms; intervention indicated	Life-threatening consequences; urgent intervention indicated	Death
Definition: A disorder characterized by leakage of intravascular fluids into the extravascular space. This syndrome is observed in patients who demonstrate a state of generalized leaky capillaries following shock syndromes, low-flow states, ischemia-reperfusion injuries, toxemias, medications, or poisoning. It can lead to generalized edema and multiple organ failure.					
Flushing	Asymptomatic; clinical or diagnostic observations only; intervention not indicated	Moderate symptoms; medical intervention indicated; limiting instrumental ADL	Symptomatic, associated with hypotension and/or tachycardia; limiting self care ADL	-	-
Definition: A disorder characterized by episodic reddening of the face.					
Hematoma	Mild symptoms; intervention not indicated	Minimally invasive evacuation or aspiration indicated	Transfusion, radiologic, endoscopic, or elective operative intervention indicated	Life-threatening consequences; urgent intervention indicated	Death
Definition: A disorder characterized by a localized collection of blood, usually clotted, in an organ, space, or tissue, due to a break in the wall of a blood vessel.					
Hot flashes	Mild symptoms; intervention not indicated	Moderate symptoms; limiting instrumental ADL	Severe symptoms; limiting self care ADL	-	-
Definition: A disorder characterized by an uncomfortable and temporary sensation of intense body warmth, flushing, sometimes accompanied by sweating upon cooling.					

Vascular disorders

	Grade				
**Adverse Event**	**1**	**2**	**3**	**4**	**5**
Hypertension	Prehypertension (systolic BP 120 - 139 mm Hg or diastolic BP 80 - 89 mm Hg)	Stage 1 hypertension (systolic BP 140 - 159 mm Hg or diastolic BP 90 - 99 mm Hg); medical intervention indicated; recurrent or persistent (>=24 hrs); symptomatic increase by >20 mm Hg (diastolic) or to >140/90 mm Hg if previously WNL; monotherapy indicated<br>Pediatric: recurrent or persistent (>=24 hrs) BP >ULN; monotherapy indicated	Stage 2 hypertension (systolic BP >=160 mm Hg or diastolic BP >=100 mm Hg); medical intervention indicated; more than one drug or more intensive therapy than previously used indicated<br>Pediatric: Same as adult	Life-threatening consequences (e.g., malignant hypertension, transient or permanent neurologic deficit, hypertensive crisis); urgent intervention indicated<br>Pediatric: Same as adult	Death
Definition: A disorder characterized by a pathological increase in blood pressure; a repeatedly elevation in the blood pressure exceeding 140 over 90 mm Hg.					
Hypotension	Asymptomatic, intervention not indicated	Non-urgent medical intervention indicated	Medical intervention or hospitalization indicated	Life-threatening and urgent intervention indicated	Death
Definition: A disorder characterized by a blood pressure that is below the normal expected for an individual in a given environment.					
Lymph leakage	-	Symptomatic; medical intervention indicated	Severe symptoms; radiologic, endoscopic or elective operative intervention indicated	Life-threatening consequences; urgent intervention indicated	Death
Definition: A disorder characterized by the loss of lymph fluid into the surrounding tissue or body cavity.					

Vascular disorders

	Grade				
**Adverse Event**	**1**	**2**	**3**	**4**	**5**
Lymphedema	Trace thickening or faint discoloration	Marked discoloration; leathery skin texture; papillary formation; limiting instrumental ADL	Severe symptoms; limiting self care ADL	-	-
Definition: A disorder characterized by excessive fluid collection in tissues that causes swelling.					
Lymphocele	Asymptomatic; clinical or diagnostic observations only; intervention not indicated	Symptomatic; medical intervention indicated	Severe symptoms; radiologic, endoscopic or elective operative intervention indicated	-	-
Definition: A disorder characterized by a cystic lesion containing lymph.					
Peripheral ischemia	-	Brief (<24 hrs) episode of ischemia managed non-surgically and without permanent deficit	Recurring or prolonged (>=24 hrs) and/or invasive intervention indicated	Life-threatening consequences; evidence of end organ damage; urgent operative intervention indicated	Death
Definition: A disorder characterized by impaired circulation to an extremity.					
Phlebitis	-	Present	-	-	-
Definition: A disorder characterized by inflammation of the wall of a vein.					
Superficial thrombophlebitis	-	Present	-	-	-
Definition: A disorder characterized by a blood clot and inflammation involving a superficial vein of the extremities.					

Vascular disorders

	Grade				
**Adverse Event**	**1**	**2**	**3**	**4**	**5**
Superior vena cava syndrome	Asymptomatic; incidental finding of SVC thrombosis	Symptomatic; medical intervention indicated (e.g., anticoagulation, radiation or chemotherapy)	Severe symptoms; multi-modality intervention indicated (e.g., anticoagulation, chemotherapy, radiation, stenting)	Life-threatening consequences; urgent multi-modality intervention indicated (e.g., lysis, thrombectomy, surgery)	Death
Definition: A disorder characterized by obstruction of the blood flow in the superior vena cava. Signs and symptoms include swelling and cyanosis of the face, neck, and upper arms, cough, orthopnea and headache.					
Thromboembolic event	Venous thrombosis (e.g., superficial thrombosis)	Venous thrombosis (e.g., uncomplicated deep vein thrombosis), medical intervention indicated	Thrombosis (e.g., uncomplicated pulmonary embolism [venous], non-embolic cardiac mural [arterial] thrombus), medical intervention indicated	Life-threatening (e.g., pulmonary embolism, cerebrovascular event, arterial insufficiency); hemodynamic or neurologic instability; urgent intervention indicated	Death
Definition: A disorder characterized by occlusion of a vessel by a thrombus that has migrated from a distal site via the blood stream.					
Vasculitis	Asymptomatic, intervention not indicated	Moderate symptoms, medical intervention indicated	Severe symptoms, medical intervention indicated (e.g., steroids)	Life-threatening; evidence of peripheral or visceral ischemia; urgent intervention indicated	Death
Definition: A disorder characterized by inflammation involving the wall of a vessel.					
Visceral arterial ischemia	-	Brief (<24 hrs) episode of ischemia managed medically and without permanent deficit	Prolonged (>=24 hrs) or recurring symptoms and/or invasive intervention indicated	Life-threatening consequences; evidence of end organ damage; urgent operative intervention indicated	Death

Vascular disorders

	Grade				
**Adverse Event**	**1**	**2**	**3**	**4**	**5**
Definition: A disorder characterized by a decrease in blood supply due to narrowing or blockage of a visceral (mesenteric) artery.					
Vascular disorders - Other, specify	Asymptomatic or mild symptoms; clinical or diagnostic observations only; intervention not indicated	Moderate; minimal, local or noninvasive intervention indicated; limiting age-appropriate instrumental ADL	Severe or medically significant but not immediately life-threatening; hospitalization or prolongation of existing hospitalization indicated; disabling; limiting self care ADL	Life-threatening consequences; urgent intervention indicated	Death

NIH Publication No. 09-5410
Revised June 2010
Reprinted June 2010
