# Supplementary figures and images for: Cost analysis of childhood hematopoietic stem cell transplantation in Sichuan, China
Source: Front Public Health. 2023 Mar 23;11:990181. doi: 10.3389/fpubh.2023.990181 (PMC10076710; doi:10.3389/fpubh.2023.990181)

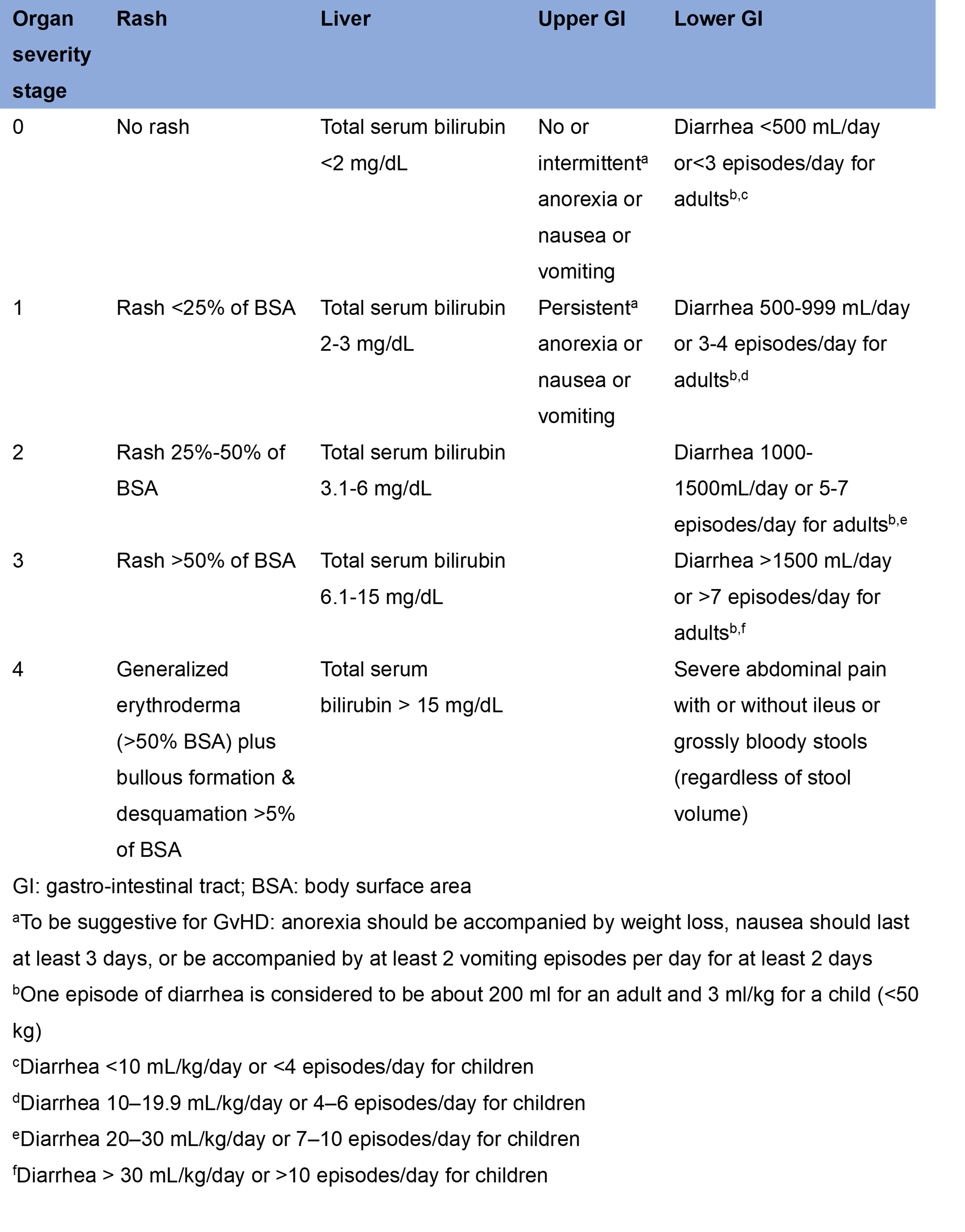

Supplement: Supplementary file 2 [file Image_1.TIF]

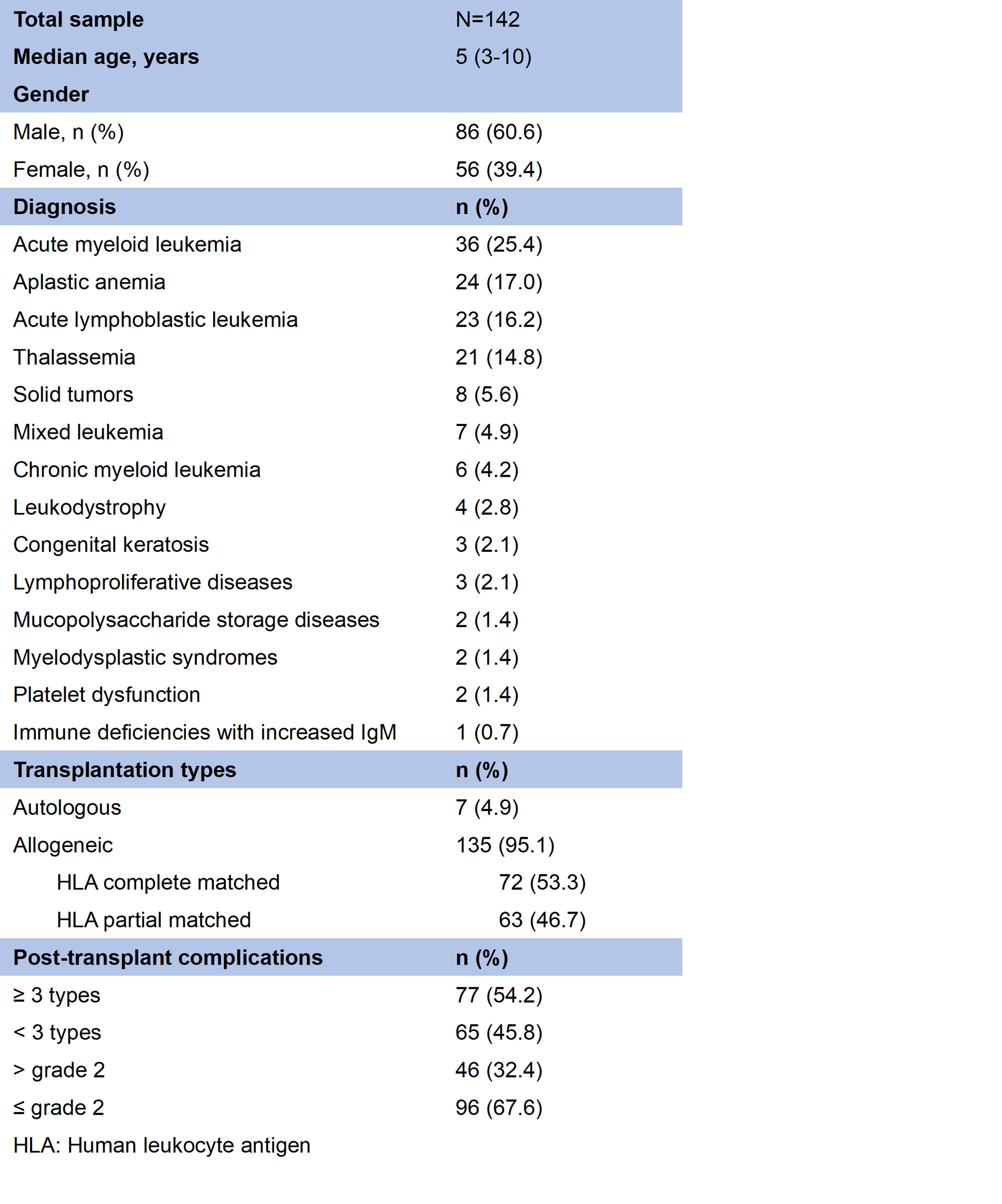

Supplement: Supplementary file 3 [file Image_2.TIF]

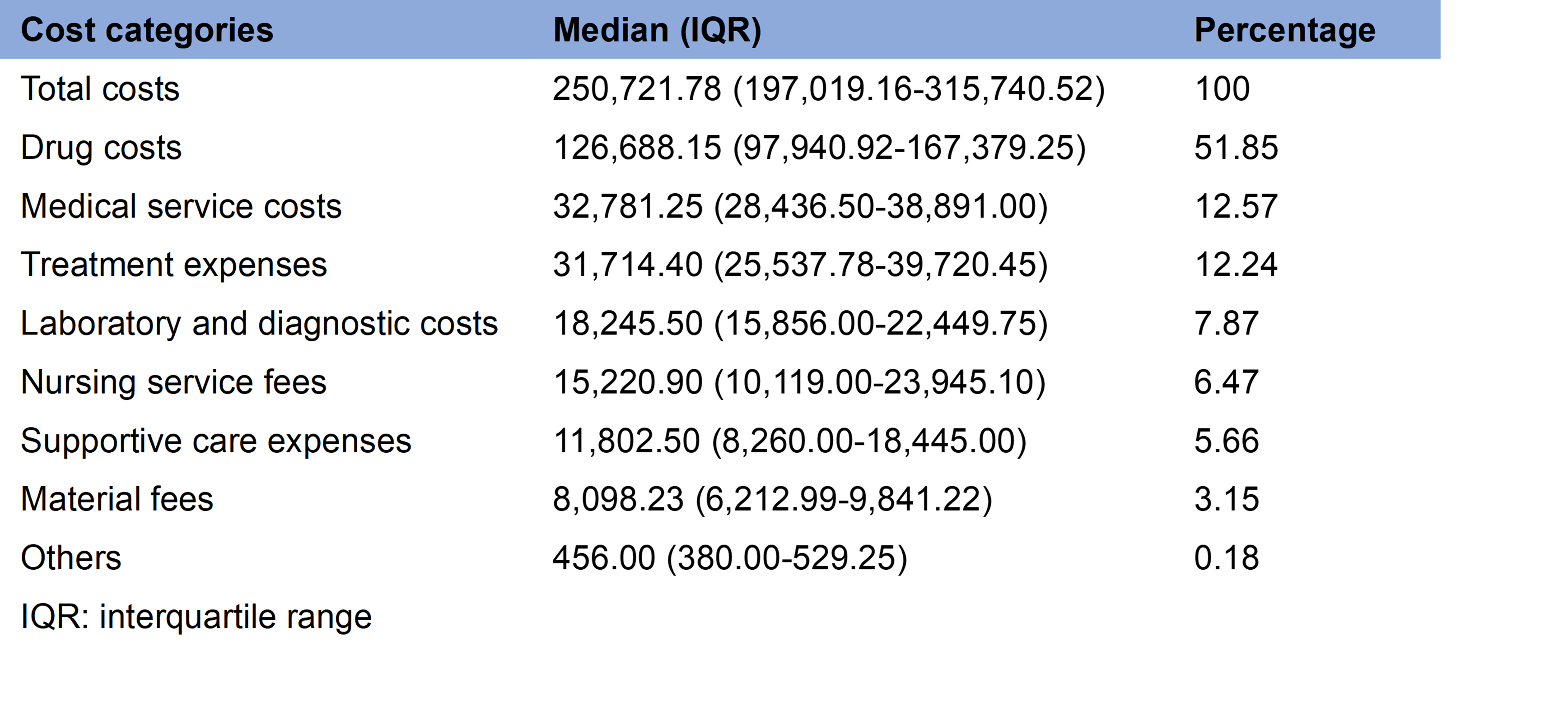

Supplement: Supplementary file 4 [file Image_3.TIF]

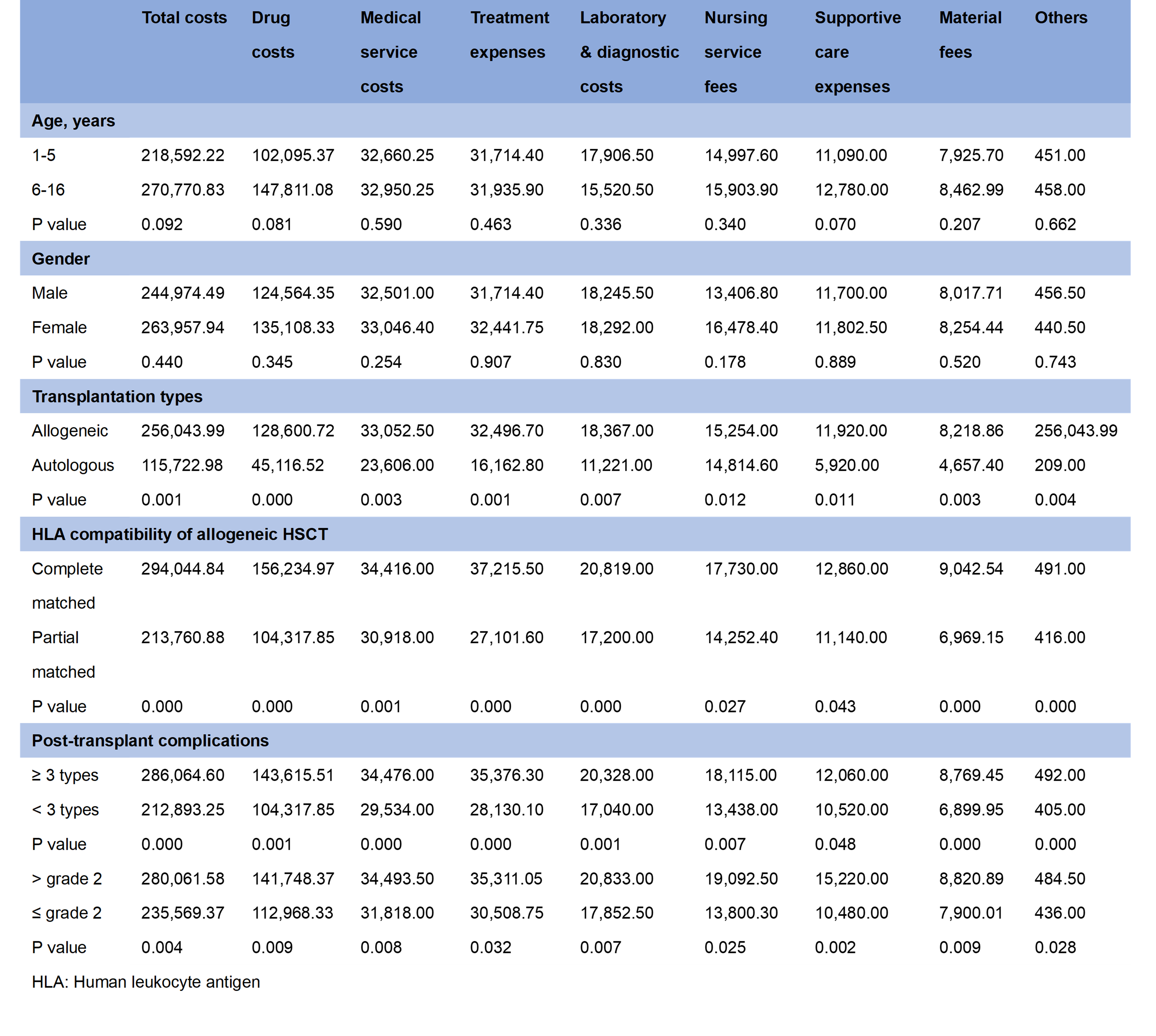

Supplement: Supplementary file 5 [file Image_4.TIF]
